# Supplementary material for: Genome-wide identification, characterization and gene expression of BES1 transcription factor family in grapevine (Vitis vinifera L.)
Source: Sci Rep. 2023 Jan 5;13:240. doi: 10.1038/s41598-022-24407-y (PMC9816167; doi:10.1038/s41598-022-24407-y)
Supplement: Supplementary file 3 — Supplementary Information. [file 41598_2022_24407_MOESM3_ESM.zip › Vvi_Vvi/Vitis_vinifera.PN40024.v4.dna_sm.toplevel.fa.vs.Vitis_vinifera.PN40024.v4.dna_sm.toplevel.fa.html/18.html]

|  |  |  |  |  |  |  |  |  |  |  |  |  |  |
| --- | --- | --- | --- | --- | --- | --- | --- | --- | --- | --- | --- | --- | --- |
| Duplication depth | Reference chromosome | Collinear blocks | | | | | | | | | | | |
| 0 | Vitvi18g04000\_t002 |  |  |  |  |  |  |  |  |  |  |  |  |
| 0 | Vitvi18g04001\_t001 |  |  |  |  |  |  |  |  |  |  |  |  |
| 1 | Vitvi18g00002\_t003 |  | Vitvi03g00289\_t001 |  |  |  |  |  |  |  |  |  |  |
| 1 | Vitvi18g00003\_t001 |  | \| \| |  |  |  |  |  |  |  |  |  |  |
| 1 | Vitvi18g00004\_t001 |  | \| \| |  |  |  |  |  |  |  |  |  |  |
| 1 | Vitvi18g00006\_t001 |  | \| \| |  |  |  |  |  |  |  |  |  |  |
| 1 | Vitvi18g00008\_t001 |  | Vitvi03g00274\_t001 |  |  |  |  |  |  |  |  |  |  |
| 1 | Vitvi18g00009\_t002 |  | \| \| |  |  |  |  |  |  |  |  |  |  |
| 1 | Vitvi18g00010\_t001 |  | \| \| |  |  |  |  |  |  |  |  |  |  |
| 1 | Vitvi18g02468\_t001 |  | \| \| |  |  |  |  |  |  |  |  |  |  |
| 1 | Vitvi18g00011\_t001 |  | \| \| |  |  |  |  |  |  |  |  |  |  |
| 1 | Vitvi18g04002\_t001 |  | \| \| |  |  |  |  |  |  |  |  |  |  |
| 1 | Vitvi18g02470\_t002 |  | \| \| |  |  |  |  |  |  |  |  |  |  |
| 1 | Vitvi18g00013\_t001 |  | \| \| |  |  |  |  |  |  |  |  |  |  |
| 1 | Vitvi18g00014\_t001 |  | \| \| |  |  |  |  |  |  |  |  |  |  |
| 1 | Vitvi18g00015\_t001 |  | \| \| |  |  |  |  |  |  |  |  |  |  |
| 1 | Vitvi18g00018\_t001 |  | \| \| |  |  |  |  |  |  |  |  |  |  |
| 1 | Vitvi18g02471\_t001 |  | \| \| |  |  |  |  |  |  |  |  |  |  |
| 1 | Vitvi18g00020\_t001 |  | \| \| |  |  |  |  |  |  |  |  |  |  |
| 1 | Vitvi18g02472\_t001 |  | \| \| |  |  |  |  |  |  |  |  |  |  |
| 1 | Vitvi18g00022\_t001 |  | \| \| |  |  |  |  |  |  |  |  |  |  |
| 1 | Vitvi18g00023\_t001 |  | Vitvi03g00267\_t001 |  |  |  |  |  |  |  |  |  |  |
| 1 | Vitvi18g02473\_t001 |  | \| \| |  |  |  |  |  |  |  |  |  |  |
| 1 | Vitvi18g04003\_t001 |  | \| \| |  |  |  |  |  |  |  |  |  |  |
| 1 | Vitvi18g04004\_t001 |  | \| \| |  |  |  |  |  |  |  |  |  |  |
| 1 | Vitvi18g00027\_t001 |  | \| \| |  |  |  |  |  |  |  |  |  |  |
| 1 | Vitvi18g04005\_t001 |  | \| \| |  |  |  |  |  |  |  |  |  |  |
| 1 | Vitvi18g00030\_t001 |  | \| \| |  |  |  |  |  |  |  |  |  |  |
| 1 | Vitvi18g00031\_t001 |  | \| \| |  |  |  |  |  |  |  |  |  |  |
| 1 | Vitvi18g04006\_t001 |  | \| \| |  |  |  |  |  |  |  |  |  |  |
| 1 | Vitvi18g04007\_t001 |  | \| \| |  |  |  |  |  |  |  |  |  |  |
| 1 | Vitvi18g00033\_t001 |  | \| \| |  |  |  |  |  |  |  |  |  |  |
| 1 | Vitvi18g02474\_t001 |  | \| \| |  |  |  |  |  |  |  |  |  |  |
| 1 | Vitvi18g00034\_t001 |  | \| \| |  |  |  |  |  |  |  |  |  |  |
| 1 | Vitvi18g00035\_t003 |  | \| \| |  |  |  |  |  |  |  |  |  |  |
| 1 | Vitvi18g00036\_t001 |  | \| \| |  |  |  |  |  |  |  |  |  |  |
| 1 | Vitvi18g00037\_t001 |  | \| \| |  |  |  |  |  |  |  |  |  |  |
| 1 | Vitvi18g04008\_t001 |  | \| \| |  |  |  |  |  |  |  |  |  |  |
| 1 | Vitvi18g04009\_t001 |  | \| \| |  |  |  |  |  |  |  |  |  |  |
| 1 | Vitvi18g00039\_t001 |  | Vitvi03g01475\_t003 |  |  |  |  |  |  |  |  |  |  |
| 1 | Vitvi18g00040\_t001 |  | \| \| |  |  |  |  |  |  |  |  |  |  |
| 1 | Vitvi18g00041\_t001 |  | \| \| |  |  |  |  |  |  |  |  |  |  |
| 1 | Vitvi18g00042\_t001 |  | Vitvi03g00263\_t001 |  |  |  |  |  |  |  |  |  |  |
| 1 | Vitvi18g04010\_t001 |  | \| \| |  |  |  |  |  |  |  |  |  |  |
| 1 | Vitvi18g00043\_t001 |  | Vitvi03g00262\_t001 |  |  |  |  |  |  |  |  |  |  |
| 1 | Vitvi18g02476\_t001 |  | \| \| |  |  |  |  |  |  |  |  |  |  |
| 1 | Vitvi18g02477\_t001 |  | \| \| |  |  |  |  |  |  |  |  |  |  |
| 1 | Vitvi18g02478\_t001 |  | \| \| |  |  |  |  |  |  |  |  |  |  |
| 1 | Vitvi18g02479\_t001 |  | \| \| |  |  |  |  |  |  |  |  |  |  |
| 1 | Vitvi18g00044\_t001 |  | \| \| |  |  |  |  |  |  |  |  |  |  |
| 1 | Vitvi18g00045\_t001 |  | \| \| |  |  |  |  |  |  |  |  |  |  |
| 1 | Vitvi18g00046\_t001 |  | \| \| |  |  |  |  |  |  |  |  |  |  |
| 1 | Vitvi18g00047\_t004 |  | \| \| |  |  |  |  |  |  |  |  |  |  |
| 1 | Vitvi18g00048\_t001 |  | \| \| |  |  |  |  |  |  |  |  |  |  |
| 1 | Vitvi18g02480\_t001 |  | \| \| |  |  |  |  |  |  |  |  |  |  |
| 1 | Vitvi18g00049\_t001 |  | \| \| |  |  |  |  |  |  |  |  |  |  |
| 1 | Vitvi18g04011\_t001 |  | \| \| |  |  |  |  |  |  |  |  |  |  |
| 1 | Vitvi18g04012\_t001 |  | \| \| |  |  |  |  |  |  |  |  |  |  |
| 1 | Vitvi18g00050\_t001 |  | \| \| |  |  |  |  |  |  |  |  |  |  |
| 1 | Vitvi18g00051\_t001 |  | Vitvi03g00253\_t001 |  |  |  |  |  |  |  |  |  |  |
| 1 | Vitvi18g00052\_t001 |  | \| \| |  |  |  |  |  |  |  |  |  |  |
| 1 | Vitvi18g00053\_t001 |  | \| \| |  |  |  |  |  |  |  |  |  |  |
| 1 | Vitvi18g00054\_t001 |  | \| \| |  |  |  |  |  |  |  |  |  |  |
| 1 | Vitvi18g00055\_t001 |  | \| \| |  |  |  |  |  |  |  |  |  |  |
| 2 | Vitvi18g00056\_t002 |  | Vitvi03g00247\_t001 |  | Vitvi07g01898\_t001 |  |  |  |  |  |  |  |  |
| 2 | Vitvi18g00057\_t001 |  | \| \| |  | \| \| |  |  |  |  |  |  |  |  |
| 2 | Vitvi18g00058\_t001 |  | Vitvi03g01473\_t001 |  | Vitvi07g02702\_t001 |  |  |  |  |  |  |  |  |
| 2 | Vitvi18g00059\_t001 |  | \| \| |  | \| \| |  |  |  |  |  |  |  |  |
| 2 | Vitvi18g00060\_t001 |  | \| \| |  | \| \| |  |  |  |  |  |  |  |  |
| 2 | Vitvi18g02483\_t001 |  | \| \| |  | \| \| |  |  |  |  |  |  |  |  |
| 2 | Vitvi18g00061\_t001 |  | \| \| |  | Vitvi07g01896\_t001 |  |  |  |  |  |  |  |  |
| 2 | Vitvi18g00064\_t001 |  | \| \| |  | \| \| |  |  |  |  |  |  |  |  |
| 2 | Vitvi18g00065\_t001 |  | \| \| |  | Vitvi07g04745\_t001 |  |  |  |  |  |  |  |  |
| 2 | Vitvi18g00067\_t001 |  | \| \| |  | Vitvi07g01894\_t001 |  |  |  |  |  |  |  |  |
| 2 | Vitvi18g00068\_t001 |  | \| \| |  | \| \| |  |  |  |  |  |  |  |  |
| 2 | Vitvi18g00069\_t001 |  | \| \| |  | Vitvi07g01891\_t001 |  |  |  |  |  |  |  |  |
| 2 | Vitvi18g00070\_t001 |  | \| \| |  | \| \| |  |  |  |  |  |  |  |  |
| 2 | Vitvi18g00071\_t001 |  | Vitvi03g00246\_t001 |  | Vitvi07g01890\_t001 |  |  |  |  |  |  |  |  |
| 2 | Vitvi18g04013\_t001 |  | \| \| |  | \| \| |  |  |  |  |  |  |  |  |
| 2 | Vitvi18g04014\_t001 |  | \| \| |  | \| \| |  |  |  |  |  |  |  |  |
| 2 | Vitvi18g00072\_t005 |  | \| \| |  | Vitvi07g01889\_t001 |  |  |  |  |  |  |  |  |
| 1 | Vitvi18g00073\_t001 |  | \| \| |  |  |  |  |  |  |  |  |  |  |
| 1 | Vitvi18g00075\_t001 |  | \| \| |  |  |  |  |  |  |  |  |  |  |
| 1 | Vitvi18g00076\_t001 |  | \| \| |  |  |  |  |  |  |  |  |  |  |
| 1 | Vitvi18g00077\_t001 |  | \| \| |  |  |  |  |  |  |  |  |  |  |
| 1 | Vitvi18g00078\_t001 |  | \| \| |  |  |  |  |  |  |  |  |  |  |
| 1 | Vitvi18g00079\_t001 |  | Vitvi03g00243\_t001 |  |  |  |  |  |  |  |  |  |  |
| 1 | Vitvi18g00080\_t001 |  | \| \| |  |  |  |  |  |  |  |  |  |  |
| 1 | Vitvi18g00081\_t001 |  | \| \| |  |  |  |  |  |  |  |  |  |  |
| 1 | Vitvi18g00082\_t001 |  | \| \| |  |  |  |  |  |  |  |  |  |  |
| 2 | Vitvi18g00084\_t001 |  | \| \| |  | Vitvi04g01578\_t001 |  |  |  |  |  |  |  |  |
| 2 | Vitvi18g02485\_t001 |  | \| \| |  | \| \| |  |  |  |  |  |  |  |  |
| 2 | Vitvi18g02486\_t001 |  | \| \| |  | Vitvi04g02203\_t004 |  |  |  |  |  |  |  |  |
| 2 | Vitvi18g00086\_t001 |  | \| \| |  | \| \| |  |  |  |  |  |  |  |  |
| 2 | Vitvi18g00087\_t001 |  | Vitvi03g00241\_t001 |  | \| \| |  |  |  |  |  |  |  |  |
| 2 | Vitvi18g00088\_t001 |  | Vitvi03g00240\_t001 |  | \| \| |  |  |  |  |  |  |  |  |
| 2 | Vitvi18g00089\_t001 |  | \| \| |  | \| \| |  |  |  |  |  |  |  |  |
| 2 | Vitvi18g00090\_t001 |  | Vitvi03g00239\_t001 |  | \| \| |  |  |  |  |  |  |  |  |
| 2 | Vitvi18g04015\_t001 |  | \| \| |  | \| \| |  |  |  |  |  |  |  |  |
| 2 | Vitvi18g00091\_t002 |  | Vitvi03g00238\_t001 |  | Vitvi04g01568\_t001 |  |  |  |  |  |  |  |  |
| 2 | Vitvi18g00092\_t001 |  | \| \| |  | \| \| |  |  |  |  |  |  |  |  |
| 2 | Vitvi18g00093\_t001 |  | Vitvi03g00233\_t001 |  | \| \| |  |  |  |  |  |  |  |  |
| 2 | Vitvi18g02488\_t001 |  | Vitvi03g00231\_t001 |  | \| \| |  |  |  |  |  |  |  |  |
| 2 | Vitvi18g02490\_t001 |  | \| \| |  | \| \| |  |  |  |  |  |  |  |  |
| 2 | Vitvi18g00095\_t001 |  | \| \| |  | Vitvi04g04448\_t001 |  |  |  |  |  |  |  |  |
| 2 | Vitvi18g00096\_t001 |  | \| \| |  | \| \| |  |  |  |  |  |  |  |  |
| 2 | Vitvi18g00098\_t001 |  | Vitvi03g00226\_t001 |  | \| \| |  |  |  |  |  |  |  |  |
| 1 | Vitvi18g00099\_t001 |  |  |  | \| \| |  |  |  |  |  |  |  |  |
| 1 | Vitvi18g00100\_t002 |  |  |  | \| \| |  |  |  |  |  |  |  |  |
| 1 | Vitvi18g00101\_t001 |  |  |  | \| \| |  |  |  |  |  |  |  |  |
| 1 | Vitvi18g00102\_t001 |  |  |  | \| \| |  |  |  |  |  |  |  |  |
| 1 | Vitvi18g04016\_t001 |  |  |  | \| \| |  |  |  |  |  |  |  |  |
| 2 | Vitvi18g00103\_t001 |  | Vitvi03g01427\_t001 |  | \| \| |  |  |  |  |  |  |  |  |
| 3 | Vitvi18g00105\_t001 |  | \| \| |  | \| \| |  | Vitvi04g01518\_t001 |  |  |  |  |  |  |
| 3 | Vitvi18g02493\_t001 |  | \| \| |  | Vitvi04g01555\_t001 |  | \| \| |  |  |  |  |  |  |
| 3 | Vitvi18g04017\_t001 |  | \| \| |  | \| \| |  | \| \| |  |  |  |  |  |  |
| 3 | Vitvi18g04018\_t001 |  | \| \| |  | \| \| |  | \| \| |  |  |  |  |  |  |
| 3 | Vitvi18g00106\_t001 |  | \| \| |  | \| \| |  | \| \| |  |  |  |  |  |  |
| 3 | Vitvi18g02494\_t001 |  | \| \| |  | Vitvi04g01554\_t001 |  | \| \| |  |  |  |  |  |  |
| 2 | Vitvi18g00107\_t001 |  | \| \| |  |  |  | \| \| |  |  |  |  |  |  |
| 2 | Vitvi18g00108\_t001 |  | \| \| |  |  |  | \| \| |  |  |  |  |  |  |
| 2 | Vitvi18g00109\_t001 |  | \| \| |  |  |  | \| \| |  |  |  |  |  |  |
| 2 | Vitvi18g00110\_t001 |  | \| \| |  |  |  | \| \| |  |  |  |  |  |  |
| 2 | Vitvi18g00111\_t001 |  | \| \| |  |  |  | \| \| |  |  |  |  |  |  |
| 2 | Vitvi18g00112\_t001 |  | \| \| |  |  |  | \| \| |  |  |  |  |  |  |
| 2 | Vitvi18g00113\_t001 |  | \| \| |  |  |  | \| \| |  |  |  |  |  |  |
| 2 | Vitvi18g00114\_t001 |  | Vitvi03g00202\_t001 |  |  |  | \| \| |  |  |  |  |  |  |
| 2 | Vitvi18g00115\_t001 |  | \| \| |  |  |  | \| \| |  |  |  |  |  |  |
| 2 | Vitvi18g00116\_t002 |  | \| \| |  |  |  | \| \| |  |  |  |  |  |  |
| 2 | Vitvi18g00117\_t001 |  | \| \| |  |  |  | \| \| |  |  |  |  |  |  |
| 2 | Vitvi18g02495\_t001 |  | \| \| |  |  |  | \| \| |  |  |  |  |  |  |
| 2 | Vitvi18g00118\_t001 |  | \| \| |  |  |  | Vitvi04g04420\_t001 |  |  |  |  |  |  |
| 2 | Vitvi18g00119\_t001 |  | Vitvi03g00204\_t001 |  |  |  | \| \| |  |  |  |  |  |  |
| 2 | Vitvi18g00120\_t002 |  | Vitvi03g00205\_t001 |  |  |  | \| \| |  |  |  |  |  |  |
| 2 | Vitvi18g02496\_t001 |  | \| \| |  |  |  | \| \| |  |  |  |  |  |  |
| 2 | Vitvi18g00121\_t001 |  | Vitvi03g00208\_t001 |  |  |  | \| \| |  |  |  |  |  |  |
| 2 | Vitvi18g00122\_t001 |  | \| \| |  |  |  | Vitvi04g02298\_t001 |  |  |  |  |  |  |
| 2 | Vitvi18g02497\_t001 |  | \| \| |  |  |  | \| \| |  |  |  |  |  |  |
| 2 | Vitvi18g00124\_t003 |  | \| \| |  |  |  | \| \| |  |  |  |  |  |  |
| 2 | Vitvi18g00127\_t001 |  | Vitvi03g01455\_t001 |  |  |  | \| \| |  |  |  |  |  |  |
| 2 | Vitvi18g04019\_t001 |  | Vitvi03g01457\_t001 |  |  |  | \| \| |  |  |  |  |  |  |
| 2 | Vitvi18g00128\_t001 |  | Vitvi03g00213\_t001 |  |  |  | \| \| |  |  |  |  |  |  |
| 2 | Vitvi18g00129\_t001 |  | \| \| |  |  |  | \| \| |  |  |  |  |  |  |
| 2 | Vitvi18g02499\_t001 |  | \| \| |  |  |  | \| \| |  |  |  |  |  |  |
| 2 | Vitvi18g00130\_t001 |  | Vitvi03g01463\_t001 |  |  |  | \| \| |  |  |  |  |  |  |
| 2 | Vitvi18g00131\_t001 |  | \| \| |  |  |  | \| \| |  |  |  |  |  |  |
| 2 | Vitvi18g00132\_t001.1.6037826c |  | \| \| |  |  |  | \| \| |  |  |  |  |  |  |
| 2 | Vitvi18g00133\_t001 |  | \| \| |  |  |  | \| \| |  |  |  |  |  |  |
| 2 | Vitvi18g00134\_t001 |  | \| \| |  |  |  | \| \| |  |  |  |  |  |  |
| 2 | Vitvi18g00135\_t001 |  | \| \| |  |  |  | \| \| |  |  |  |  |  |  |
| 2 | Vitvi18g02500\_t001 |  | \| \| |  |  |  | \| \| |  |  |  |  |  |  |
| 2 | Vitvi18g00136\_t001 |  | \| \| |  |  |  | \| \| |  |  |  |  |  |  |
| 2 | Vitvi18g00137\_t001 |  | Vitvi03g00217\_t001 |  |  |  | \| \| |  |  |  |  |  |  |
| 2 | Vitvi18g00138\_t002 |  | \| \| |  |  |  | \| \| |  |  |  |  |  |  |
| 2 | Vitvi18g00139\_t001 |  | \| \| |  |  |  | \| \| |  |  |  |  |  |  |
| 2 | Vitvi18g00140\_t001 |  | \| \| |  |  |  | \| \| |  |  |  |  |  |  |
| 2 | Vitvi18g00142\_t001 |  | \| \| |  |  |  | \| \| |  |  |  |  |  |  |
| 2 | Vitvi18g00143\_t001 |  | Vitvi03g00223\_t001 |  |  |  | \| \| |  |  |  |  |  |  |
| 1 | Vitvi18g00144\_t001 |  |  |  |  |  | \| \| |  |  |  |  |  |  |
| 1 | Vitvi18g04020\_t001 |  |  |  |  |  | \| \| |  |  |  |  |  |  |
| 1 | Vitvi18g00145\_t001 |  |  |  |  |  | \| \| |  |  |  |  |  |  |
| 1 | Vitvi18g00146\_t001 |  |  |  |  |  | \| \| |  |  |  |  |  |  |
| 1 | Vitvi18g00147\_t001 |  |  |  |  |  | Vitvi04g01519\_t001 |  |  |  |  |  |  |
| 1 | Vitvi18g00148\_t001 |  |  |  |  |  | \| \| |  |  |  |  |  |  |
| 1 | Vitvi18g04021\_t001 |  |  |  |  |  | \| \| |  |  |  |  |  |  |
| 1 | Vitvi18g02501\_t001 |  |  |  |  |  | \| \| |  |  |  |  |  |  |
| 1 | Vitvi18g00150\_t001 |  |  |  |  |  | \| \| |  |  |  |  |  |  |
| 1 | Vitvi18g00151\_t001 |  |  |  |  |  | \| \| |  |  |  |  |  |  |
| 1 | Vitvi18g00152\_t001 |  |  |  |  |  | \| \| |  |  |  |  |  |  |
| 1 | Vitvi18g00153\_t001 |  |  |  |  |  | \| \| |  |  |  |  |  |  |
| 1 | Vitvi18g04022\_t001 |  |  |  |  |  | \| \| |  |  |  |  |  |  |
| 1 | Vitvi18g04023\_t001 |  |  |  |  |  | \| \| |  |  |  |  |  |  |
| 2 | Vitvi18g00154\_t001 |  | Vitvi03g01416\_t001 |  |  |  | Vitvi04g04409\_t001 |  |  |  |  |  |  |
| 2 | Vitvi18g04024\_t001 |  | \| \| |  |  |  | \| \| |  |  |  |  |  |  |
| 2 | Vitvi18g00156\_t001 |  | \| \| |  |  |  | \| \| |  |  |  |  |  |  |
| 2 | Vitvi18g00157\_t001 |  | \| \| |  |  |  | \| \| |  |  |  |  |  |  |
| 2 | Vitvi18g00158\_t001 |  | \| \| |  |  |  | \| \| |  |  |  |  |  |  |
| 2 | Vitvi18g00159\_t001 |  | \| \| |  |  |  | Vitvi04g01530\_t002 |  |  |  |  |  |  |
| 2 | Vitvi18g00160\_t001 |  | \| \| |  |  |  | \| \| |  |  |  |  |  |  |
| 2 | Vitvi18g00161\_t001 |  | Vitvi03g00186\_t001 |  |  |  | \| \| |  |  |  |  |  |  |
| 2 | Vitvi18g00163\_t001 |  | Vitvi03g00185\_t001 |  |  |  | \| \| |  |  |  |  |  |  |
| 2 | Vitvi18g02503\_t001 |  | Vitvi03g04074\_t001 |  |  |  | \| \| |  |  |  |  |  |  |
| 2 | Vitvi18g00164\_t001 |  | \| \| |  |  |  | Vitvi04g04405\_t001 |  |  |  |  |  |  |
| 2 | Vitvi18g04025\_t001 |  | \| \| |  |  |  | \| \| |  |  |  |  |  |  |
| 2 | Vitvi18g00167\_t001 |  | \| \| |  |  |  | Vitvi04g04403\_t001 |  |  |  |  |  |  |
| 2 | Vitvi18g00168\_t001 |  | \| \| |  |  |  | \| \| |  |  |  |  |  |  |
| 2 | Vitvi18g00169\_t001 |  | \| \| |  |  |  | \| \| |  |  |  |  |  |  |
| 2 | Vitvi18g00170\_t001 |  | \| \| |  |  |  | \| \| |  |  |  |  |  |  |
| 2 | Vitvi18g00171\_t001 |  | Vitvi03g00180\_t001 |  |  |  | \| \| |  |  |  |  |  |  |
| 2 | Vitvi18g00172\_t001.3.6037826c |  | \| \| |  |  |  | Vitvi04g02185\_t001 |  |  |  |  |  |  |
| 2 | Vitvi18g04026\_t001 |  | \| \| |  |  |  | \| \| |  |  |  |  |  |  |
| 2 | Vitvi18g00173\_t001 |  | \| \| |  |  |  | \| \| |  |  |  |  |  |  |
| 2 | Vitvi18g00174\_t001 |  | \| \| |  |  |  | \| \| |  |  |  |  |  |  |
| 2 | Vitvi18g00175\_t001 |  | \| \| |  |  |  | \| \| |  |  |  |  |  |  |
| 2 | Vitvi18g00176\_t001 |  | Vitvi03g00173\_t001 |  |  |  | \| \| |  |  |  |  |  |  |
| 2 | Vitvi18g00177\_t001 |  | \| \| |  |  |  | \| \| |  |  |  |  |  |  |
| 2 | Vitvi18g00179\_t001 |  | \| \| |  |  |  | \| \| |  |  |  |  |  |  |
| 2 | Vitvi18g00180\_t001 |  | \| \| |  |  |  | \| \| |  |  |  |  |  |  |
| 2 | Vitvi18g00181\_t001 |  | \| \| |  |  |  | \| \| |  |  |  |  |  |  |
| 2 | Vitvi18g00182\_t001 |  | \| \| |  |  |  | \| \| |  |  |  |  |  |  |
| 2 | Vitvi18g04027\_t001 |  | \| \| |  |  |  | \| \| |  |  |  |  |  |  |
| 2 | Vitvi18g00183\_t001 |  | \| \| |  |  |  | \| \| |  |  |  |  |  |  |
| 2 | Vitvi18g00184\_t001 |  | \| \| |  |  |  | \| \| |  |  |  |  |  |  |
| 2 | Vitvi18g00185\_t001 |  | Vitvi03g04066\_t001 |  |  |  | \| \| |  |  |  |  |  |  |
| 2 | Vitvi18g00188\_t002 |  | Vitvi03g00161\_t001 |  |  |  | \| \| |  |  |  |  |  |  |
| 1 | Vitvi18g00189\_t001 |  |  |  |  |  | \| \| |  |  |  |  |  |  |
| 1 | Vitvi18g00190\_t001 |  |  |  |  |  | Vitvi04g02306\_t001 |  |  |  |  |  |  |
| 0 | Vitvi18g00191\_t001 |  |  |  |  |  |  |  |  |  |  |  |  |
| 0 | Vitvi18g00192\_t001 |  |  |  |  |  |  |  |  |  |  |  |  |
| 0 | Vitvi18g00193\_t001 |  |  |  |  |  |  |  |  |  |  |  |  |
| 0 | Vitvi18g02507\_t001 |  |  |  |  |  |  |  |  |  |  |  |  |
| 0 | Vitvi18g04028\_t001 |  |  |  |  |  |  |  |  |  |  |  |  |
| 0 | Vitvi18g02508\_t001 |  |  |  |  |  |  |  |  |  |  |  |  |
| 0 | Vitvi18g02509\_t001 |  |  |  |  |  |  |  |  |  |  |  |  |
| 0 | Vitvi18g02510\_t001 |  |  |  |  |  |  |  |  |  |  |  |  |
| 0 | Vitvi18g02511\_t001 |  |  |  |  |  |  |  |  |  |  |  |  |
| 0 | Vitvi18g00196\_t003 |  |  |  |  |  |  |  |  |  |  |  |  |
| 0 | Vitvi18g04029\_t001 |  |  |  |  |  |  |  |  |  |  |  |  |
| 0 | Vitvi18g02512\_t001 |  |  |  |  |  |  |  |  |  |  |  |  |
| 0 | Vitvi18g02513\_t001 |  |  |  |  |  |  |  |  |  |  |  |  |
| 0 | Vitvi18g00197\_t001 |  |  |  |  |  |  |  |  |  |  |  |  |
| 0 | Vitvi18g02514\_t001 |  |  |  |  |  |  |  |  |  |  |  |  |
| 0 | Vitvi18g04030\_t001 |  |  |  |  |  |  |  |  |  |  |  |  |
| 0 | Vitvi18g00200\_t001 |  |  |  |  |  |  |  |  |  |  |  |  |
| 0 | Vitvi18g00202\_t001 |  |  |  |  |  |  |  |  |  |  |  |  |
| 0 | Vitvi18g00203\_t001 |  |  |  |  |  |  |  |  |  |  |  |  |
| 0 | Vitvi18g00204\_t001 |  |  |  |  |  |  |  |  |  |  |  |  |
| 0 | Vitvi18g02517\_t001 |  |  |  |  |  |  |  |  |  |  |  |  |
| 0 | Vitvi18g04031\_t001 |  |  |  |  |  |  |  |  |  |  |  |  |
| 0 | Vitvi18g04032\_t001 |  |  |  |  |  |  |  |  |  |  |  |  |
| 0 | Vitvi18g04033\_t001 |  |  |  |  |  |  |  |  |  |  |  |  |
| 0 | Vitvi18g04034\_t001 |  |  |  |  |  |  |  |  |  |  |  |  |
| 0 | Vitvi18g02519\_t001 |  |  |  |  |  |  |  |  |  |  |  |  |
| 0 | Vitvi18g02521\_t001 |  |  |  |  |  |  |  |  |  |  |  |  |
| 0 | Vitvi18g04035\_t001 |  |  |  |  |  |  |  |  |  |  |  |  |
| 0 | Vitvi18g04036\_t001 |  |  |  |  |  |  |  |  |  |  |  |  |
| 0 | Vitvi18g04037\_t001 |  |  |  |  |  |  |  |  |  |  |  |  |
| 0 | Vitvi18g02522\_t001 |  |  |  |  |  |  |  |  |  |  |  |  |
| 0 | Vitvi18g04038\_t001 |  |  |  |  |  |  |  |  |  |  |  |  |
| 0 | Vitvi18g02524\_t001 |  |  |  |  |  |  |  |  |  |  |  |  |
| 0 | Vitvi18g00209\_t001 |  |  |  |  |  |  |  |  |  |  |  |  |
| 0 | Vitvi18g00211\_t001 |  |  |  |  |  |  |  |  |  |  |  |  |
| 0 | Vitvi18g00212\_t001 |  |  |  |  |  |  |  |  |  |  |  |  |
| 0 | Vitvi18g00213\_t001 |  |  |  |  |  |  |  |  |  |  |  |  |
| 0 | Vitvi18g00214\_t001 |  |  |  |  |  |  |  |  |  |  |  |  |
| 0 | Vitvi18g00215\_t001 |  |  |  |  |  |  |  |  |  |  |  |  |
| 0 | Vitvi18g02525\_t001 |  |  |  |  |  |  |  |  |  |  |  |  |
| 0 | Vitvi18g00216\_t001 |  |  |  |  |  |  |  |  |  |  |  |  |
| 0 | Vitvi18g00217\_t003 |  |  |  |  |  |  |  |  |  |  |  |  |
| 0 | Vitvi18g04039\_t001 |  |  |  |  |  |  |  |  |  |  |  |  |
| 0 | Vitvi18g00219\_t001 |  |  |  |  |  |  |  |  |  |  |  |  |
| 0 | Vitvi18g00220\_t001 |  |  |  |  |  |  |  |  |  |  |  |  |
| 0 | Vitvi18g00221\_t001 |  |  |  |  |  |  |  |  |  |  |  |  |
| 0 | Vitvi18g00222\_t001 |  |  |  |  |  |  |  |  |  |  |  |  |
| 0 | Vitvi18g00223\_t004 |  |  |  |  |  |  |  |  |  |  |  |  |
| 0 | Vitvi18g00224\_t001 |  |  |  |  |  |  |  |  |  |  |  |  |
| 0 | Vitvi18g00226\_t001 |  |  |  |  |  |  |  |  |  |  |  |  |
| 0 | Vitvi18g00227\_t001 |  |  |  |  |  |  |  |  |  |  |  |  |
| 0 | Vitvi18g00228\_t001 |  |  |  |  |  |  |  |  |  |  |  |  |
| 0 | Vitvi18g00230\_t001.1.6037826c |  |  |  |  |  |  |  |  |  |  |  |  |
| 0 | Vitvi18g00231\_t001 |  |  |  |  |  |  |  |  |  |  |  |  |
| 0 | Vitvi18g00232\_t001 |  |  |  |  |  |  |  |  |  |  |  |  |
| 0 | Vitvi18g00233\_t001 |  |  |  |  |  |  |  |  |  |  |  |  |
| 1 | Vitvi18g00234\_t001 |  | Vitvi07g02703\_t001 |  |  |  |  |  |  |  |  |  |  |
| 1 | Vitvi18g00235\_t001 |  | \| \| |  |  |  |  |  |  |  |  |  |  |
| 1 | Vitvi18g00236\_t001 |  | \| \| |  |  |  |  |  |  |  |  |  |  |
| 1 | Vitvi18g04040\_t001 |  | \| \| |  |  |  |  |  |  |  |  |  |  |
| 1 | Vitvi18g00237\_t001 |  | Vitvi07g04750\_t001 |  |  |  |  |  |  |  |  |  |  |
| 1 | Vitvi18g02526\_t001 |  | \| \| |  |  |  |  |  |  |  |  |  |  |
| 1 | Vitvi18g00238\_t001 |  | \| \| |  |  |  |  |  |  |  |  |  |  |
| 1 | Vitvi18g00239\_t001 |  | \| \| |  |  |  |  |  |  |  |  |  |  |
| 1 | Vitvi18g02527\_t001 |  | \| \| |  |  |  |  |  |  |  |  |  |  |
| 1 | Vitvi18g00240\_t001 |  | \| \| |  |  |  |  |  |  |  |  |  |  |
| 1 | Vitvi18g02528\_t001 |  | \| \| |  |  |  |  |  |  |  |  |  |  |
| 1 | Vitvi18g00241\_t001 |  | \| \| |  |  |  |  |  |  |  |  |  |  |
| 1 | Vitvi18g02529\_t001 |  | \| \| |  |  |  |  |  |  |  |  |  |  |
| 1 | Vitvi18g00242\_t001 |  | \| \| |  |  |  |  |  |  |  |  |  |  |
| 1 | Vitvi18g00243\_t001 |  | \| \| |  |  |  |  |  |  |  |  |  |  |
| 1 | Vitvi18g00244\_t001 |  | \| \| |  |  |  |  |  |  |  |  |  |  |
| 1 | Vitvi18g00245\_t001 |  | Vitvi07g01927\_t001 |  |  |  |  |  |  |  |  |  |  |
| 1 | Vitvi18g00246\_t001 |  | \| \| |  |  |  |  |  |  |  |  |  |  |
| 1 | Vitvi18g00247\_t001 |  | \| \| |  |  |  |  |  |  |  |  |  |  |
| 1 | Vitvi18g00248\_t001 |  | \| \| |  |  |  |  |  |  |  |  |  |  |
| 1 | Vitvi18g02530\_t001 |  | \| \| |  |  |  |  |  |  |  |  |  |  |
| 1 | Vitvi18g00250\_t001 |  | Vitvi07g01929\_t001 |  |  |  |  |  |  |  |  |  |  |
| 1 | Vitvi18g00251\_t001 |  | \| \| |  |  |  |  |  |  |  |  |  |  |
| 1 | Vitvi18g00252\_t001 |  | \| \| |  |  |  |  |  |  |  |  |  |  |
| 1 | Vitvi18g00253\_t001 |  | \| \| |  |  |  |  |  |  |  |  |  |  |
| 1 | Vitvi18g00254\_t001 |  | Vitvi07g01930\_t001 |  |  |  |  |  |  |  |  |  |  |
| 1 | Vitvi18g00255\_t001 |  | Vitvi07g01934\_t002 |  |  |  |  |  |  |  |  |  |  |
| 1 | Vitvi18g00256\_t001 |  | \| \| |  |  |  |  |  |  |  |  |  |  |
| 1 | Vitvi18g00257\_t001 |  | Vitvi07g01941\_t001 |  |  |  |  |  |  |  |  |  |  |
| 1 | Vitvi18g00258\_t001 |  | Vitvi07g01942\_t001 |  |  |  |  |  |  |  |  |  |  |
| 1 | Vitvi18g00260\_t001 |  | \| \| |  |  |  |  |  |  |  |  |  |  |
| 1 | Vitvi18g00261\_t001 |  | \| \| |  |  |  |  |  |  |  |  |  |  |
| 1 | Vitvi18g00262\_t001 |  | \| \| |  |  |  |  |  |  |  |  |  |  |
| 1 | Vitvi18g00263\_t001 |  | \| \| |  |  |  |  |  |  |  |  |  |  |
| 1 | Vitvi18g00264\_t001 |  | \| \| |  |  |  |  |  |  |  |  |  |  |
| 1 | Vitvi18g00265\_t001 |  | \| \| |  |  |  |  |  |  |  |  |  |  |
| 1 | Vitvi18g04041\_t001 |  | \| \| |  |  |  |  |  |  |  |  |  |  |
| 1 | Vitvi18g00267\_t002 |  | \| \| |  |  |  |  |  |  |  |  |  |  |
| 1 | Vitvi18g00268\_t001 |  | \| \| |  |  |  |  |  |  |  |  |  |  |
| 1 | Vitvi18g00269\_t001 |  | \| \| |  |  |  |  |  |  |  |  |  |  |
| 1 | Vitvi18g04042\_t001 |  | \| \| |  |  |  |  |  |  |  |  |  |  |
| 1 | Vitvi18g00270\_t001 |  | \| \| |  |  |  |  |  |  |  |  |  |  |
| 1 | Vitvi18g00271\_t001 |  | Vitvi07g01949\_t003 |  |  |  |  |  |  |  |  |  |  |
| 1 | Vitvi18g00272\_t001 |  | \| \| |  |  |  |  |  |  |  |  |  |  |
| 1 | Vitvi18g02532\_t001 |  | Vitvi07g01955\_t001 |  |  |  |  |  |  |  |  |  |  |
| 1 | Vitvi18g02533\_t003 |  | \| \| |  |  |  |  |  |  |  |  |  |  |
| 1 | Vitvi18g00273\_t001 |  | \| \| |  |  |  |  |  |  |  |  |  |  |
| 1 | Vitvi18g00274\_t001 |  | \| \| |  |  |  |  |  |  |  |  |  |  |
| 1 | Vitvi18g00275\_t002 |  | \| \| |  |  |  |  |  |  |  |  |  |  |
| 1 | Vitvi18g00276\_t001 |  | \| \| |  |  |  |  |  |  |  |  |  |  |
| 1 | Vitvi18g00277\_t001 |  | \| \| |  |  |  |  |  |  |  |  |  |  |
| 1 | Vitvi18g00279\_t002 |  | \| \| |  |  |  |  |  |  |  |  |  |  |
| 1 | Vitvi18g00280\_t001 |  | \| \| |  |  |  |  |  |  |  |  |  |  |
| 1 | Vitvi18g00281\_t001 |  | \| \| |  |  |  |  |  |  |  |  |  |  |
| 1 | Vitvi18g00282\_t001 |  | \| \| |  |  |  |  |  |  |  |  |  |  |
| 1 | Vitvi18g00283\_t001 |  | \| \| |  |  |  |  |  |  |  |  |  |  |
| 1 | Vitvi18g00284\_t001 |  | Vitvi07g01970\_t001 |  |  |  |  |  |  |  |  |  |  |
| 0 | Vitvi18g00285\_t001 |  |  |  |  |  |  |  |  |  |  |  |  |
| 0 | Vitvi18g00286\_t001 |  |  |  |  |  |  |  |  |  |  |  |  |
| 0 | Vitvi18g00287\_t001 |  |  |  |  |  |  |  |  |  |  |  |  |
| 0 | Vitvi18g00288\_t002 |  |  |  |  |  |  |  |  |  |  |  |  |
| 0 | Vitvi18g00289\_t001 |  |  |  |  |  |  |  |  |  |  |  |  |
| 0 | Vitvi18g00290\_t001 |  |  |  |  |  |  |  |  |  |  |  |  |
| 0 | Vitvi18g00291\_t001 |  |  |  |  |  |  |  |  |  |  |  |  |
| 0 | Vitvi18g00293\_t001 |  |  |  |  |  |  |  |  |  |  |  |  |
| 0 | Vitvi18g04043\_t001 |  |  |  |  |  |  |  |  |  |  |  |  |
| 0 | Vitvi18g04044\_t001 |  |  |  |  |  |  |  |  |  |  |  |  |
| 0 | Vitvi18g04045\_t001 |  |  |  |  |  |  |  |  |  |  |  |  |
| 0 | Vitvi18g00294\_t001 |  |  |  |  |  |  |  |  |  |  |  |  |
| 0 | Vitvi18g00295\_t001 |  |  |  |  |  |  |  |  |  |  |  |  |
| 0 | Vitvi18g00296\_t001 |  |  |  |  |  |  |  |  |  |  |  |  |
| 0 | Vitvi18g04046\_t001 |  |  |  |  |  |  |  |  |  |  |  |  |
| 0 | Vitvi18g00297\_t001 |  |  |  |  |  |  |  |  |  |  |  |  |
| 0 | Vitvi18g00298\_t001 |  |  |  |  |  |  |  |  |  |  |  |  |
| 0 | Vitvi18g00299\_t001 |  |  |  |  |  |  |  |  |  |  |  |  |
| 0 | Vitvi18g00300\_t001 |  |  |  |  |  |  |  |  |  |  |  |  |
| 0 | Vitvi18g00301\_t001 |  |  |  |  |  |  |  |  |  |  |  |  |
| 0 | Vitvi18g00302\_t001 |  |  |  |  |  |  |  |  |  |  |  |  |
| 0 | Vitvi18g00303\_t001 |  |  |  |  |  |  |  |  |  |  |  |  |
| 0 | Vitvi18g04047\_t001 |  |  |  |  |  |  |  |  |  |  |  |  |
| 0 | Vitvi18g02538\_t001 |  |  |  |  |  |  |  |  |  |  |  |  |
| 0 | Vitvi18g04048\_t001 |  |  |  |  |  |  |  |  |  |  |  |  |
| 0 | Vitvi18g02540\_t002 |  |  |  |  |  |  |  |  |  |  |  |  |
| 0 | Vitvi18g02541\_t001 |  |  |  |  |  |  |  |  |  |  |  |  |
| 0 | Vitvi18g02542\_t001 |  |  |  |  |  |  |  |  |  |  |  |  |
| 0 | Vitvi18g02543\_t001 |  |  |  |  |  |  |  |  |  |  |  |  |
| 0 | Vitvi18g00307\_t001 |  |  |  |  |  |  |  |  |  |  |  |  |
| 0 | Vitvi18g00308\_t003 |  |  |  |  |  |  |  |  |  |  |  |  |
| 0 | Vitvi18g00309\_t001 |  |  |  |  |  |  |  |  |  |  |  |  |
| 0 | Vitvi18g00310\_t001 |  |  |  |  |  |  |  |  |  |  |  |  |
| 0 | Vitvi18g00311\_t001 |  |  |  |  |  |  |  |  |  |  |  |  |
| 0 | Vitvi18g00312\_t001 |  |  |  |  |  |  |  |  |  |  |  |  |
| 0 | Vitvi18g00313\_t002 |  |  |  |  |  |  |  |  |  |  |  |  |
| 0 | Vitvi18g00315\_t001 |  |  |  |  |  |  |  |  |  |  |  |  |
| 0 | Vitvi18g04049\_t001 |  |  |  |  |  |  |  |  |  |  |  |  |
| 0 | Vitvi18g04050\_t001 |  |  |  |  |  |  |  |  |  |  |  |  |
| 0 | Vitvi18g00316\_t001 |  |  |  |  |  |  |  |  |  |  |  |  |
| 0 | Vitvi18g00317\_t001 |  |  |  |  |  |  |  |  |  |  |  |  |
| 0 | Vitvi18g04051\_t001 |  |  |  |  |  |  |  |  |  |  |  |  |
| 0 | Vitvi18g00318\_t001 |  |  |  |  |  |  |  |  |  |  |  |  |
| 0 | Vitvi18g00320\_t001 |  |  |  |  |  |  |  |  |  |  |  |  |
| 0 | Vitvi18g04052\_t001 |  |  |  |  |  |  |  |  |  |  |  |  |
| 0 | Vitvi18g00321\_t001 |  |  |  |  |  |  |  |  |  |  |  |  |
| 0 | Vitvi18g04053\_t001 |  |  |  |  |  |  |  |  |  |  |  |  |
| 0 | Vitvi18g00322\_t001 |  |  |  |  |  |  |  |  |  |  |  |  |
| 0 | Vitvi18g02546\_t001 |  |  |  |  |  |  |  |  |  |  |  |  |
| 0 | Vitvi18g00325\_t001 |  |  |  |  |  |  |  |  |  |  |  |  |
| 0 | Vitvi18g02547\_t001 |  |  |  |  |  |  |  |  |  |  |  |  |
| 0 | Vitvi18g02548\_t001 |  |  |  |  |  |  |  |  |  |  |  |  |
| 0 | Vitvi18g00326\_t001 |  |  |  |  |  |  |  |  |  |  |  |  |
| 0 | Vitvi18g04054\_t001 |  |  |  |  |  |  |  |  |  |  |  |  |
| 0 | Vitvi18g00328\_t001 |  |  |  |  |  |  |  |  |  |  |  |  |
| 0 | Vitvi18g04055\_t001 |  |  |  |  |  |  |  |  |  |  |  |  |
| 0 | Vitvi18g00329\_t001 |  |  |  |  |  |  |  |  |  |  |  |  |
| 0 | Vitvi18g00330\_t001 |  |  |  |  |  |  |  |  |  |  |  |  |
| 0 | Vitvi18g04056\_t001 |  |  |  |  |  |  |  |  |  |  |  |  |
| 0 | Vitvi18g02550\_t001 |  |  |  |  |  |  |  |  |  |  |  |  |
| 0 | Vitvi18g00331\_t001 |  |  |  |  |  |  |  |  |  |  |  |  |
| 0 | Vitvi18g00333\_t001 |  |  |  |  |  |  |  |  |  |  |  |  |
| 0 | Vitvi18g00334\_t001 |  |  |  |  |  |  |  |  |  |  |  |  |
| 0 | Vitvi18g00335\_t001 |  |  |  |  |  |  |  |  |  |  |  |  |
| 0 | Vitvi18g04057\_t001 |  |  |  |  |  |  |  |  |  |  |  |  |
| 0 | Vitvi18g00336\_t001 |  |  |  |  |  |  |  |  |  |  |  |  |
| 0 | Vitvi18g04058\_t001 |  |  |  |  |  |  |  |  |  |  |  |  |
| 0 | Vitvi18g04059\_t001 |  |  |  |  |  |  |  |  |  |  |  |  |
| 0 | Vitvi18g00337\_t001 |  |  |  |  |  |  |  |  |  |  |  |  |
| 0 | Vitvi18g00338\_t001 |  |  |  |  |  |  |  |  |  |  |  |  |
| 0 | Vitvi18g00339\_t001 |  |  |  |  |  |  |  |  |  |  |  |  |
| 0 | Vitvi18g04060\_t001 |  |  |  |  |  |  |  |  |  |  |  |  |
| 0 | Vitvi18g00341\_t001 |  |  |  |  |  |  |  |  |  |  |  |  |
| 0 | Vitvi18g00342\_t001 |  |  |  |  |  |  |  |  |  |  |  |  |
| 0 | Vitvi18g04061\_t001 |  |  |  |  |  |  |  |  |  |  |  |  |
| 0 | Vitvi18g00343\_t001 |  |  |  |  |  |  |  |  |  |  |  |  |
| 0 | Vitvi18g00344\_t001 |  |  |  |  |  |  |  |  |  |  |  |  |
| 0 | Vitvi18g00345\_t001 |  |  |  |  |  |  |  |  |  |  |  |  |
| 1 | Vitvi18g00346\_t001 |  | Vitvi07g01807\_t001 |  |  |  |  |  |  |  |  |  |  |
| 1 | Vitvi18g04062\_t001 |  | \| \| |  |  |  |  |  |  |  |  |  |  |
| 1 | Vitvi18g00347\_t001 |  | \| \| |  |  |  |  |  |  |  |  |  |  |
| 1 | Vitvi18g04063\_t002 |  | \| \| |  |  |  |  |  |  |  |  |  |  |
| 1 | Vitvi18g00348\_t001 |  | \| \| |  |  |  |  |  |  |  |  |  |  |
| 1 | Vitvi18g00349\_t001 |  | \| \| |  |  |  |  |  |  |  |  |  |  |
| 1 | Vitvi18g00350\_t001 |  | \| \| |  |  |  |  |  |  |  |  |  |  |
| 1 | Vitvi18g04064\_t001 |  | \| \| |  |  |  |  |  |  |  |  |  |  |
| 1 | Vitvi18g00351\_t001 |  | \| \| |  |  |  |  |  |  |  |  |  |  |
| 1 | Vitvi18g00352\_t002 |  | \| \| |  |  |  |  |  |  |  |  |  |  |
| 1 | Vitvi18g00353\_t001 |  | \| \| |  |  |  |  |  |  |  |  |  |  |
| 1 | Vitvi18g04065\_t001 |  | \| \| |  |  |  |  |  |  |  |  |  |  |
| 1 | Vitvi18g04066\_t001 |  | \| \| |  |  |  |  |  |  |  |  |  |  |
| 1 | Vitvi18g00355\_t001 |  | \| \| |  |  |  |  |  |  |  |  |  |  |
| 1 | Vitvi18g02556\_t001 |  | \| \| |  |  |  |  |  |  |  |  |  |  |
| 1 | Vitvi18g04067\_t001 |  | \| \| |  |  |  |  |  |  |  |  |  |  |
| 1 | Vitvi18g00356\_t001 |  | \| \| |  |  |  |  |  |  |  |  |  |  |
| 1 | Vitvi18g02557\_t001 |  | \| \| |  |  |  |  |  |  |  |  |  |  |
| 1 | Vitvi18g02558\_t001 |  | \| \| |  |  |  |  |  |  |  |  |  |  |
| 1 | Vitvi18g00357\_t001 |  | \| \| |  |  |  |  |  |  |  |  |  |  |
| 1 | Vitvi18g00358\_t002 |  | Vitvi07g01802\_t001 |  |  |  |  |  |  |  |  |  |  |
| 1 | Vitvi18g00359\_t001 |  | Vitvi07g01800\_t001 |  |  |  |  |  |  |  |  |  |  |
| 1 | Vitvi18g04068\_t001 |  | \| \| |  |  |  |  |  |  |  |  |  |  |
| 1 | Vitvi18g04069\_t001 |  | \| \| |  |  |  |  |  |  |  |  |  |  |
| 1 | Vitvi18g00360\_t001 |  | Vitvi07g01795\_t001 |  |  |  |  |  |  |  |  |  |  |
| 1 | Vitvi18g04070\_t001 |  | \| \| |  |  |  |  |  |  |  |  |  |  |
| 1 | Vitvi18g02559\_t001 |  | Vitvi07g01794\_t001 |  |  |  |  |  |  |  |  |  |  |
| 1 | Vitvi18g00361\_t001 |  | Vitvi07g01792\_t001 |  |  |  |  |  |  |  |  |  |  |
| 1 | Vitvi18g04071\_t001 |  | \| \| |  |  |  |  |  |  |  |  |  |  |
| 1 | Vitvi18g02563\_t001 |  | \| \| |  |  |  |  |  |  |  |  |  |  |
| 1 | Vitvi18g00362\_t001 |  | Vitvi07g01783\_t002 |  |  |  |  |  |  |  |  |  |  |
| 1 | Vitvi18g02565\_t002 |  | \| \| |  |  |  |  |  |  |  |  |  |  |
| 1 | Vitvi18g00363\_t001 |  | Vitvi07g01779\_t001 |  |  |  |  |  |  |  |  |  |  |
| 0 | Vitvi18g04072\_t001 |  |  |  |  |  |  |  |  |  |  |  |  |
| 0 | Vitvi18g00365\_t001 |  |  |  |  |  |  |  |  |  |  |  |  |
| 0 | Vitvi18g00367\_t001 |  |  |  |  |  |  |  |  |  |  |  |  |
| 0 | Vitvi18g00368\_t003 |  |  |  |  |  |  |  |  |  |  |  |  |
| 0 | Vitvi18g04073\_t001 |  |  |  |  |  |  |  |  |  |  |  |  |
| 1 | Vitvi18g02569\_t001 |  | Vitvi07g04543\_t001 |  |  |  |  |  |  |  |  |  |  |
| 1 | Vitvi18g02570\_t001 |  | Vitvi07g02536\_t001 |  |  |  |  |  |  |  |  |  |  |
| 1 | Vitvi18g00371\_t001 |  | Vitvi07g01351\_t001 |  |  |  |  |  |  |  |  |  |  |
| 1 | Vitvi18g02571\_t001 |  | \| \| |  |  |  |  |  |  |  |  |  |  |
| 1 | Vitvi18g00372\_t001 |  | \| \| |  |  |  |  |  |  |  |  |  |  |
| 1 | Vitvi18g04074\_t001 |  | \| \| |  |  |  |  |  |  |  |  |  |  |
| 1 | Vitvi18g00373\_t001 |  | \| \| |  |  |  |  |  |  |  |  |  |  |
| 1 | Vitvi18g00374\_t001 |  | \| \| |  |  |  |  |  |  |  |  |  |  |
| 1 | Vitvi18g00375\_t001 |  | \| \| |  |  |  |  |  |  |  |  |  |  |
| 1 | Vitvi18g00376\_t001 |  | \| \| |  |  |  |  |  |  |  |  |  |  |
| 1 | Vitvi18g04075\_t001 |  | \| \| |  |  |  |  |  |  |  |  |  |  |
| 1 | Vitvi18g00378\_t001 |  | \| \| |  |  |  |  |  |  |  |  |  |  |
| 1 | Vitvi18g00381\_t001 |  | Vitvi07g03030\_t001 |  |  |  |  |  |  |  |  |  |  |
| 1 | Vitvi18g04076\_t001 |  | \| \| |  |  |  |  |  |  |  |  |  |  |
| 1 | Vitvi18g00382\_t001 |  | \| \| |  |  |  |  |  |  |  |  |  |  |
| 1 | Vitvi18g00384\_t001 |  | Vitvi07g01365\_t001 |  |  |  |  |  |  |  |  |  |  |
| 1 | Vitvi18g00385\_t001 |  | Vitvi07g02540\_t001 |  |  |  |  |  |  |  |  |  |  |
| 1 | Vitvi18g04077\_t001 |  | \| \| |  |  |  |  |  |  |  |  |  |  |
| 1 | Vitvi18g00386\_t001 |  | \| \| |  |  |  |  |  |  |  |  |  |  |
| 1 | Vitvi18g00387\_t001 |  | \| \| |  |  |  |  |  |  |  |  |  |  |
| 1 | Vitvi18g00388\_t001 |  | \| \| |  |  |  |  |  |  |  |  |  |  |
| 1 | Vitvi18g00390\_t001 |  | Vitvi07g01363\_t001 |  |  |  |  |  |  |  |  |  |  |
| 1 | Vitvi18g00391\_t001 |  | Vitvi07g01359\_t001 |  |  |  |  |  |  |  |  |  |  |
| 1 | Vitvi18g00392\_t001 |  | Vitvi07g01358\_t001 |  |  |  |  |  |  |  |  |  |  |
| 1 | Vitvi18g00393\_t001 |  | \| \| |  |  |  |  |  |  |  |  |  |  |
| 1 | Vitvi18g00394\_t001 |  | Vitvi07g04557\_t001 |  |  |  |  |  |  |  |  |  |  |
| 1 | Vitvi18g02573\_t001 |  | \| \| |  |  |  |  |  |  |  |  |  |  |
| 1 | Vitvi18g00395\_t001 |  | \| \| |  |  |  |  |  |  |  |  |  |  |
| 1 | Vitvi18g00396\_t001 |  | \| \| |  |  |  |  |  |  |  |  |  |  |
| 1 | Vitvi18g00397\_t001 |  | \| \| |  |  |  |  |  |  |  |  |  |  |
| 1 | Vitvi18g00398\_t001 |  | \| \| |  |  |  |  |  |  |  |  |  |  |
| 1 | Vitvi18g04078\_t001 |  | \| \| |  |  |  |  |  |  |  |  |  |  |
| 1 | Vitvi18g00400\_t001 |  | \| \| |  |  |  |  |  |  |  |  |  |  |
| 1 | Vitvi18g00401\_t001 |  | \| \| |  |  |  |  |  |  |  |  |  |  |
| 1 | Vitvi18g00402\_t001 |  | \| \| |  |  |  |  |  |  |  |  |  |  |
| 1 | Vitvi18g00403\_t001 |  | \| \| |  |  |  |  |  |  |  |  |  |  |
| 1 | Vitvi18g04079\_t001 |  | \| \| |  |  |  |  |  |  |  |  |  |  |
| 1 | Vitvi18g00405\_t001 |  | \| \| |  |  |  |  |  |  |  |  |  |  |
| 1 | Vitvi18g00406\_t001 |  | \| \| |  |  |  |  |  |  |  |  |  |  |
| 1 | Vitvi18g00407\_t001 |  | \| \| |  |  |  |  |  |  |  |  |  |  |
| 1 | Vitvi18g04080\_t001 |  | \| \| |  |  |  |  |  |  |  |  |  |  |
| 1 | Vitvi18g00408\_t001 |  | \| \| |  |  |  |  |  |  |  |  |  |  |
| 1 | Vitvi18g02574\_t002 |  | Vitvi07g04569\_t001 |  |  |  |  |  |  |  |  |  |  |
| 1 | Vitvi18g00409\_t001 |  | \| \| |  |  |  |  |  |  |  |  |  |  |
| 1 | Vitvi18g00410\_t001 |  | \| \| |  |  |  |  |  |  |  |  |  |  |
| 1 | Vitvi18g00411\_t001 |  | \| \| |  |  |  |  |  |  |  |  |  |  |
| 1 | Vitvi18g00412\_t002 |  | \| \| |  |  |  |  |  |  |  |  |  |  |
| 1 | Vitvi18g00413\_t001 |  | \| \| |  |  |  |  |  |  |  |  |  |  |
| 1 | Vitvi18g00414\_t001 |  | \| \| |  |  |  |  |  |  |  |  |  |  |
| 1 | Vitvi18g04081\_t001 |  | \| \| |  |  |  |  |  |  |  |  |  |  |
| 1 | Vitvi18g00416\_t001 |  | \| \| |  |  |  |  |  |  |  |  |  |  |
| 1 | Vitvi18g00417\_t001 |  | \| \| |  |  |  |  |  |  |  |  |  |  |
| 1 | Vitvi18g04082\_t001 |  | Vitvi07g01394\_t001 |  |  |  |  |  |  |  |  |  |  |
| 1 | Vitvi18g04083\_t001 |  | \| \| |  |  |  |  |  |  |  |  |  |  |
| 1 | Vitvi18g04084\_t001 |  | \| \| |  |  |  |  |  |  |  |  |  |  |
| 1 | Vitvi18g04085\_t001 |  | \| \| |  |  |  |  |  |  |  |  |  |  |
| 1 | Vitvi18g00420\_t001 |  | Vitvi07g03041\_t001 |  |  |  |  |  |  |  |  |  |  |
| 1 | Vitvi18g04086\_t001 |  | \| \| |  |  |  |  |  |  |  |  |  |  |
| 1 | Vitvi18g04087\_t001 |  | \| \| |  |  |  |  |  |  |  |  |  |  |
| 1 | Vitvi18g04088\_t001 |  | \| \| |  |  |  |  |  |  |  |  |  |  |
| 1 | Vitvi18g00421\_t001 |  | \| \| |  |  |  |  |  |  |  |  |  |  |
| 1 | Vitvi18g00422\_t001 |  | \| \| |  |  |  |  |  |  |  |  |  |  |
| 1 | Vitvi18g00424\_t001 |  | \| \| |  |  |  |  |  |  |  |  |  |  |
| 1 | Vitvi18g00425\_t001 |  | \| \| |  |  |  |  |  |  |  |  |  |  |
| 1 | Vitvi18g04089\_t001 |  | \| \| |  |  |  |  |  |  |  |  |  |  |
| 1 | Vitvi18g04090\_t001 |  | \| \| |  |  |  |  |  |  |  |  |  |  |
| 1 | Vitvi18g02581\_t001 |  | Vitvi07g01396\_t001 |  |  |  |  |  |  |  |  |  |  |
| 1 | Vitvi18g02582\_t001 |  | \| \| |  |  |  |  |  |  |  |  |  |  |
| 1 | Vitvi18g00427\_t001 |  | \| \| |  |  |  |  |  |  |  |  |  |  |
| 1 | Vitvi18g00430\_t001 |  | \| \| |  |  |  |  |  |  |  |  |  |  |
| 1 | Vitvi18g00431\_t001 |  | \| \| |  |  |  |  |  |  |  |  |  |  |
| 1 | Vitvi18g04091\_t001 |  | \| \| |  |  |  |  |  |  |  |  |  |  |
| 1 | Vitvi18g00432\_t001 |  | \| \| |  |  |  |  |  |  |  |  |  |  |
| 1 | Vitvi18g02584\_t001 |  | \| \| |  |  |  |  |  |  |  |  |  |  |
| 1 | Vitvi18g00433\_t001 |  | \| \| |  |  |  |  |  |  |  |  |  |  |
| 1 | Vitvi18g04092\_t001 |  | \| \| |  |  |  |  |  |  |  |  |  |  |
| 1 | Vitvi18g00434\_t001 |  | \| \| |  |  |  |  |  |  |  |  |  |  |
| 1 | Vitvi18g00435\_t002 |  | Vitvi07g03046\_t001 |  |  |  |  |  |  |  |  |  |  |
| 1 | Vitvi18g00436\_t001 |  | Vitvi07g03049\_t001 |  |  |  |  |  |  |  |  |  |  |
| 1 | Vitvi18g00437\_t001 |  | \| \| |  |  |  |  |  |  |  |  |  |  |
| 1 | Vitvi18g00438\_t001 |  | Vitvi07g01501\_t001 |  |  |  |  |  |  |  |  |  |  |
| 1 | Vitvi18g00439\_t001 |  | \| \| |  |  |  |  |  |  |  |  |  |  |
| 1 | Vitvi18g00440\_t001 |  | Vitvi07g01500\_t001 |  |  |  |  |  |  |  |  |  |  |
| 1 | Vitvi18g00441\_t001 |  | \| \| |  |  |  |  |  |  |  |  |  |  |
| 1 | Vitvi18g00442\_t001 |  | Vitvi07g01498\_t001 |  |  |  |  |  |  |  |  |  |  |
| 1 | Vitvi18g00443\_t001 |  | \| \| |  |  |  |  |  |  |  |  |  |  |
| 1 | Vitvi18g00444\_t001 |  | \| \| |  |  |  |  |  |  |  |  |  |  |
| 1 | Vitvi18g00445\_t001 |  | \| \| |  |  |  |  |  |  |  |  |  |  |
| 1 | Vitvi18g00446\_t001 |  | \| \| |  |  |  |  |  |  |  |  |  |  |
| 1 | Vitvi18g02585\_t001 |  | \| \| |  |  |  |  |  |  |  |  |  |  |
| 1 | Vitvi18g00447\_t001 |  | \| \| |  |  |  |  |  |  |  |  |  |  |
| 1 | Vitvi18g00448\_t001 |  | Vitvi07g01488\_t001 |  |  |  |  |  |  |  |  |  |  |
| 1 | Vitvi18g00449\_t001 |  | \| \| |  |  |  |  |  |  |  |  |  |  |
| 1 | Vitvi18g00452\_t001 |  | \| \| |  |  |  |  |  |  |  |  |  |  |
| 1 | Vitvi18g00454\_t001 |  | \| \| |  |  |  |  |  |  |  |  |  |  |
| 1 | Vitvi18g04093\_t001 |  | \| \| |  |  |  |  |  |  |  |  |  |  |
| 1 | Vitvi18g04094\_t001 |  | \| \| |  |  |  |  |  |  |  |  |  |  |
| 1 | Vitvi18g02588\_t001 |  | \| \| |  |  |  |  |  |  |  |  |  |  |
| 1 | Vitvi18g04095\_t001 |  | \| \| |  |  |  |  |  |  |  |  |  |  |
| 1 | Vitvi18g00456\_t001 |  | \| \| |  |  |  |  |  |  |  |  |  |  |
| 1 | Vitvi18g00457\_t001 |  | \| \| |  |  |  |  |  |  |  |  |  |  |
| 1 | Vitvi18g02589\_t002 |  | Vitvi07g02602\_t002 |  |  |  |  |  |  |  |  |  |  |
| 1 | Vitvi18g04096\_t001 |  | \| \| |  |  |  |  |  |  |  |  |  |  |
| 1 | Vitvi18g00462\_t001 |  | Vitvi07g01476\_t001 |  |  |  |  |  |  |  |  |  |  |
| 1 | Vitvi18g00463\_t001 |  | Vitvi07g01475\_t001 |  |  |  |  |  |  |  |  |  |  |
| 1 | Vitvi18g00464\_t001 |  | Vitvi07g04590\_t001 |  |  |  |  |  |  |  |  |  |  |
| 1 | Vitvi18g00466\_t001 |  | \| \| |  |  |  |  |  |  |  |  |  |  |
| 1 | Vitvi18g00467\_t001 |  | \| \| |  |  |  |  |  |  |  |  |  |  |
| 1 | Vitvi18g04097\_t001 |  | \| \| |  |  |  |  |  |  |  |  |  |  |
| 1 | Vitvi18g00469\_t001 |  | Vitvi07g01471\_t001 |  |  |  |  |  |  |  |  |  |  |
| 1 | Vitvi18g00470\_t001 |  | Vitvi07g04591\_t001 |  |  |  |  |  |  |  |  |  |  |
| 1 | Vitvi18g00472\_t001 |  | \| \| |  |  |  |  |  |  |  |  |  |  |
| 1 | Vitvi18g00473\_t001 |  | \| \| |  |  |  |  |  |  |  |  |  |  |
| 1 | Vitvi18g02592\_t001 |  | \| \| |  |  |  |  |  |  |  |  |  |  |
| 1 | Vitvi18g00474\_t001 |  | \| \| |  |  |  |  |  |  |  |  |  |  |
| 1 | Vitvi18g00475\_t001 |  | \| \| |  |  |  |  |  |  |  |  |  |  |
| 1 | Vitvi18g00476\_t001 |  | \| \| |  |  |  |  |  |  |  |  |  |  |
| 1 | Vitvi18g00479\_t001 |  | \| \| |  |  |  |  |  |  |  |  |  |  |
| 1 | Vitvi18g04098\_t001 |  | \| \| |  |  |  |  |  |  |  |  |  |  |
| 1 | Vitvi18g00480\_t001 |  | \| \| |  |  |  |  |  |  |  |  |  |  |
| 1 | Vitvi18g00482\_t001 |  | \| \| |  |  |  |  |  |  |  |  |  |  |
| 1 | Vitvi18g00483\_t001 |  | \| \| |  |  |  |  |  |  |  |  |  |  |
| 1 | Vitvi18g04099\_t001 |  | \| \| |  |  |  |  |  |  |  |  |  |  |
| 1 | Vitvi18g00485\_t001.1.6037826c |  | \| \| |  |  |  |  |  |  |  |  |  |  |
| 1 | Vitvi18g02597\_t001 |  | \| \| |  |  |  |  |  |  |  |  |  |  |
| 1 | Vitvi18g00486\_t001 |  | \| \| |  |  |  |  |  |  |  |  |  |  |
| 1 | Vitvi18g00487\_t001 |  | \| \| |  |  |  |  |  |  |  |  |  |  |
| 1 | Vitvi18g00488\_t001 |  | Vitvi07g01469\_t001 |  |  |  |  |  |  |  |  |  |  |
| 1 | Vitvi18g04100\_t001 |  | \| \| |  |  |  |  |  |  |  |  |  |  |
| 1 | Vitvi18g00489\_t001 |  | Vitvi07g01468\_t001 |  |  |  |  |  |  |  |  |  |  |
| 1 | Vitvi18g00490\_t001 |  | \| \| |  |  |  |  |  |  |  |  |  |  |
| 1 | Vitvi18g00491\_t001 |  | \| \| |  |  |  |  |  |  |  |  |  |  |
| 1 | Vitvi18g00492\_t001 |  | Vitvi07g04592\_t001 |  |  |  |  |  |  |  |  |  |  |
| 1 | Vitvi18g00493\_t001 |  | Vitvi07g01465\_t001 |  |  |  |  |  |  |  |  |  |  |
| 1 | Vitvi18g00494\_t001 |  | \| \| |  |  |  |  |  |  |  |  |  |  |
| 1 | Vitvi18g04101\_t001 |  | \| \| |  |  |  |  |  |  |  |  |  |  |
| 1 | Vitvi18g00495\_t001 |  | \| \| |  |  |  |  |  |  |  |  |  |  |
| 1 | Vitvi18g00496\_t001 |  | \| \| |  |  |  |  |  |  |  |  |  |  |
| 1 | Vitvi18g00498\_t001 |  | \| \| |  |  |  |  |  |  |  |  |  |  |
| 1 | Vitvi18g04102\_t001 |  | \| \| |  |  |  |  |  |  |  |  |  |  |
| 1 | Vitvi18g04103\_t001 |  | \| \| |  |  |  |  |  |  |  |  |  |  |
| 1 | Vitvi18g00499\_t001 |  | Vitvi07g01460\_t001 |  |  |  |  |  |  |  |  |  |  |
| 1 | Vitvi18g00500\_t001 |  | \| \| |  |  |  |  |  |  |  |  |  |  |
| 1 | Vitvi18g02600\_t001 |  | Vitvi07g01457\_t001 |  |  |  |  |  |  |  |  |  |  |
| 1 | Vitvi18g02601\_t001 |  | \| \| |  |  |  |  |  |  |  |  |  |  |
| 1 | Vitvi18g00501\_t001 |  | \| \| |  |  |  |  |  |  |  |  |  |  |
| 1 | Vitvi18g04104\_t001 |  | \| \| |  |  |  |  |  |  |  |  |  |  |
| 1 | Vitvi18g04105\_t001 |  | \| \| |  |  |  |  |  |  |  |  |  |  |
| 1 | Vitvi18g04106\_t001 |  | \| \| |  |  |  |  |  |  |  |  |  |  |
| 1 | Vitvi18g00504\_t001 |  | \| \| |  |  |  |  |  |  |  |  |  |  |
| 1 | Vitvi18g00505\_t002 |  | Vitvi07g01451\_t001 |  |  |  |  |  |  |  |  |  |  |
| 1 | Vitvi18g00506\_t001 |  | \| \| |  |  |  |  |  |  |  |  |  |  |
| 1 | Vitvi18g04107\_t001 |  | \| \| |  |  |  |  |  |  |  |  |  |  |
| 1 | Vitvi18g00507\_t001 |  | Vitvi07g04599\_t001 |  |  |  |  |  |  |  |  |  |  |
| 1 | Vitvi18g00508\_t001 |  | Vitvi07g04600\_t001 |  |  |  |  |  |  |  |  |  |  |
| 1 | Vitvi18g04108\_t001 |  | \| \| |  |  |  |  |  |  |  |  |  |  |
| 1 | Vitvi18g04109\_t001 |  | \| \| |  |  |  |  |  |  |  |  |  |  |
| 1 | Vitvi18g00509\_t001 |  | \| \| |  |  |  |  |  |  |  |  |  |  |
| 1 | Vitvi18g00510\_t001 |  | \| \| |  |  |  |  |  |  |  |  |  |  |
| 1 | Vitvi18g02603\_t001 |  | \| \| |  |  |  |  |  |  |  |  |  |  |
| 1 | Vitvi18g04110\_t001 |  | \| \| |  |  |  |  |  |  |  |  |  |  |
| 1 | Vitvi18g00511\_t001 |  | Vitvi07g01449\_t001 |  |  |  |  |  |  |  |  |  |  |
| 1 | Vitvi18g04111\_t001 |  | \| \| |  |  |  |  |  |  |  |  |  |  |
| 1 | Vitvi18g02604\_t001 |  | \| \| |  |  |  |  |  |  |  |  |  |  |
| 1 | Vitvi18g02605\_t001 |  | \| \| |  |  |  |  |  |  |  |  |  |  |
| 1 | Vitvi18g04112\_t001 |  | \| \| |  |  |  |  |  |  |  |  |  |  |
| 1 | Vitvi18g02607\_t001 |  | Vitvi07g02577\_t001 |  |  |  |  |  |  |  |  |  |  |
| 1 | Vitvi18g00512\_t001 |  | \| \| |  |  |  |  |  |  |  |  |  |  |
| 1 | Vitvi18g00513\_t001 |  | Vitvi07g04603\_t001 |  |  |  |  |  |  |  |  |  |  |
| 1 | Vitvi18g00514\_t002 |  | \| \| |  |  |  |  |  |  |  |  |  |  |
| 1 | Vitvi18g04113\_t001 |  | \| \| |  |  |  |  |  |  |  |  |  |  |
| 1 | Vitvi18g00515\_t001 |  | \| \| |  |  |  |  |  |  |  |  |  |  |
| 1 | Vitvi18g00516\_t001 |  | Vitvi07g01444\_t001 |  |  |  |  |  |  |  |  |  |  |
| 1 | Vitvi18g04114\_t001 |  | Vitvi07g01441\_t001 |  |  |  |  |  |  |  |  |  |  |
| 1 | Vitvi18g02609\_t003 |  | \| \| |  |  |  |  |  |  |  |  |  |  |
| 1 | Vitvi18g00518\_t002 |  | Vitvi07g01437\_t001 |  |  |  |  |  |  |  |  |  |  |
| 1 | Vitvi18g02610\_t001.1.6037826d |  | Vitvi07g04607\_t001 |  |  |  |  |  |  |  |  |  |  |
| 1 | Vitvi18g04115\_t001 |  | \| \| |  |  |  |  |  |  |  |  |  |  |
| 1 | Vitvi18g02611\_t001 |  | \| \| |  |  |  |  |  |  |  |  |  |  |
| 1 | Vitvi18g04116\_t001 |  | \| \| |  |  |  |  |  |  |  |  |  |  |
| 1 | Vitvi18g00520\_t001 |  | \| \| |  |  |  |  |  |  |  |  |  |  |
| 1 | Vitvi18g00521\_t001 |  | \| \| |  |  |  |  |  |  |  |  |  |  |
| 1 | Vitvi18g00523\_t001 |  | \| \| |  |  |  |  |  |  |  |  |  |  |
| 1 | Vitvi18g00524\_t001 |  | Vitvi07g01431\_t001 |  |  |  |  |  |  |  |  |  |  |
| 1 | Vitvi18g00525\_t001 |  | Vitvi07g04608\_t001 |  |  |  |  |  |  |  |  |  |  |
| 1 | Vitvi18g02612\_t001 |  | \| \| |  |  |  |  |  |  |  |  |  |  |
| 1 | Vitvi18g00528\_t001 |  | \| \| |  |  |  |  |  |  |  |  |  |  |
| 1 | Vitvi18g00529\_t001 |  | \| \| |  |  |  |  |  |  |  |  |  |  |
| 1 | Vitvi18g00530\_t001 |  | \| \| |  |  |  |  |  |  |  |  |  |  |
| 1 | Vitvi18g04117\_t001 |  | \| \| |  |  |  |  |  |  |  |  |  |  |
| 1 | Vitvi18g02613\_t003 |  | \| \| |  |  |  |  |  |  |  |  |  |  |
| 1 | Vitvi18g00531\_t001 |  | \| \| |  |  |  |  |  |  |  |  |  |  |
| 1 | Vitvi18g00532\_t001 |  | \| \| |  |  |  |  |  |  |  |  |  |  |
| 1 | Vitvi18g00533\_t001 |  | Vitvi07g02572\_t001 |  |  |  |  |  |  |  |  |  |  |
| 1 | Vitvi18g00534\_t001 |  | \| \| |  |  |  |  |  |  |  |  |  |  |
| 1 | Vitvi18g00535\_t001 |  | \| \| |  |  |  |  |  |  |  |  |  |  |
| 1 | Vitvi18g02614\_t001 |  | \| \| |  |  |  |  |  |  |  |  |  |  |
| 1 | Vitvi18g00537\_t001 |  | \| \| |  |  |  |  |  |  |  |  |  |  |
| 1 | Vitvi18g00538\_t001 |  | \| \| |  |  |  |  |  |  |  |  |  |  |
| 1 | Vitvi18g00539\_t001 |  | \| \| |  |  |  |  |  |  |  |  |  |  |
| 1 | Vitvi18g00540\_t001 |  | \| \| |  |  |  |  |  |  |  |  |  |  |
| 1 | Vitvi18g00541\_t001 |  | \| \| |  |  |  |  |  |  |  |  |  |  |
| 1 | Vitvi18g00542\_t001 |  | \| \| |  |  |  |  |  |  |  |  |  |  |
| 1 | Vitvi18g00543\_t001 |  | \| \| |  |  |  |  |  |  |  |  |  |  |
| 1 | Vitvi18g02615\_t001 |  | \| \| |  |  |  |  |  |  |  |  |  |  |
| 1 | Vitvi18g00544\_t001 |  | Vitvi07g01402\_t001 |  |  |  |  |  |  |  |  |  |  |
| 1 | Vitvi18g00546\_t001 |  | \| \| |  |  |  |  |  |  |  |  |  |  |
| 1 | Vitvi18g04118\_t001 |  | \| \| |  |  |  |  |  |  |  |  |  |  |
| 1 | Vitvi18g04119\_t001 |  | \| \| |  |  |  |  |  |  |  |  |  |  |
| 1 | Vitvi18g02616\_t001 |  | \| \| |  |  |  |  |  |  |  |  |  |  |
| 1 | Vitvi18g00547\_t001 |  | \| \| |  |  |  |  |  |  |  |  |  |  |
| 1 | Vitvi18g00548\_t001 |  | \| \| |  |  |  |  |  |  |  |  |  |  |
| 1 | Vitvi18g00549\_t001 |  | Vitvi07g01388\_t001 |  |  |  |  |  |  |  |  |  |  |
| 1 | Vitvi18g00550\_t001 |  | \| \| |  |  |  |  |  |  |  |  |  |  |
| 1 | Vitvi18g00551\_t001 |  | \| \| |  |  |  |  |  |  |  |  |  |  |
| 1 | Vitvi18g00552\_t001 |  | \| \| |  |  |  |  |  |  |  |  |  |  |
| 2 | Vitvi18g00553\_t001 |  | Vitvi07g01520\_t001 |  | Vitvi03g00819\_t001 |  |  |  |  |  |  |  |  |
| 2 | Vitvi18g04120\_t001 |  | \| \| |  | \| \| |  |  |  |  |  |  |  |  |
| 2 | Vitvi18g00556\_t001 |  | \| \| |  | \| \| |  |  |  |  |  |  |  |  |
| 2 | Vitvi18g04121\_t001 |  | \| \| |  | \| \| |  |  |  |  |  |  |  |  |
| 2 | Vitvi18g00557\_t001 |  | \| \| |  | Vitvi03g00842\_t001 |  |  |  |  |  |  |  |  |
| 2 | Vitvi18g00558\_t001 |  | \| \| |  | \| \| |  |  |  |  |  |  |  |  |
| 2 | Vitvi18g02617\_t001 |  | \| \| |  | \| \| |  |  |  |  |  |  |  |  |
| 2 | Vitvi18g00560\_t002 |  | Vitvi07g02610\_t001 |  | \| \| |  |  |  |  |  |  |  |  |
| 2 | Vitvi18g02618\_t001 |  | \| \| |  | \| \| |  |  |  |  |  |  |  |  |
| 2 | Vitvi18g00561\_t001 |  | \| \| |  | \| \| |  |  |  |  |  |  |  |  |
| 2 | Vitvi18g00562\_t001 |  | Vitvi07g01523\_t001 |  | \| \| |  |  |  |  |  |  |  |  |
| 2 | Vitvi18g00563\_t001 |  | \| \| |  | Vitvi03g00849\_t001 |  |  |  |  |  |  |  |  |
| 2 | Vitvi18g00565\_t003 |  | \| \| |  | \| \| |  |  |  |  |  |  |  |  |
| 2 | Vitvi18g00566\_t001 |  | \| \| |  | Vitvi03g00852\_t001 |  |  |  |  |  |  |  |  |
| 2 | Vitvi18g00567\_t001 |  | Vitvi07g02613\_t001 |  | \| \| |  |  |  |  |  |  |  |  |
| 2 | Vitvi18g04122\_t001 |  | Vitvi07g01543\_t001 |  | \| \| |  |  |  |  |  |  |  |  |
| 2 | Vitvi18g00571\_t003 |  | Vitvi07g04642\_t003 |  | \| \| |  |  |  |  |  |  |  |  |
| 2 | Vitvi18g04123\_t001 |  | \| \| |  | \| \| |  |  |  |  |  |  |  |  |
| 2 | Vitvi18g04124\_t001 |  | \| \| |  | \| \| |  |  |  |  |  |  |  |  |
| 2 | Vitvi18g00572\_t001 |  | Vitvi07g04644\_t001 |  | \| \| |  |  |  |  |  |  |  |  |
| 2 | Vitvi18g04125\_t001 |  | \| \| |  | \| \| |  |  |  |  |  |  |  |  |
| 2 | Vitvi18g00573\_t001 |  | \| \| |  | Vitvi03g00862\_t001 |  |  |  |  |  |  |  |  |
| 2 | Vitvi18g00574\_t001 |  | \| \| |  | \| \| |  |  |  |  |  |  |  |  |
| 2 | Vitvi18g00576\_t001 |  | \| \| |  | \| \| |  |  |  |  |  |  |  |  |
| 2 | Vitvi18g00577\_t001 |  | \| \| |  | \| \| |  |  |  |  |  |  |  |  |
| 2 | Vitvi18g04126\_t001 |  | \| \| |  | \| \| |  |  |  |  |  |  |  |  |
| 2 | Vitvi18g04127\_t001 |  | \| \| |  | \| \| |  |  |  |  |  |  |  |  |
| 2 | Vitvi18g02620\_t001 |  | \| \| |  | \| \| |  |  |  |  |  |  |  |  |
| 2 | Vitvi18g04128\_t001 |  | \| \| |  | Vitvi03g04291\_t001 |  |  |  |  |  |  |  |  |
| 1 | Vitvi18g00580\_t001 |  | \| \| |  |  |  |  |  |  |  |  |  |  |
| 1 | Vitvi18g04129\_t001 |  | \| \| |  |  |  |  |  |  |  |  |  |  |
| 1 | Vitvi18g04130\_t001 |  | \| \| |  |  |  |  |  |  |  |  |  |  |
| 1 | Vitvi18g04131\_t001 |  | \| \| |  |  |  |  |  |  |  |  |  |  |
| 1 | Vitvi18g00583\_t001 |  | \| \| |  |  |  |  |  |  |  |  |  |  |
| 1 | Vitvi18g04132\_t001 |  | \| \| |  |  |  |  |  |  |  |  |  |  |
| 1 | Vitvi18g00587\_t001 |  | Vitvi07g04655\_t001 |  |  |  |  |  |  |  |  |  |  |
| 1 | Vitvi18g00588\_t001 |  | \| \| |  |  |  |  |  |  |  |  |  |  |
| 1 | Vitvi18g00589\_t001 |  | \| \| |  |  |  |  |  |  |  |  |  |  |
| 1 | Vitvi18g00590\_t001 |  | Vitvi07g04656\_t001 |  |  |  |  |  |  |  |  |  |  |
| 1 | Vitvi18g00591\_t001 |  | \| \| |  |  |  |  |  |  |  |  |  |  |
| 1 | Vitvi18g00593\_t001 |  | \| \| |  |  |  |  |  |  |  |  |  |  |
| 1 | Vitvi18g00594\_t002 |  | \| \| |  |  |  |  |  |  |  |  |  |  |
| 1 | Vitvi18g00595\_t006 |  | \| \| |  |  |  |  |  |  |  |  |  |  |
| 1 | Vitvi18g00596\_t001 |  | \| \| |  |  |  |  |  |  |  |  |  |  |
| 1 | Vitvi18g00597\_t002 |  | \| \| |  |  |  |  |  |  |  |  |  |  |
| 1 | Vitvi18g00598\_t001 |  | \| \| |  |  |  |  |  |  |  |  |  |  |
| 1 | Vitvi18g04133\_t001 |  | \| \| |  |  |  |  |  |  |  |  |  |  |
| 1 | Vitvi18g00599\_t001 |  | \| \| |  |  |  |  |  |  |  |  |  |  |
| 1 | Vitvi18g02621\_t001 |  | Vitvi07g03063\_t002 |  |  |  |  |  |  |  |  |  |  |
| 1 | Vitvi18g02622\_t001 |  | Vitvi07g03061\_t001 |  |  |  |  |  |  |  |  |  |  |
| 1 | Vitvi18g00600\_t001 |  | \| \| |  |  |  |  |  |  |  |  |  |  |
| 1 | Vitvi18g04134\_t001 |  | \| \| |  |  |  |  |  |  |  |  |  |  |
| 1 | Vitvi18g00601\_t001 |  | \| \| |  |  |  |  |  |  |  |  |  |  |
| 1 | Vitvi18g00602\_t001 |  | \| \| |  |  |  |  |  |  |  |  |  |  |
| 1 | Vitvi18g00603\_t001 |  | Vitvi07g03056\_t002 |  |  |  |  |  |  |  |  |  |  |
| 1 | Vitvi18g00604\_t001 |  | \| \| |  |  |  |  |  |  |  |  |  |  |
| 1 | Vitvi18g02625\_t001 |  | \| \| |  |  |  |  |  |  |  |  |  |  |
| 1 | Vitvi18g04135\_t001 |  | \| \| |  |  |  |  |  |  |  |  |  |  |
| 1 | Vitvi18g04136\_t001 |  | \| \| |  |  |  |  |  |  |  |  |  |  |
| 1 | Vitvi18g02627\_t001 |  | \| \| |  |  |  |  |  |  |  |  |  |  |
| 1 | Vitvi18g02628\_t001 |  | \| \| |  |  |  |  |  |  |  |  |  |  |
| 1 | Vitvi18g00605\_t001 |  | Vitvi07g03055\_t001 |  |  |  |  |  |  |  |  |  |  |
| 0 | Vitvi18g02629\_t001 |  |  |  |  |  |  |  |  |  |  |  |  |
| 0 | Vitvi18g00606\_t001 |  |  |  |  |  |  |  |  |  |  |  |  |
| 0 | Vitvi18g04137\_t001 |  |  |  |  |  |  |  |  |  |  |  |  |
| 0 | Vitvi18g00607\_t001 |  |  |  |  |  |  |  |  |  |  |  |  |
| 0 | Vitvi18g00608\_t001 |  |  |  |  |  |  |  |  |  |  |  |  |
| 0 | Vitvi18g00609\_t001 |  |  |  |  |  |  |  |  |  |  |  |  |
| 1 | Vitvi18g00610\_t001 |  | Vitvi15g04535\_t001 |  |  |  |  |  |  |  |  |  |  |
| 1 | Vitvi18g00611\_t002 |  | \| \| |  |  |  |  |  |  |  |  |  |  |
| 1 | Vitvi18g00612\_t001 |  | Vitvi15g04526\_t001 |  |  |  |  |  |  |  |  |  |  |
| 1 | Vitvi18g00614\_t001 |  | \| \| |  |  |  |  |  |  |  |  |  |  |
| 1 | Vitvi18g00615\_t001 |  | \| \| |  |  |  |  |  |  |  |  |  |  |
| 1 | Vitvi18g04138\_t001 |  | \| \| |  |  |  |  |  |  |  |  |  |  |
| 1 | Vitvi18g00616\_t001 |  | \| \| |  |  |  |  |  |  |  |  |  |  |
| 1 | Vitvi18g00617\_t001 |  | Vitvi15g04522\_t001 |  |  |  |  |  |  |  |  |  |  |
| 1 | Vitvi18g00618\_t001 |  | Vitvi15g04521\_t001 |  |  |  |  |  |  |  |  |  |  |
| 1 | Vitvi18g00619\_t001 |  | \| \| |  |  |  |  |  |  |  |  |  |  |
| 1 | Vitvi18g00621\_t001 |  | \| \| |  |  |  |  |  |  |  |  |  |  |
| 1 | Vitvi18g00622\_t001 |  | \| \| |  |  |  |  |  |  |  |  |  |  |
| 1 | Vitvi18g00623\_t001 |  | Vitvi15g04517\_t001 |  |  |  |  |  |  |  |  |  |  |
| 1 | Vitvi18g02631\_t001 |  | Vitvi15g04515\_t001 |  |  |  |  |  |  |  |  |  |  |
| 1 | Vitvi18g00624\_t001 |  | \| \| |  |  |  |  |  |  |  |  |  |  |
| 1 | Vitvi18g02632\_t001 |  | \| \| |  |  |  |  |  |  |  |  |  |  |
| 1 | Vitvi18g00626\_t001 |  | \| \| |  |  |  |  |  |  |  |  |  |  |
| 1 | Vitvi18g00628\_t001 |  | Vitvi15g04514\_t001 |  |  |  |  |  |  |  |  |  |  |
| 1 | Vitvi18g00631\_t001 |  | \| \| |  |  |  |  |  |  |  |  |  |  |
| 1 | Vitvi18g00632\_t001 |  | \| \| |  |  |  |  |  |  |  |  |  |  |
| 1 | Vitvi18g04139\_t001 |  | \| \| |  |  |  |  |  |  |  |  |  |  |
| 1 | Vitvi18g00634\_t001 |  | Vitvi15g04512\_t001 |  |  |  |  |  |  |  |  |  |  |
| 1 | Vitvi18g00635\_t001 |  | Vitvi15g04511\_t001 |  |  |  |  |  |  |  |  |  |  |
| 1 | Vitvi18g04140\_t001 |  | \| \| |  |  |  |  |  |  |  |  |  |  |
| 1 | Vitvi18g00636\_t001 |  | Vitvi15g04508\_t001 |  |  |  |  |  |  |  |  |  |  |
| 1 | Vitvi18g00637\_t001 |  | Vitvi15g04506\_t001 |  |  |  |  |  |  |  |  |  |  |
| 1 | Vitvi18g02635\_t001 |  | Vitvi15g04504\_t001 |  |  |  |  |  |  |  |  |  |  |
| 1 | Vitvi18g00638\_t001 |  | Vitvi15g04503\_t001 |  |  |  |  |  |  |  |  |  |  |
| 1 | Vitvi18g04141\_t001 |  | \| \| |  |  |  |  |  |  |  |  |  |  |
| 1 | Vitvi18g00639\_t001 |  | \| \| |  |  |  |  |  |  |  |  |  |  |
| 1 | Vitvi18g00640\_t001 |  | \| \| |  |  |  |  |  |  |  |  |  |  |
| 1 | Vitvi18g04142\_t001 |  | \| \| |  |  |  |  |  |  |  |  |  |  |
| 1 | Vitvi18g02636\_t001 |  | Vitvi15g04501\_t001 |  |  |  |  |  |  |  |  |  |  |
| 1 | Vitvi18g02637\_t001 |  | \| \| |  |  |  |  |  |  |  |  |  |  |
| 1 | Vitvi18g00641\_t001 |  | \| \| |  |  |  |  |  |  |  |  |  |  |
| 2 | Vitvi18g00642\_t001 |  | \| \| |  | Vitvi03g00691\_t001 |  |  |  |  |  |  |  |  |
| 2 | Vitvi18g00643\_t001 |  | \| \| |  | \| \| |  |  |  |  |  |  |  |  |
| 2 | Vitvi18g00644\_t001 |  | \| \| |  | \| \| |  |  |  |  |  |  |  |  |
| 2 | Vitvi18g02638\_t001 |  | \| \| |  | Vitvi03g00677\_t001 |  |  |  |  |  |  |  |  |
| 2 | Vitvi18g02639\_t001 |  | \| \| |  | \| \| |  |  |  |  |  |  |  |  |
| 2 | Vitvi18g00645\_t003 |  | Vitvi15g04494\_t001 |  | \| \| |  |  |  |  |  |  |  |  |
| 1 | Vitvi18g04143\_t001 |  |  |  | \| \| |  |  |  |  |  |  |  |  |
| 1 | Vitvi18g02643\_t001 |  |  |  | \| \| |  |  |  |  |  |  |  |  |
| 1 | Vitvi18g02644\_t001 |  |  |  | Vitvi03g00673\_t001 |  |  |  |  |  |  |  |  |
| 1 | Vitvi18g02646\_t001 |  |  |  | \| \| |  |  |  |  |  |  |  |  |
| 1 | Vitvi18g00646\_t001 |  |  |  | \| \| |  |  |  |  |  |  |  |  |
| 1 | Vitvi18g02647\_t001 |  |  |  | \| \| |  |  |  |  |  |  |  |  |
| 1 | Vitvi18g04144\_t001 |  |  |  | \| \| |  |  |  |  |  |  |  |  |
| 1 | Vitvi18g00647\_t001 |  |  |  | \| \| |  |  |  |  |  |  |  |  |
| 1 | Vitvi18g04145\_t001 |  |  |  | \| \| |  |  |  |  |  |  |  |  |
| 1 | Vitvi18g02648\_t001 |  |  |  | \| \| |  |  |  |  |  |  |  |  |
| 1 | Vitvi18g04146\_t001 |  |  |  | \| \| |  |  |  |  |  |  |  |  |
| 1 | Vitvi18g00651\_t001 |  |  |  | \| \| |  |  |  |  |  |  |  |  |
| 1 | Vitvi18g04147\_t001 |  |  |  | \| \| |  |  |  |  |  |  |  |  |
| 1 | Vitvi18g00652\_t001 |  |  |  | \| \| |  |  |  |  |  |  |  |  |
| 1 | Vitvi18g00653\_t001 |  |  |  | \| \| |  |  |  |  |  |  |  |  |
| 1 | Vitvi18g00654\_t001 |  |  |  | Vitvi03g00666\_t001 |  |  |  |  |  |  |  |  |
| 1 | Vitvi18g00655\_t001 |  |  |  | \| \| |  |  |  |  |  |  |  |  |
| 1 | Vitvi18g04148\_t001 |  |  |  | \| \| |  |  |  |  |  |  |  |  |
| 1 | Vitvi18g00656\_t001 |  |  |  | \| \| |  |  |  |  |  |  |  |  |
| 1 | Vitvi18g00657\_t001 |  |  |  | \| \| |  |  |  |  |  |  |  |  |
| 1 | Vitvi18g00658\_t001 |  |  |  | \| \| |  |  |  |  |  |  |  |  |
| 1 | Vitvi18g04149\_t001 |  |  |  | \| \| |  |  |  |  |  |  |  |  |
| 1 | Vitvi18g00659\_t001 |  |  |  | \| \| |  |  |  |  |  |  |  |  |
| 1 | Vitvi18g04150\_t001 |  |  |  | \| \| |  |  |  |  |  |  |  |  |
| 1 | Vitvi18g00660\_t001 |  |  |  | \| \| |  |  |  |  |  |  |  |  |
| 1 | Vitvi18g04151\_t001 |  |  |  | \| \| |  |  |  |  |  |  |  |  |
| 1 | Vitvi18g00661\_t001 |  |  |  | \| \| |  |  |  |  |  |  |  |  |
| 2 | Vitvi18g00664\_t001 |  | Vitvi07g01593\_t001 |  | \| \| |  |  |  |  |  |  |  |  |
| 2 | Vitvi18g00666\_t001 |  | \| \| |  | \| \| |  |  |  |  |  |  |  |  |
| 2 | Vitvi18g00667\_t001 |  | \| \| |  | \| \| |  |  |  |  |  |  |  |  |
| 2 | Vitvi18g00668\_t003 |  | Vitvi07g01595\_t001 |  | \| \| |  |  |  |  |  |  |  |  |
| 2 | Vitvi18g02649\_t001 |  | Vitvi07g04675\_t001 |  | \| \| |  |  |  |  |  |  |  |  |
| 2 | Vitvi18g00670\_t001 |  | \| \| |  | Vitvi03g00639\_t001 |  |  |  |  |  |  |  |  |
| 2 | Vitvi18g00671\_t001 |  | \| \| |  | \| \| |  |  |  |  |  |  |  |  |
| 2 | Vitvi18g00672\_t001 |  | \| \| |  | \| \| |  |  |  |  |  |  |  |  |
| 2 | Vitvi18g00673\_t001 |  | \| \| |  | Vitvi03g00635\_t001 |  |  |  |  |  |  |  |  |
| 2 | Vitvi18g00674\_t001 |  | \| \| |  | \| \| |  |  |  |  |  |  |  |  |
| 2 | Vitvi18g00675\_t001 |  | Vitvi07g01602\_t001 |  | Vitvi03g00630\_t001 |  |  |  |  |  |  |  |  |
| 2 | Vitvi18g04152\_t001 |  | \| \| |  | \| \| |  |  |  |  |  |  |  |  |
| 2 | Vitvi18g00677\_t001 |  | Vitvi07g01610\_t001 |  | Vitvi03g00628\_t001 |  |  |  |  |  |  |  |  |
| 2 | Vitvi18g00678\_t001 |  | \| \| |  | Vitvi03g00626\_t001 |  |  |  |  |  |  |  |  |
| 2 | Vitvi18g02651\_t001 |  | \| \| |  | Vitvi03g01606\_t001 |  |  |  |  |  |  |  |  |
| 2 | Vitvi18g04153\_t002 |  | \| \| |  | \| \| |  |  |  |  |  |  |  |  |
| 2 | Vitvi18g00679\_t001 |  | \| \| |  | Vitvi03g01605\_t002 |  |  |  |  |  |  |  |  |
| 2 | Vitvi18g00680\_t001 |  | \| \| |  | Vitvi03g00615\_t001 |  |  |  |  |  |  |  |  |
| 2 | Vitvi18g04154\_t001 |  | \| \| |  | \| \| |  |  |  |  |  |  |  |  |
| 2 | Vitvi18g00681\_t001 |  | Vitvi07g01620\_t002 |  | \| \| |  |  |  |  |  |  |  |  |
| 2 | Vitvi18g00682\_t001 |  | Vitvi07g01621\_t001 |  | \| \| |  |  |  |  |  |  |  |  |
| 2 | Vitvi18g04155\_t001 |  | \| \| |  | \| \| |  |  |  |  |  |  |  |  |
| 2 | Vitvi18g04156\_t001 |  | \| \| |  | \| \| |  |  |  |  |  |  |  |  |
| 2 | Vitvi18g04157\_t001 |  | Vitvi07g01622\_t001 |  | \| \| |  |  |  |  |  |  |  |  |
| 2 | Vitvi18g02654\_t001 |  | \| \| |  | \| \| |  |  |  |  |  |  |  |  |
| 2 | Vitvi18g00683\_t001 |  | Vitvi07g01627\_t001 |  | \| \| |  |  |  |  |  |  |  |  |
| 2 | Vitvi18g00684\_t001 |  | \| \| |  | \| \| |  |  |  |  |  |  |  |  |
| 2 | Vitvi18g00685\_t001 |  | \| \| |  | \| \| |  |  |  |  |  |  |  |  |
| 2 | Vitvi18g00686\_t001 |  | Vitvi07g01631\_t001 |  | Vitvi03g00605\_t001 |  |  |  |  |  |  |  |  |
| 2 | Vitvi18g00687\_t001 |  | \| \| |  | \| \| |  |  |  |  |  |  |  |  |
| 2 | Vitvi18g02655\_t001 |  | \| \| |  | \| \| |  |  |  |  |  |  |  |  |
| 2 | Vitvi18g00688\_t001 |  | \| \| |  | Vitvi03g00601\_t001 |  |  |  |  |  |  |  |  |
| 2 | Vitvi18g00689\_t001 |  | Vitvi07g01635\_t001 |  | \| \| |  |  |  |  |  |  |  |  |
| 2 | Vitvi18g00690\_t001 |  | \| \| |  | Vitvi03g00597\_t001 |  |  |  |  |  |  |  |  |
| 2 | Vitvi18g00691\_t001 |  | Vitvi07g01638\_t001 |  | Vitvi03g00593\_t001 |  |  |  |  |  |  |  |  |
| 2 | Vitvi18g00693\_t001 |  | Vitvi07g04684\_t001 |  | \| \| |  |  |  |  |  |  |  |  |
| 2 | Vitvi18g00694\_t001 |  | \| \| |  | \| \| |  |  |  |  |  |  |  |  |
| 2 | Vitvi18g00695\_t001 |  | Vitvi07g01642\_t001 |  | \| \| |  |  |  |  |  |  |  |  |
| 2 | Vitvi18g02657\_t001 |  | \| \| |  | \| \| |  |  |  |  |  |  |  |  |
| 2 | Vitvi18g00697\_t001 |  | \| \| |  | \| \| |  |  |  |  |  |  |  |  |
| 2 | Vitvi18g00699\_t001 |  | Vitvi07g04685\_t001 |  | \| \| |  |  |  |  |  |  |  |  |
| 2 | Vitvi18g04158\_t001 |  | \| \| |  | \| \| |  |  |  |  |  |  |  |  |
| 2 | Vitvi18g00700\_t001 |  | Vitvi07g04686\_t001 |  | \| \| |  |  |  |  |  |  |  |  |
| 2 | Vitvi18g04159\_t001 |  | \| \| |  | \| \| |  |  |  |  |  |  |  |  |
| 2 | Vitvi18g04160\_t001 |  | \| \| |  | \| \| |  |  |  |  |  |  |  |  |
| 2 | Vitvi18g00703\_t001 |  | \| \| |  | \| \| |  |  |  |  |  |  |  |  |
| 2 | Vitvi18g00704\_t001 |  | \| \| |  | \| \| |  |  |  |  |  |  |  |  |
| 2 | Vitvi18g00706\_t001 |  | \| \| |  | \| \| |  |  |  |  |  |  |  |  |
| 2 | Vitvi18g00707\_t001 |  | \| \| |  | Vitvi03g00584\_t001 |  |  |  |  |  |  |  |  |
| 2 | Vitvi18g00708\_t001 |  | Vitvi07g01645\_t001 |  | Vitvi03g00582\_t001 |  |  |  |  |  |  |  |  |
| 1 | Vitvi18g00711\_t001 |  | Vitvi07g01647\_t003 |  |  |  |  |  |  |  |  |  |  |
| 1 | Vitvi18g04161\_t001 |  | \| \| |  |  |  |  |  |  |  |  |  |  |
| 1 | Vitvi18g04162\_t001 |  | \| \| |  |  |  |  |  |  |  |  |  |  |
| 1 | Vitvi18g00716\_t001 |  | Vitvi07g01657\_t001 |  |  |  |  |  |  |  |  |  |  |
| 1 | Vitvi18g02659\_t001 |  | Vitvi07g01662\_t001 |  |  |  |  |  |  |  |  |  |  |
| 1 | Vitvi18g02660\_t001 |  | \| \| |  |  |  |  |  |  |  |  |  |  |
| 1 | Vitvi18g00717\_t001 |  | \| \| |  |  |  |  |  |  |  |  |  |  |
| 1 | Vitvi18g00718\_t001 |  | \| \| |  |  |  |  |  |  |  |  |  |  |
| 1 | Vitvi18g02661\_t001 |  | \| \| |  |  |  |  |  |  |  |  |  |  |
| 1 | Vitvi18g04163\_t001 |  | \| \| |  |  |  |  |  |  |  |  |  |  |
| 1 | Vitvi18g00719\_t001 |  | \| \| |  |  |  |  |  |  |  |  |  |  |
| 1 | Vitvi18g00720\_t001 |  | Vitvi07g01671\_t001 |  |  |  |  |  |  |  |  |  |  |
| 1 | Vitvi18g02662\_t001 |  | \| \| |  |  |  |  |  |  |  |  |  |  |
| 1 | Vitvi18g04164\_t001 |  | \| \| |  |  |  |  |  |  |  |  |  |  |
| 1 | Vitvi18g00721\_t002 |  | Vitvi07g01672\_t001 |  |  |  |  |  |  |  |  |  |  |
| 1 | Vitvi18g02663\_t001 |  | \| \| |  |  |  |  |  |  |  |  |  |  |
| 1 | Vitvi18g02664\_t001 |  | \| \| |  |  |  |  |  |  |  |  |  |  |
| 1 | Vitvi18g02665\_t001 |  | \| \| |  |  |  |  |  |  |  |  |  |  |
| 1 | Vitvi18g04165\_t001 |  | \| \| |  |  |  |  |  |  |  |  |  |  |
| 1 | Vitvi18g04166\_t001 |  | \| \| |  |  |  |  |  |  |  |  |  |  |
| 1 | Vitvi18g02666\_t001 |  | \| \| |  |  |  |  |  |  |  |  |  |  |
| 1 | Vitvi18g04167\_t001 |  | \| \| |  |  |  |  |  |  |  |  |  |  |
| 1 | Vitvi18g02667\_t001 |  | \| \| |  |  |  |  |  |  |  |  |  |  |
| 1 | Vitvi18g02669\_t001 |  | \| \| |  |  |  |  |  |  |  |  |  |  |
| 1 | Vitvi18g02670\_t001 |  | \| \| |  |  |  |  |  |  |  |  |  |  |
| 1 | Vitvi18g00722\_t001 |  | \| \| |  |  |  |  |  |  |  |  |  |  |
| 1 | Vitvi18g02671\_t001 |  | \| \| |  |  |  |  |  |  |  |  |  |  |
| 1 | Vitvi18g04168\_t001 |  | \| \| |  |  |  |  |  |  |  |  |  |  |
| 1 | Vitvi18g02672\_t001 |  | \| \| |  |  |  |  |  |  |  |  |  |  |
| 2 | Vitvi18g00725\_t001 |  | Vitvi07g01676\_t001 |  | Vitvi03g00559\_t001 |  |  |  |  |  |  |  |  |
| 2 | Vitvi18g04169\_t001 |  | \| \| |  | \| \| |  |  |  |  |  |  |  |  |
| 2 | Vitvi18g00727\_t002 |  | \| \| |  | Vitvi03g00552\_t001 |  |  |  |  |  |  |  |  |
| 2 | Vitvi18g00728\_t001 |  | \| \| |  | \| \| |  |  |  |  |  |  |  |  |
| 2 | Vitvi18g00729\_t001 |  | \| \| |  | Vitvi03g00551\_t001 |  |  |  |  |  |  |  |  |
| 2 | Vitvi18g02673\_t001 |  | \| \| |  | \| \| |  |  |  |  |  |  |  |  |
| 2 | Vitvi18g00730\_t001 |  | \| \| |  | \| \| |  |  |  |  |  |  |  |  |
| 2 | Vitvi18g00731\_t001 |  | Vitvi07g01683\_t001 |  | Vitvi03g00542\_t001 |  |  |  |  |  |  |  |  |
| 2 | Vitvi18g00732\_t001 |  | \| \| |  | \| \| |  |  |  |  |  |  |  |  |
| 2 | Vitvi18g00734\_t001 |  | Vitvi07g01684\_t001 |  | \| \| |  |  |  |  |  |  |  |  |
| 2 | Vitvi18g04170\_t001 |  | \| \| |  | \| \| |  |  |  |  |  |  |  |  |
| 2 | Vitvi18g00736\_t001 |  | \| \| |  | \| \| |  |  |  |  |  |  |  |  |
| 2 | Vitvi18g00737\_t001 |  | \| \| |  | Vitvi03g00541\_t001 |  |  |  |  |  |  |  |  |
| 2 | Vitvi18g00739\_t001 |  | \| \| |  | \| \| |  |  |  |  |  |  |  |  |
| 2 | Vitvi18g00740\_t002 |  | Vitvi07g01690\_t001 |  | Vitvi03g00539\_t001 |  |  |  |  |  |  |  |  |
| 2 | Vitvi18g00742\_t001 |  | Vitvi07g01694\_t001 |  | \| \| |  |  |  |  |  |  |  |  |
| 2 | Vitvi18g00743\_t001 |  | \| \| |  | Vitvi03g00536\_t001 |  |  |  |  |  |  |  |  |
| 2 | Vitvi18g04171\_t001 |  | \| \| |  | \| \| |  |  |  |  |  |  |  |  |
| 2 | Vitvi18g04172\_t001 |  | \| \| |  | \| \| |  |  |  |  |  |  |  |  |
| 2 | Vitvi18g00747\_t001 |  | Vitvi07g01698\_t001 |  | \| \| |  |  |  |  |  |  |  |  |
| 2 | Vitvi18g00748\_t001 |  | \| \| |  | \| \| |  |  |  |  |  |  |  |  |
| 2 | Vitvi18g04173\_t001 |  | \| \| |  | \| \| |  |  |  |  |  |  |  |  |
| 2 | Vitvi18g00749\_t002 |  | Vitvi07g01699\_t002 |  | \| \| |  |  |  |  |  |  |  |  |
| 2 | Vitvi18g00752\_t001 |  | Vitvi07g01700\_t001 |  | \| \| |  |  |  |  |  |  |  |  |
| 2 | Vitvi18g00753\_t001 |  | \| \| |  | Vitvi03g00533\_t001 |  |  |  |  |  |  |  |  |
| 2 | Vitvi18g00754\_t001 |  | \| \| |  | \| \| |  |  |  |  |  |  |  |  |
| 2 | Vitvi18g00755\_t001 |  | \| \| |  | \| \| |  |  |  |  |  |  |  |  |
| 2 | Vitvi18g04174\_t001 |  | Vitvi07g01702\_t001 |  | \| \| |  |  |  |  |  |  |  |  |
| 2 | Vitvi18g00757\_t001 |  | \| \| |  | \| \| |  |  |  |  |  |  |  |  |
| 2 | Vitvi18g00758\_t001 |  | Vitvi07g01705\_t001 |  | Vitvi03g00531\_t001 |  |  |  |  |  |  |  |  |
| 2 | Vitvi18g00759\_t001 |  | Vitvi07g04707\_t001 |  | \| \| |  |  |  |  |  |  |  |  |
| 2 | Vitvi18g02675\_t001 |  | \| \| |  | \| \| |  |  |  |  |  |  |  |  |
| 2 | Vitvi18g02676\_t001 |  | \| \| |  | \| \| |  |  |  |  |  |  |  |  |
| 2 | Vitvi18g04175\_t001 |  | \| \| |  | \| \| |  |  |  |  |  |  |  |  |
| 2 | Vitvi18g04176\_t001 |  | \| \| |  | \| \| |  |  |  |  |  |  |  |  |
| 2 | Vitvi18g04177\_t001 |  | Vitvi07g01714\_t001 |  | \| \| |  |  |  |  |  |  |  |  |
| 2 | Vitvi18g02678\_t001 |  | \| \| |  | \| \| |  |  |  |  |  |  |  |  |
| 2 | Vitvi18g04178\_t001 |  | \| \| |  | \| \| |  |  |  |  |  |  |  |  |
| 2 | Vitvi18g04179\_t001 |  | \| \| |  | \| \| |  |  |  |  |  |  |  |  |
| 2 | Vitvi18g04180\_t001 |  | \| \| |  | \| \| |  |  |  |  |  |  |  |  |
| 2 | Vitvi18g04181\_t001 |  | \| \| |  | \| \| |  |  |  |  |  |  |  |  |
| 2 | Vitvi18g04182\_t001 |  | \| \| |  | \| \| |  |  |  |  |  |  |  |  |
| 2 | Vitvi18g00761\_t001 |  | \| \| |  | \| \| |  |  |  |  |  |  |  |  |
| 2 | Vitvi18g04183\_t001 |  | \| \| |  | \| \| |  |  |  |  |  |  |  |  |
| 2 | Vitvi18g00762\_t001 |  | Vitvi07g01728\_t001 |  | Vitvi03g00521\_t001 |  |  |  |  |  |  |  |  |
| 2 | Vitvi18g00763\_t001 |  | Vitvi07g01729\_t001 |  | \| \| |  |  |  |  |  |  |  |  |
| 2 | Vitvi18g00764\_t001 |  | \| \| |  | \| \| |  |  |  |  |  |  |  |  |
| 2 | Vitvi18g00765\_t001 |  | \| \| |  | \| \| |  |  |  |  |  |  |  |  |
| 2 | Vitvi18g00766\_t001 |  | \| \| |  | Vitvi03g00520\_t001 |  |  |  |  |  |  |  |  |
| 2 | Vitvi18g00768\_t002 |  | Vitvi07g01732\_t001 |  | \| \| |  |  |  |  |  |  |  |  |
| 2 | Vitvi18g00769\_t001 |  | \| \| |  | \| \| |  |  |  |  |  |  |  |  |
| 2 | Vitvi18g00770\_t001 |  | Vitvi07g01734\_t001 |  | Vitvi03g01577\_t001 |  |  |  |  |  |  |  |  |
| 2 | Vitvi18g00772\_t001 |  | \| \| |  | \| \| |  |  |  |  |  |  |  |  |
| 2 | Vitvi18g00773\_t001 |  | \| \| |  | \| \| |  |  |  |  |  |  |  |  |
| 2 | Vitvi18g00775\_t001 |  | Vitvi07g01736\_t001 |  | \| \| |  |  |  |  |  |  |  |  |
| 2 | Vitvi18g00776\_t001 |  | \| \| |  | \| \| |  |  |  |  |  |  |  |  |
| 2 | Vitvi18g00777\_t001 |  | \| \| |  | \| \| |  |  |  |  |  |  |  |  |
| 2 | Vitvi18g00778\_t001 |  | \| \| |  | \| \| |  |  |  |  |  |  |  |  |
| 2 | Vitvi18g00779\_t001 |  | Vitvi07g01740\_t001 |  | \| \| |  |  |  |  |  |  |  |  |
| 2 | Vitvi18g04184\_t001 |  | \| \| |  | \| \| |  |  |  |  |  |  |  |  |
| 2 | Vitvi18g00780\_t001 |  | Vitvi07g02664\_t001 |  | \| \| |  |  |  |  |  |  |  |  |
| 2 | Vitvi18g00781\_t002 |  | Vitvi07g01744\_t001 |  | \| \| |  |  |  |  |  |  |  |  |
| 2 | Vitvi18g00782\_t001 |  | \| \| |  | \| \| |  |  |  |  |  |  |  |  |
| 2 | Vitvi18g00783\_t001 |  | \| \| |  | \| \| |  |  |  |  |  |  |  |  |
| 2 | Vitvi18g00784\_t001 |  | \| \| |  | Vitvi03g01574\_t003 |  |  |  |  |  |  |  |  |
| 2 | Vitvi18g04185\_t001 |  | \| \| |  | \| \| |  |  |  |  |  |  |  |  |
| 2 | Vitvi18g04186\_t001 |  | \| \| |  | \| \| |  |  |  |  |  |  |  |  |
| 2 | Vitvi18g00785\_t001 |  | Vitvi07g02666\_t001 |  | \| \| |  |  |  |  |  |  |  |  |
| 2 | Vitvi18g00787\_t001 |  | \| \| |  | \| \| |  |  |  |  |  |  |  |  |
| 2 | Vitvi18g00788\_t001 |  | \| \| |  | \| \| |  |  |  |  |  |  |  |  |
| 2 | Vitvi18g00789\_t001 |  | \| \| |  | Vitvi03g01573\_t001 |  |  |  |  |  |  |  |  |
| 2 | Vitvi18g00790\_t001 |  | \| \| |  | \| \| |  |  |  |  |  |  |  |  |
| 2 | Vitvi18g00792\_t001 |  | Vitvi07g01751\_t001 |  | Vitvi03g00508\_t001 |  |  |  |  |  |  |  |  |
| 2 | Vitvi18g04187\_t001 |  | \| \| |  | \| \| |  |  |  |  |  |  |  |  |
| 2 | Vitvi18g00793\_t001 |  | \| \| |  | \| \| |  |  |  |  |  |  |  |  |
| 2 | Vitvi18g00794\_t001 |  | \| \| |  | \| \| |  |  |  |  |  |  |  |  |
| 2 | Vitvi18g00795\_t001 |  | Vitvi07g01753\_t001 |  | \| \| |  |  |  |  |  |  |  |  |
| 2 | Vitvi18g00796\_t001 |  | \| \| |  | Vitvi03g00499\_t001 |  |  |  |  |  |  |  |  |
| 2 | Vitvi18g02680\_t001 |  | \| \| |  | \| \| |  |  |  |  |  |  |  |  |
| 2 | Vitvi18g00797\_t001 |  | \| \| |  | \| \| |  |  |  |  |  |  |  |  |
| 2 | Vitvi18g02681\_t001 |  | \| \| |  | \| \| |  |  |  |  |  |  |  |  |
| 2 | Vitvi18g00799\_t001 |  | \| \| |  | \| \| |  |  |  |  |  |  |  |  |
| 2 | Vitvi18g00801\_t001 |  | \| \| |  | \| \| |  |  |  |  |  |  |  |  |
| 2 | Vitvi18g00802\_t001 |  | \| \| |  | \| \| |  |  |  |  |  |  |  |  |
| 2 | Vitvi18g04188\_t001 |  | \| \| |  | \| \| |  |  |  |  |  |  |  |  |
| 2 | Vitvi18g02683\_t001 |  | \| \| |  | \| \| |  |  |  |  |  |  |  |  |
| 2 | Vitvi18g02684\_t001 |  | \| \| |  | \| \| |  |  |  |  |  |  |  |  |
| 2 | Vitvi18g02685\_t001 |  | \| \| |  | \| \| |  |  |  |  |  |  |  |  |
| 2 | Vitvi18g04189\_t001 |  | \| \| |  | \| \| |  |  |  |  |  |  |  |  |
| 2 | Vitvi18g04190\_t001 |  | \| \| |  | \| \| |  |  |  |  |  |  |  |  |
| 2 | Vitvi18g04191\_t001 |  | \| \| |  | \| \| |  |  |  |  |  |  |  |  |
| 2 | Vitvi18g02687\_t001 |  | \| \| |  | \| \| |  |  |  |  |  |  |  |  |
| 2 | Vitvi18g04192\_t001 |  | \| \| |  | \| \| |  |  |  |  |  |  |  |  |
| 2 | Vitvi18g04193\_t001 |  | \| \| |  | \| \| |  |  |  |  |  |  |  |  |
| 2 | Vitvi18g02689\_t001 |  | Vitvi07g01760\_t001 |  | \| \| |  |  |  |  |  |  |  |  |
| 2 | Vitvi18g00804\_t002 |  | \| \| |  | \| \| |  |  |  |  |  |  |  |  |
| 2 | Vitvi18g02690\_t001 |  | \| \| |  | \| \| |  |  |  |  |  |  |  |  |
| 2 | Vitvi18g00806\_t001 |  | \| \| |  | \| \| |  |  |  |  |  |  |  |  |
| 2 | Vitvi18g00807\_t001 |  | Vitvi07g01762\_t001 |  | Vitvi03g04176\_t001 |  |  |  |  |  |  |  |  |
| 2 | Vitvi18g00808\_t001 |  | \| \| |  | \| \| |  |  |  |  |  |  |  |  |
| 2 | Vitvi18g00809\_t001 |  | \| \| |  | Vitvi03g00486\_t001 |  |  |  |  |  |  |  |  |
| 2 | Vitvi18g00810\_t001 |  | \| \| |  | \| \| |  |  |  |  |  |  |  |  |
| 2 | Vitvi18g00811\_t001 |  | \| \| |  | \| \| |  |  |  |  |  |  |  |  |
| 2 | Vitvi18g04194\_t001 |  | \| \| |  | \| \| |  |  |  |  |  |  |  |  |
| 2 | Vitvi18g02691\_t001 |  | \| \| |  | \| \| |  |  |  |  |  |  |  |  |
| 2 | Vitvi18g00812\_t001 |  | \| \| |  | \| \| |  |  |  |  |  |  |  |  |
| 2 | Vitvi18g00813\_t001 |  | \| \| |  | \| \| |  |  |  |  |  |  |  |  |
| 2 | Vitvi18g00815\_t001 |  | \| \| |  | Vitvi03g00483\_t001 |  |  |  |  |  |  |  |  |
| 2 | Vitvi18g00816\_t001 |  | \| \| |  | \| \| |  |  |  |  |  |  |  |  |
| 2 | Vitvi18g00817\_t001 |  | \| \| |  | \| \| |  |  |  |  |  |  |  |  |
| 2 | Vitvi18g00818\_t001 |  | \| \| |  | \| \| |  |  |  |  |  |  |  |  |
| 2 | Vitvi18g02692\_t001 |  | Vitvi07g01766\_t001 |  | \| \| |  |  |  |  |  |  |  |  |
| 2 | Vitvi18g04195\_t001 |  | \| \| |  | \| \| |  |  |  |  |  |  |  |  |
| 2 | Vitvi18g02695\_t001 |  | \| \| |  | \| \| |  |  |  |  |  |  |  |  |
| 2 | Vitvi18g00821\_t001 |  | \| \| |  | \| \| |  |  |  |  |  |  |  |  |
| 2 | Vitvi18g04196\_t001 |  | \| \| |  | \| \| |  |  |  |  |  |  |  |  |
| 2 | Vitvi18g02696\_t001 |  | \| \| |  | \| \| |  |  |  |  |  |  |  |  |
| 2 | Vitvi18g04197\_t001 |  | \| \| |  | \| \| |  |  |  |  |  |  |  |  |
| 2 | Vitvi18g04198\_t001 |  | \| \| |  | \| \| |  |  |  |  |  |  |  |  |
| 2 | Vitvi18g00825\_t001 |  | \| \| |  | \| \| |  |  |  |  |  |  |  |  |
| 2 | Vitvi18g02698\_t001 |  | \| \| |  | \| \| |  |  |  |  |  |  |  |  |
| 2 | Vitvi18g04199\_t001 |  | \| \| |  | \| \| |  |  |  |  |  |  |  |  |
| 2 | Vitvi18g02699\_t001 |  | \| \| |  | \| \| |  |  |  |  |  |  |  |  |
| 2 | Vitvi18g02703\_t001 |  | Vitvi07g01769\_t001 |  | \| \| |  |  |  |  |  |  |  |  |
| 2 | Vitvi18g02704\_t001 |  | \| \| |  | \| \| |  |  |  |  |  |  |  |  |
| 2 | Vitvi18g00828\_t001 |  | \| \| |  | \| \| |  |  |  |  |  |  |  |  |
| 2 | Vitvi18g00830\_t001 |  | \| \| |  | \| \| |  |  |  |  |  |  |  |  |
| 2 | Vitvi18g00831\_t001 |  | Vitvi07g01773\_t001 |  | Vitvi03g00477\_t001 |  |  |  |  |  |  |  |  |
| 1 | Vitvi18g00832\_t001 |  |  |  | \| \| |  |  |  |  |  |  |  |  |
| 1 | Vitvi18g00833\_t001 |  |  |  | \| \| |  |  |  |  |  |  |  |  |
| 1 | Vitvi18g00834\_t001 |  |  |  | Vitvi03g00476\_t002 |  |  |  |  |  |  |  |  |
| 1 | Vitvi18g00835\_t002 |  |  |  | \| \| |  |  |  |  |  |  |  |  |
| 1 | Vitvi18g00836\_t001 |  |  |  | Vitvi03g00474\_t001 |  |  |  |  |  |  |  |  |
| 1 | Vitvi18g00837\_t001 |  |  |  | \| \| |  |  |  |  |  |  |  |  |
| 2 | Vitvi18g00838\_t001 |  | Vitvi07g04541\_t001 |  | \| \| |  |  |  |  |  |  |  |  |
| 2 | Vitvi18g00839\_t001 |  | \| \| |  | Vitvi03g00473\_t001 |  |  |  |  |  |  |  |  |
| 2 | Vitvi18g02706\_t001 |  | \| \| |  | \| \| |  |  |  |  |  |  |  |  |
| 2 | Vitvi18g00840\_t001 |  | \| \| |  | \| \| |  |  |  |  |  |  |  |  |
| 2 | Vitvi18g00842\_t001 |  | \| \| |  | \| \| |  |  |  |  |  |  |  |  |
| 2 | Vitvi18g04200\_t001 |  | \| \| |  | \| \| |  |  |  |  |  |  |  |  |
| 2 | Vitvi18g00843\_t001 |  | \| \| |  | \| \| |  |  |  |  |  |  |  |  |
| 2 | Vitvi18g02707\_t001 |  | \| \| |  | Vitvi03g00460\_t001 |  |  |  |  |  |  |  |  |
| 2 | Vitvi18g02708\_t001 |  | \| \| |  | \| \| |  |  |  |  |  |  |  |  |
| 2 | Vitvi18g02709\_t001 |  | \| \| |  | \| \| |  |  |  |  |  |  |  |  |
| 2 | Vitvi18g00844\_t002 |  | \| \| |  | \| \| |  |  |  |  |  |  |  |  |
| 2 | Vitvi18g00845\_t001 |  | \| \| |  | Vitvi03g04171\_t001 |  |  |  |  |  |  |  |  |
| 1 | Vitvi18g00846\_t001 |  | \| \| |  |  |  |  |  |  |  |  |  |  |
| 1 | Vitvi18g00847\_t002 |  | \| \| |  |  |  |  |  |  |  |  |  |  |
| 1 | Vitvi18g00848\_t001 |  | \| \| |  |  |  |  |  |  |  |  |  |  |
| 1 | Vitvi18g00849\_t001 |  | Vitvi07g04530\_t001 |  |  |  |  |  |  |  |  |  |  |
| 1 | Vitvi18g00850\_t001 |  | Vitvi07g01313\_t001 |  |  |  |  |  |  |  |  |  |  |
| 1 | Vitvi18g00851\_t001 |  | \| \| |  |  |  |  |  |  |  |  |  |  |
| 1 | Vitvi18g00852\_t001 |  | \| \| |  |  |  |  |  |  |  |  |  |  |
| 1 | Vitvi18g00855\_t001 |  | \| \| |  |  |  |  |  |  |  |  |  |  |
| 1 | Vitvi18g00856\_t001 |  | \| \| |  |  |  |  |  |  |  |  |  |  |
| 1 | Vitvi18g00857\_t001 |  | \| \| |  |  |  |  |  |  |  |  |  |  |
| 2 | Vitvi18g00858\_t001 |  | Vitvi07g01298\_t001 |  | Vitvi03g00430\_t001 |  |  |  |  |  |  |  |  |
| 2 | Vitvi18g04201\_t001 |  | \| \| |  | \| \| |  |  |  |  |  |  |  |  |
| 2 | Vitvi18g04202\_t001 |  | \| \| |  | \| \| |  |  |  |  |  |  |  |  |
| 2 | Vitvi18g04203\_t001 |  | \| \| |  | Vitvi03g00427\_t001 |  |  |  |  |  |  |  |  |
| 2 | Vitvi18g00863\_t001 |  | Vitvi07g01295\_t001 |  | \| \| |  |  |  |  |  |  |  |  |
| 2 | Vitvi18g00865\_t001 |  | \| \| |  | Vitvi03g00419\_t001 |  |  |  |  |  |  |  |  |
| 2 | Vitvi18g00866\_t001 |  | Vitvi07g01291\_t001 |  | \| \| |  |  |  |  |  |  |  |  |
| 2 | Vitvi18g00867\_t002 |  | \| \| |  | Vitvi03g04146\_t001 |  |  |  |  |  |  |  |  |
| 2 | Vitvi18g00868\_t001 |  | \| \| |  | \| \| |  |  |  |  |  |  |  |  |
| 2 | Vitvi18g00869\_t001 |  | \| \| |  | \| \| |  |  |  |  |  |  |  |  |
| 2 | Vitvi18g00870\_t001 |  | \| \| |  | Vitvi03g01524\_t001 |  |  |  |  |  |  |  |  |
| 2 | Vitvi18g02711\_t001 |  | \| \| |  | \| \| |  |  |  |  |  |  |  |  |
| 2 | Vitvi18g04204\_t001 |  | \| \| |  | \| \| |  |  |  |  |  |  |  |  |
| 2 | Vitvi18g02712\_t001 |  | \| \| |  | \| \| |  |  |  |  |  |  |  |  |
| 2 | Vitvi18g02714\_t001 |  | \| \| |  | \| \| |  |  |  |  |  |  |  |  |
| 2 | Vitvi18g04205\_t001 |  | \| \| |  | \| \| |  |  |  |  |  |  |  |  |
| 2 | Vitvi18g02715\_t001 |  | \| \| |  | \| \| |  |  |  |  |  |  |  |  |
| 2 | Vitvi18g04206\_t001 |  | \| \| |  | \| \| |  |  |  |  |  |  |  |  |
| 2 | Vitvi18g00873\_t001 |  | \| \| |  | \| \| |  |  |  |  |  |  |  |  |
| 2 | Vitvi18g04207\_t001 |  | \| \| |  | \| \| |  |  |  |  |  |  |  |  |
| 2 | Vitvi18g04208\_t001 |  | \| \| |  | \| \| |  |  |  |  |  |  |  |  |
| 2 | Vitvi18g04209\_t001 |  | \| \| |  | \| \| |  |  |  |  |  |  |  |  |
| 2 | Vitvi18g02720\_t001 |  | \| \| |  | \| \| |  |  |  |  |  |  |  |  |
| 2 | Vitvi18g02718\_t001 |  | Vitvi07g01288\_t001 |  | \| \| |  |  |  |  |  |  |  |  |
| 2 | Vitvi18g00876\_t001 |  | \| \| |  | \| \| |  |  |  |  |  |  |  |  |
| 2 | Vitvi18g00878\_t001 |  | \| \| |  | \| \| |  |  |  |  |  |  |  |  |
| 2 | Vitvi18g00879\_t001 |  | \| \| |  | \| \| |  |  |  |  |  |  |  |  |
| 2 | Vitvi18g04210\_t001 |  | \| \| |  | \| \| |  |  |  |  |  |  |  |  |
| 2 | Vitvi18g04211\_t001 |  | \| \| |  | \| \| |  |  |  |  |  |  |  |  |
| 2 | Vitvi18g04212\_t001 |  | \| \| |  | \| \| |  |  |  |  |  |  |  |  |
| 2 | Vitvi18g00881\_t001 |  | \| \| |  | \| \| |  |  |  |  |  |  |  |  |
| 2 | Vitvi18g00882\_t001 |  | Vitvi07g01286\_t001 |  | Vitvi03g00397\_t001 |  |  |  |  |  |  |  |  |
| 2 | Vitvi18g02724\_t001 |  | Vitvi07g04495\_t001 |  | \| \| |  |  |  |  |  |  |  |  |
| 2 | Vitvi18g00885\_t001 |  | Vitvi07g01280\_t001 |  | \| \| |  |  |  |  |  |  |  |  |
| 2 | Vitvi18g00886\_t001 |  | \| \| |  | Vitvi03g00391\_t001 |  |  |  |  |  |  |  |  |
| 2 | Vitvi18g00887\_t001 |  | \| \| |  | \| \| |  |  |  |  |  |  |  |  |
| 2 | Vitvi18g04213\_t001 |  | Vitvi07g01278\_t002 |  | \| \| |  |  |  |  |  |  |  |  |
| 2 | Vitvi18g04214\_t001 |  | \| \| |  | \| \| |  |  |  |  |  |  |  |  |
| 2 | Vitvi18g00888\_t001 |  | \| \| |  | \| \| |  |  |  |  |  |  |  |  |
| 2 | Vitvi18g00890\_t001 |  | \| \| |  | \| \| |  |  |  |  |  |  |  |  |
| 2 | Vitvi18g04215\_t001 |  | \| \| |  | \| \| |  |  |  |  |  |  |  |  |
| 2 | Vitvi18g02727\_t001 |  | \| \| |  | \| \| |  |  |  |  |  |  |  |  |
| 2 | Vitvi18g00892\_t002 |  | \| \| |  | \| \| |  |  |  |  |  |  |  |  |
| 2 | Vitvi18g00893\_t001 |  | \| \| |  | \| \| |  |  |  |  |  |  |  |  |
| 2 | Vitvi18g00894\_t001 |  | Vitvi07g01275\_t001 |  | \| \| |  |  |  |  |  |  |  |  |
| 2 | Vitvi18g00895\_t001 |  | \| \| |  | \| \| |  |  |  |  |  |  |  |  |
| 2 | Vitvi18g00896\_t001 |  | \| \| |  | \| \| |  |  |  |  |  |  |  |  |
| 2 | Vitvi18g02728\_t001 |  | \| \| |  | \| \| |  |  |  |  |  |  |  |  |
| 2 | Vitvi18g00897\_t001 |  | Vitvi07g01261\_t001 |  | Vitvi03g00377\_t001 |  |  |  |  |  |  |  |  |
| 2 | Vitvi18g04216\_t001 |  | \| \| |  | \| \| |  |  |  |  |  |  |  |  |
| 2 | Vitvi18g00898\_t001 |  | \| \| |  | \| \| |  |  |  |  |  |  |  |  |
| 2 | Vitvi18g00899\_t001 |  | \| \| |  | \| \| |  |  |  |  |  |  |  |  |
| 2 | Vitvi18g00900\_t001 |  | \| \| |  | \| \| |  |  |  |  |  |  |  |  |
| 2 | Vitvi18g00901\_t001 |  | Vitvi07g01242\_t001 |  | \| \| |  |  |  |  |  |  |  |  |
| 2 | Vitvi18g00902\_t001 |  | Vitvi04g01210\_t001 |  | \| \| |  |  |  |  |  |  |  |  |
| 2 | Vitvi18g00903\_t001 |  | Vitvi04g02091\_t001 |  | \| \| |  |  |  |  |  |  |  |  |
| 2 | Vitvi18g00904\_t001 |  | Vitvi04g01216\_t001 |  | \| \| |  |  |  |  |  |  |  |  |
| 2 | Vitvi18g00905\_t001 |  | Vitvi04g01220\_t001 |  | \| \| |  |  |  |  |  |  |  |  |
| 2 | Vitvi18g00908\_t001 |  | \| \| |  | \| \| |  |  |  |  |  |  |  |  |
| 2 | Vitvi18g00909\_t001 |  | Vitvi04g01222\_t001 |  | \| \| |  |  |  |  |  |  |  |  |
| 2 | Vitvi18g00910\_t001 |  | \| \| |  | \| \| |  |  |  |  |  |  |  |  |
| 2 | Vitvi18g04217\_t001 |  | \| \| |  | \| \| |  |  |  |  |  |  |  |  |
| 2 | Vitvi18g00912\_t001 |  | Vitvi04g01223\_t001 |  | Vitvi03g00372\_t001 |  |  |  |  |  |  |  |  |
| 2 | Vitvi18g00913\_t001 |  | Vitvi04g01224\_t001 |  | \| \| |  |  |  |  |  |  |  |  |
| 2 | Vitvi18g00915\_t001 |  | \| \| |  | Vitvi03g00370\_t003 |  |  |  |  |  |  |  |  |
| 2 | Vitvi18g00917\_t001 |  | \| \| |  | \| \| |  |  |  |  |  |  |  |  |
| 2 | Vitvi18g00918\_t001 |  | \| \| |  | \| \| |  |  |  |  |  |  |  |  |
| 2 | Vitvi18g02730\_t001 |  | Vitvi04g01225\_t001 |  | \| \| |  |  |  |  |  |  |  |  |
| 2 | Vitvi18g00919\_t001 |  | Vitvi04g01227\_t001 |  | \| \| |  |  |  |  |  |  |  |  |
| 2 | Vitvi18g02732\_t001 |  | \| \| |  | \| \| |  |  |  |  |  |  |  |  |
| 2 | Vitvi18g00920\_t001 |  | \| \| |  | \| \| |  |  |  |  |  |  |  |  |
| 2 | Vitvi18g00922\_t001 |  | Vitvi04g01230\_t001 |  | \| \| |  |  |  |  |  |  |  |  |
| 2 | Vitvi18g00923\_t001 |  | \| \| |  | Vitvi03g04128\_t001 |  |  |  |  |  |  |  |  |
| 2 | Vitvi18g00924\_t001 |  | Vitvi04g01234\_t001 |  | \| \| |  |  |  |  |  |  |  |  |
| 2 | Vitvi18g00925\_t001 |  | \| \| |  | \| \| |  |  |  |  |  |  |  |  |
| 2 | Vitvi18g04218\_t001 |  | Vitvi04g01237\_t001 |  | \| \| |  |  |  |  |  |  |  |  |
| 2 | Vitvi18g04219\_t001 |  | \| \| |  | \| \| |  |  |  |  |  |  |  |  |
| 2 | Vitvi18g02734\_t001 |  | \| \| |  | \| \| |  |  |  |  |  |  |  |  |
| 2 | Vitvi18g04220\_t001 |  | \| \| |  | \| \| |  |  |  |  |  |  |  |  |
| 2 | Vitvi18g00927\_t002 |  | \| \| |  | \| \| |  |  |  |  |  |  |  |  |
| 2 | Vitvi18g00928\_t002 |  | \| \| |  | Vitvi03g00364\_t001 |  |  |  |  |  |  |  |  |
| 2 | Vitvi18g00929\_t001 |  | \| \| |  | \| \| |  |  |  |  |  |  |  |  |
| 2 | Vitvi18g00930\_t003 |  | Vitvi04g02098\_t001 |  | Vitvi03g04127\_t001 |  |  |  |  |  |  |  |  |
| 2 | Vitvi18g00931\_t001 |  | \| \| |  | Vitvi03g04125\_t001 |  |  |  |  |  |  |  |  |
| 1 | Vitvi18g02736\_t001 |  | \| \| |  |  |  |  |  |  |  |  |  |  |
| 1 | Vitvi18g00933\_t005 |  | Vitvi04g01249\_t001 |  |  |  |  |  |  |  |  |  |  |
| 1 | Vitvi18g02738\_t001 |  | \| \| |  |  |  |  |  |  |  |  |  |  |
| 1 | Vitvi18g02739\_t001 |  | \| \| |  |  |  |  |  |  |  |  |  |  |
| 1 | Vitvi18g04221\_t001 |  | \| \| |  |  |  |  |  |  |  |  |  |  |
| 1 | Vitvi18g02741\_t001 |  | \| \| |  |  |  |  |  |  |  |  |  |  |
| 1 | Vitvi18g04222\_t001 |  | \| \| |  |  |  |  |  |  |  |  |  |  |
| 1 | Vitvi18g02742\_t001 |  | \| \| |  |  |  |  |  |  |  |  |  |  |
| 1 | Vitvi18g04223\_t001 |  | \| \| |  |  |  |  |  |  |  |  |  |  |
| 1 | Vitvi18g02743\_t001 |  | \| \| |  |  |  |  |  |  |  |  |  |  |
| 1 | Vitvi18g00935\_t001 |  | Vitvi04g01252\_t001 |  |  |  |  |  |  |  |  |  |  |
| 1 | Vitvi18g04224\_t001 |  | \| \| |  |  |  |  |  |  |  |  |  |  |
| 1 | Vitvi18g00936\_t001 |  | \| \| |  |  |  |  |  |  |  |  |  |  |
| 1 | Vitvi18g00937\_t001 |  | \| \| |  |  |  |  |  |  |  |  |  |  |
| 1 | Vitvi18g00938\_t001 |  | \| \| |  |  |  |  |  |  |  |  |  |  |
| 1 | Vitvi18g00940\_t001 |  | \| \| |  |  |  |  |  |  |  |  |  |  |
| 1 | Vitvi18g00941\_t001 |  | \| \| |  |  |  |  |  |  |  |  |  |  |
| 1 | Vitvi18g00942\_t001 |  | \| \| |  |  |  |  |  |  |  |  |  |  |
| 1 | Vitvi18g00943\_t001 |  | \| \| |  |  |  |  |  |  |  |  |  |  |
| 1 | Vitvi18g04225\_t001 |  | \| \| |  |  |  |  |  |  |  |  |  |  |
| 1 | Vitvi18g00944\_t001 |  | \| \| |  |  |  |  |  |  |  |  |  |  |
| 1 | Vitvi18g00946\_t001 |  | Vitvi04g01262\_t001 |  |  |  |  |  |  |  |  |  |  |
| 1 | Vitvi18g00947\_t001 |  | Vitvi04g01264\_t001 |  |  |  |  |  |  |  |  |  |  |
| 1 | Vitvi18g00948\_t001 |  | Vitvi04g01265\_t001 |  |  |  |  |  |  |  |  |  |  |
| 1 | Vitvi18g00949\_t001 |  | \| \| |  |  |  |  |  |  |  |  |  |  |
| 1 | Vitvi18g04226\_t001 |  | \| \| |  |  |  |  |  |  |  |  |  |  |
| 1 | Vitvi18g00953\_t001 |  | \| \| |  |  |  |  |  |  |  |  |  |  |
| 1 | Vitvi18g00954\_t001 |  | \| \| |  |  |  |  |  |  |  |  |  |  |
| 2 | Vitvi18g00955\_t001 |  | \| \| |  | Vitvi03g00340\_t001 |  |  |  |  |  |  |  |  |
| 2 | Vitvi18g00956\_t001 |  | \| \| |  | \| \| |  |  |  |  |  |  |  |  |
| 2 | Vitvi18g04227\_t001 |  | \| \| |  | \| \| |  |  |  |  |  |  |  |  |
| 2 | Vitvi18g00957\_t003 |  | Vitvi04g01287\_t001 |  | \| \| |  |  |  |  |  |  |  |  |
| 2 | Vitvi18g00958\_t001 |  | \| \| |  | Vitvi03g00334\_t001 |  |  |  |  |  |  |  |  |
| 2 | Vitvi18g04228\_t001 |  | \| \| |  | \| \| |  |  |  |  |  |  |  |  |
| 2 | Vitvi18g02748\_t001 |  | \| \| |  | \| \| |  |  |  |  |  |  |  |  |
| 2 | Vitvi18g00959\_t001 |  | \| \| |  | \| \| |  |  |  |  |  |  |  |  |
| 2 | Vitvi18g00960\_t001 |  | \| \| |  | \| \| |  |  |  |  |  |  |  |  |
| 2 | Vitvi18g00961\_t001 |  | \| \| |  | \| \| |  |  |  |  |  |  |  |  |
| 2 | Vitvi18g00962\_t001 |  | \| \| |  | \| \| |  |  |  |  |  |  |  |  |
| 2 | Vitvi18g00963\_t001 |  | \| \| |  | \| \| |  |  |  |  |  |  |  |  |
| 2 | Vitvi18g00964\_t001 |  | \| \| |  | \| \| |  |  |  |  |  |  |  |  |
| 2 | Vitvi18g00966\_t001 |  | \| \| |  | Vitvi03g00328\_t002 |  |  |  |  |  |  |  |  |
| 2 | Vitvi18g00967\_t001 |  | \| \| |  | \| \| |  |  |  |  |  |  |  |  |
| 2 | Vitvi18g00968\_t002 |  | Vitvi04g01296\_t001 |  | \| \| |  |  |  |  |  |  |  |  |
| 2 | Vitvi18g00969\_t001 |  | \| \| |  | Vitvi03g00322\_t001 |  |  |  |  |  |  |  |  |
| 2 | Vitvi18g00970\_t002 |  | Vitvi04g01302\_t001 |  | \| \| |  |  |  |  |  |  |  |  |
| 2 | Vitvi18g00971\_t001 |  | Vitvi04g01303\_t001 |  | Vitvi03g00319\_t001 |  |  |  |  |  |  |  |  |
| 2 | Vitvi18g00972\_t001 |  | Vitvi04g01304\_t001 |  | \| \| |  |  |  |  |  |  |  |  |
| 2 | Vitvi18g02749\_t001 |  | \| \| |  | \| \| |  |  |  |  |  |  |  |  |
| 2 | Vitvi18g02750\_t001 |  | \| \| |  | \| \| |  |  |  |  |  |  |  |  |
| 2 | Vitvi18g00973\_t001 |  | Vitvi04g01309\_t001 |  | Vitvi03g01495\_t001 |  |  |  |  |  |  |  |  |
| 2 | Vitvi18g00974\_t001 |  | Vitvi04g01311\_t001 |  | \| \| |  |  |  |  |  |  |  |  |
| 2 | Vitvi18g04229\_t001 |  | \| \| |  | \| \| |  |  |  |  |  |  |  |  |
| 2 | Vitvi18g00975\_t001 |  | \| \| |  | \| \| |  |  |  |  |  |  |  |  |
| 2 | Vitvi18g02752\_t001 |  | \| \| |  | \| \| |  |  |  |  |  |  |  |  |
| 2 | Vitvi18g02753\_t001 |  | \| \| |  | \| \| |  |  |  |  |  |  |  |  |
| 2 | Vitvi18g00976\_t001 |  | Vitvi04g01315\_t001 |  | \| \| |  |  |  |  |  |  |  |  |
| 2 | Vitvi18g04230\_t001 |  | \| \| |  | \| \| |  |  |  |  |  |  |  |  |
| 2 | Vitvi18g04231\_t001 |  | \| \| |  | \| \| |  |  |  |  |  |  |  |  |
| 2 | Vitvi18g00977\_t001 |  | \| \| |  | Vitvi03g00313\_t001 |  |  |  |  |  |  |  |  |
| 2 | Vitvi18g02754\_t002 |  | \| \| |  | \| \| |  |  |  |  |  |  |  |  |
| 2 | Vitvi18g00979\_t001 |  | \| \| |  | Vitvi03g01484\_t001 |  |  |  |  |  |  |  |  |
| 2 | Vitvi18g02755\_t001 |  | \| \| |  | \| \| |  |  |  |  |  |  |  |  |
| 2 | Vitvi18g02756\_t001 |  | \| \| |  | \| \| |  |  |  |  |  |  |  |  |
| 2 | Vitvi18g00980\_t001 |  | Vitvi04g01323\_t002 |  | Vitvi03g00307\_t003 |  |  |  |  |  |  |  |  |
| 2 | Vitvi18g00981\_t001 |  | Vitvi04g01325\_t001 |  | \| \| |  |  |  |  |  |  |  |  |
| 2 | Vitvi18g02757\_t001 |  | \| \| |  | \| \| |  |  |  |  |  |  |  |  |
| 2 | Vitvi18g00982\_t001 |  | \| \| |  | \| \| |  |  |  |  |  |  |  |  |
| 2 | Vitvi18g00983\_t001 |  | Vitvi04g01327\_t002 |  | \| \| |  |  |  |  |  |  |  |  |
| 2 | Vitvi18g00986\_t001 |  | \| \| |  | \| \| |  |  |  |  |  |  |  |  |
| 2 | Vitvi18g04232\_t001 |  | Vitvi04g04347\_t001 |  | \| \| |  |  |  |  |  |  |  |  |
| 2 | Vitvi18g00988\_t001 |  | \| \| |  | \| \| |  |  |  |  |  |  |  |  |
| 2 | Vitvi18g04233\_t001 |  | \| \| |  | \| \| |  |  |  |  |  |  |  |  |
| 2 | Vitvi18g00989\_t001 |  | \| \| |  | \| \| |  |  |  |  |  |  |  |  |
| 2 | Vitvi18g00990\_t001 |  | \| \| |  | \| \| |  |  |  |  |  |  |  |  |
| 2 | Vitvi18g00991\_t001 |  | Vitvi04g01334\_t001 |  | \| \| |  |  |  |  |  |  |  |  |
| 2 | Vitvi18g02758\_t002 |  | \| \| |  | Vitvi03g00304\_t001 |  |  |  |  |  |  |  |  |
| 2 | Vitvi18g02759\_t001 |  | \| \| |  | \| \| |  |  |  |  |  |  |  |  |
| 2 | Vitvi18g04234\_t001 |  | \| \| |  | \| \| |  |  |  |  |  |  |  |  |
| 2 | Vitvi18g02760\_t001 |  | \| \| |  | \| \| |  |  |  |  |  |  |  |  |
| 2 | Vitvi18g00992\_t001 |  | Vitvi04g01337\_t001 |  | Vitvi03g00302\_t001 |  |  |  |  |  |  |  |  |
| 2 | Vitvi18g00993\_t001 |  | \| \| |  | \| \| |  |  |  |  |  |  |  |  |
| 2 | Vitvi18g02761\_t002 |  | Vitvi04g02123\_t001 |  | \| \| |  |  |  |  |  |  |  |  |
| 2 | Vitvi18g02763\_t001 |  | \| \| |  | Vitvi03g00301\_t001 |  |  |  |  |  |  |  |  |
| 2 | Vitvi18g00994\_t001 |  | \| \| |  | \| \| |  |  |  |  |  |  |  |  |
| 2 | Vitvi18g00995\_t001 |  | Vitvi04g04361\_t001 |  | \| \| |  |  |  |  |  |  |  |  |
| 2 | Vitvi18g04235\_t001 |  | \| \| |  | \| \| |  |  |  |  |  |  |  |  |
| 2 | Vitvi18g00996\_t001 |  | \| \| |  | Vitvi03g04109\_t001 |  |  |  |  |  |  |  |  |
| 2 | Vitvi18g00997\_t001 |  | Vitvi04g01360\_t001 |  | Vitvi03g00294\_t001 |  |  |  |  |  |  |  |  |
| 2 | Vitvi18g00998\_t001 |  | \| \| |  | \| \| |  |  |  |  |  |  |  |  |
| 2 | Vitvi18g00999\_t001 |  | Vitvi04g01361\_t001 |  | \| \| |  |  |  |  |  |  |  |  |
| 2 | Vitvi18g01000\_t001 |  | \| \| |  | \| \| |  |  |  |  |  |  |  |  |
| 2 | Vitvi18g01001\_t001 |  | \| \| |  | \| \| |  |  |  |  |  |  |  |  |
| 2 | Vitvi18g04236\_t001 |  | \| \| |  | \| \| |  |  |  |  |  |  |  |  |
| 2 | Vitvi18g01002\_t001 |  | \| \| |  | \| \| |  |  |  |  |  |  |  |  |
| 2 | Vitvi18g04237\_t001 |  | \| \| |  | \| \| |  |  |  |  |  |  |  |  |
| 2 | Vitvi18g01004\_t001 |  | Vitvi04g01362\_t001 |  | \| \| |  |  |  |  |  |  |  |  |
| 2 | Vitvi18g01005\_t001 |  | Vitvi04g01363\_t001 |  | Vitvi03g00292\_t001 |  |  |  |  |  |  |  |  |
| 2 | Vitvi18g01006\_t003 |  | \| \| |  | \| \| |  |  |  |  |  |  |  |  |
| 2 | Vitvi18g04238\_t001 |  | \| \| |  | \| \| |  |  |  |  |  |  |  |  |
| 2 | Vitvi18g02767\_t001 |  | \| \| |  | Vitvi03g00288\_t001 |  |  |  |  |  |  |  |  |
| 2 | Vitvi18g01008\_t001 |  | \| \| |  | \| \| |  |  |  |  |  |  |  |  |
| 2 | Vitvi18g01010\_t001 |  | \| \| |  | \| \| |  |  |  |  |  |  |  |  |
| 2 | Vitvi18g01011\_t001 |  | \| \| |  | \| \| |  |  |  |  |  |  |  |  |
| 2 | Vitvi18g01012\_t001 |  | \| \| |  | Vitvi03g00285\_t001 |  |  |  |  |  |  |  |  |
| 1 | Vitvi18g01013\_t002 |  | Vitvi04g01371\_t001 |  |  |  |  |  |  |  |  |  |  |
| 1 | Vitvi18g01014\_t001 |  | Vitvi04g01378\_t001 |  |  |  |  |  |  |  |  |  |  |
| 1 | Vitvi18g01015\_t001 |  | \| \| |  |  |  |  |  |  |  |  |  |  |
| 2 | Vitvi18g02768\_t001 |  | \| \| |  | Vitvi03g00004\_t001 |  |  |  |  |  |  |  |  |
| 2 | Vitvi18g01016\_t004 |  | \| \| |  | \| \| |  |  |  |  |  |  |  |  |
| 2 | Vitvi18g04239\_t001 |  | \| \| |  | \| \| |  |  |  |  |  |  |  |  |
| 2 | Vitvi18g04240\_t001 |  | Vitvi04g02133\_t001 |  | \| \| |  |  |  |  |  |  |  |  |
| 2 | Vitvi18g01019\_t001 |  | \| \| |  | \| \| |  |  |  |  |  |  |  |  |
| 2 | Vitvi18g02773\_t001 |  | \| \| |  | \| \| |  |  |  |  |  |  |  |  |
| 2 | Vitvi18g04241\_t001 |  | \| \| |  | \| \| |  |  |  |  |  |  |  |  |
| 2 | Vitvi18g01020\_t001 |  | \| \| |  | \| \| |  |  |  |  |  |  |  |  |
| 2 | Vitvi18g01022\_t002 |  | Vitvi04g01389\_t001 |  | Vitvi03g00009\_t001 |  |  |  |  |  |  |  |  |
| 2 | Vitvi18g02775\_t001 |  | Vitvi04g01390\_t001 |  | \| \| |  |  |  |  |  |  |  |  |
| 2 | Vitvi18g01023\_t001 |  | \| \| |  | \| \| |  |  |  |  |  |  |  |  |
| 2 | Vitvi18g01024\_t001 |  | \| \| |  | \| \| |  |  |  |  |  |  |  |  |
| 2 | Vitvi18g01025\_t001 |  | \| \| |  | \| \| |  |  |  |  |  |  |  |  |
| 2 | Vitvi18g01027\_t001 |  | \| \| |  | \| \| |  |  |  |  |  |  |  |  |
| 2 | Vitvi18g02776\_t001 |  | \| \| |  | \| \| |  |  |  |  |  |  |  |  |
| 2 | Vitvi18g01028\_t001 |  | \| \| |  | \| \| |  |  |  |  |  |  |  |  |
| 2 | Vitvi18g01029\_t001 |  | \| \| |  | \| \| |  |  |  |  |  |  |  |  |
| 2 | Vitvi18g02777\_t001 |  | \| \| |  | Vitvi03g00015\_t001 |  |  |  |  |  |  |  |  |
| 2 | Vitvi18g02778\_t001 |  | \| \| |  | \| \| |  |  |  |  |  |  |  |  |
| 2 | Vitvi18g01031\_t001 |  | \| \| |  | \| \| |  |  |  |  |  |  |  |  |
| 2 | Vitvi18g01032\_t001 |  | \| \| |  | \| \| |  |  |  |  |  |  |  |  |
| 2 | Vitvi18g01034\_t001 |  | Vitvi04g01392\_t001 |  | \| \| |  |  |  |  |  |  |  |  |
| 2 | Vitvi18g01035\_t002 |  | \| \| |  | \| \| |  |  |  |  |  |  |  |  |
| 2 | Vitvi18g01036\_t001 |  | \| \| |  | Vitvi03g00021\_t001 |  |  |  |  |  |  |  |  |
| 2 | Vitvi18g01037\_t001 |  | \| \| |  | \| \| |  |  |  |  |  |  |  |  |
| 2 | Vitvi18g04242\_t001 |  | \| \| |  | \| \| |  |  |  |  |  |  |  |  |
| 2 | Vitvi18g02779\_t001 |  | \| \| |  | \| \| |  |  |  |  |  |  |  |  |
| 2 | Vitvi18g01038\_t001 |  | \| \| |  | Vitvi03g00022\_t001 |  |  |  |  |  |  |  |  |
| 2 | Vitvi18g01041\_t001 |  | Vitvi04g01399\_t001 |  | Vitvi03g00024\_t001 |  |  |  |  |  |  |  |  |
| 2 | Vitvi18g01042\_t001 |  | \| \| |  | \| \| |  |  |  |  |  |  |  |  |
| 2 | Vitvi18g01043\_t001 |  | \| \| |  | \| \| |  |  |  |  |  |  |  |  |
| 2 | Vitvi18g01044\_t001 |  | Vitvi04g01404\_t001 |  | \| \| |  |  |  |  |  |  |  |  |
| 2 | Vitvi18g01045\_t001 |  | Vitvi04g01406\_t001 |  | \| \| |  |  |  |  |  |  |  |  |
| 2 | Vitvi18g01047\_t001 |  | Vitvi04g01407\_t001 |  | \| \| |  |  |  |  |  |  |  |  |
| 2 | Vitvi18g01048\_t001 |  | Vitvi04g01423\_t003 |  | Vitvi03g00026\_t001 |  |  |  |  |  |  |  |  |
| 2 | Vitvi18g01049\_t001 |  | \| \| |  | Vitvi03g00035\_t001 |  |  |  |  |  |  |  |  |
| 2 | Vitvi18g02780\_t001 |  | \| \| |  | \| \| |  |  |  |  |  |  |  |  |
| 2 | Vitvi18g04243\_t001 |  | \| \| |  | \| \| |  |  |  |  |  |  |  |  |
| 2 | Vitvi18g01050\_t001 |  | \| \| |  | Vitvi03g00043\_t002 |  |  |  |  |  |  |  |  |
| 2 | Vitvi18g01051\_t001 |  | \| \| |  | \| \| |  |  |  |  |  |  |  |  |
| 2 | Vitvi18g01052\_t001 |  | \| \| |  | \| \| |  |  |  |  |  |  |  |  |
| 2 | Vitvi18g04244\_t001 |  | \| \| |  | \| \| |  |  |  |  |  |  |  |  |
| 2 | Vitvi18g01055\_t001 |  | Vitvi04g01425\_t003 |  | Vitvi03g00050\_t001 |  |  |  |  |  |  |  |  |
| 2 | Vitvi18g01056\_t001 |  | Vitvi04g01426\_t001 |  | \| \| |  |  |  |  |  |  |  |  |
| 2 | Vitvi18g01057\_t001 |  | \| \| |  | \| \| |  |  |  |  |  |  |  |  |
| 2 | Vitvi18g01058\_t001 |  | \| \| |  | Vitvi03g04022\_t001 |  |  |  |  |  |  |  |  |
| 2 | Vitvi18g04245\_t001 |  | \| \| |  | \| \| |  |  |  |  |  |  |  |  |
| 2 | Vitvi18g01059\_t001 |  | \| \| |  | \| \| |  |  |  |  |  |  |  |  |
| 2 | Vitvi18g04246\_t001 |  | \| \| |  | \| \| |  |  |  |  |  |  |  |  |
| 2 | Vitvi18g04247\_t001 |  | \| \| |  | \| \| |  |  |  |  |  |  |  |  |
| 2 | Vitvi18g01061\_t001 |  | \| \| |  | \| \| |  |  |  |  |  |  |  |  |
| 2 | Vitvi18g01063\_t001 |  | \| \| |  | \| \| |  |  |  |  |  |  |  |  |
| 2 | Vitvi18g04248\_t001 |  | Vitvi04g04384\_t001 |  | \| \| |  |  |  |  |  |  |  |  |
| 2 | Vitvi18g01065\_t001 |  | \| \| |  | \| \| |  |  |  |  |  |  |  |  |
| 2 | Vitvi18g04249\_t001 |  | \| \| |  | Vitvi03g00058\_t001 |  |  |  |  |  |  |  |  |
| 2 | Vitvi18g04250\_t001 |  | \| \| |  | \| \| |  |  |  |  |  |  |  |  |
| 2 | Vitvi18g01067\_t001 |  | \| \| |  | \| \| |  |  |  |  |  |  |  |  |
| 2 | Vitvi18g01068\_t001 |  | \| \| |  | Vitvi03g00059\_t001 |  |  |  |  |  |  |  |  |
| 2 | Vitvi18g01069\_t001 |  | \| \| |  | \| \| |  |  |  |  |  |  |  |  |
| 2 | Vitvi18g01070\_t001 |  | \| \| |  | \| \| |  |  |  |  |  |  |  |  |
| 2 | Vitvi18g04251\_t001 |  | \| \| |  | \| \| |  |  |  |  |  |  |  |  |
| 2 | Vitvi18g01072\_t001 |  | \| \| |  | \| \| |  |  |  |  |  |  |  |  |
| 2 | Vitvi18g01073\_t001 |  | \| \| |  | \| \| |  |  |  |  |  |  |  |  |
| 2 | Vitvi18g01078\_t001 |  | \| \| |  | \| \| |  |  |  |  |  |  |  |  |
| 2 | Vitvi18g01079\_t001 |  | \| \| |  | \| \| |  |  |  |  |  |  |  |  |
| 2 | Vitvi18g04252\_t001 |  | \| \| |  | \| \| |  |  |  |  |  |  |  |  |
| 2 | Vitvi18g04253\_t001 |  | \| \| |  | \| \| |  |  |  |  |  |  |  |  |
| 2 | Vitvi18g04254\_t001 |  | \| \| |  | \| \| |  |  |  |  |  |  |  |  |
| 2 | Vitvi18g01080\_t001 |  | \| \| |  | \| \| |  |  |  |  |  |  |  |  |
| 2 | Vitvi18g01081\_t001 |  | Vitvi04g01436\_t001 |  | \| \| |  |  |  |  |  |  |  |  |
| 2 | Vitvi18g01082\_t001 |  | \| \| |  | \| \| |  |  |  |  |  |  |  |  |
| 2 | Vitvi18g01083\_t001.1.6037826d |  | \| \| |  | \| \| |  |  |  |  |  |  |  |  |
| 2 | Vitvi18g01084\_t001 |  | \| \| |  | Vitvi03g01335\_t001 |  |  |  |  |  |  |  |  |
| 2 | Vitvi18g01085\_t001 |  | \| \| |  | \| \| |  |  |  |  |  |  |  |  |
| 2 | Vitvi18g01086\_t001 |  | \| \| |  | \| \| |  |  |  |  |  |  |  |  |
| 2 | Vitvi18g04255\_t001 |  | \| \| |  | \| \| |  |  |  |  |  |  |  |  |
| 2 | Vitvi18g01088\_t001 |  | \| \| |  | \| \| |  |  |  |  |  |  |  |  |
| 2 | Vitvi18g01090\_t001 |  | \| \| |  | \| \| |  |  |  |  |  |  |  |  |
| 2 | Vitvi18g04256\_t001 |  | \| \| |  | \| \| |  |  |  |  |  |  |  |  |
| 2 | Vitvi18g01091\_t001 |  | \| \| |  | Vitvi03g01336\_t001 |  |  |  |  |  |  |  |  |
| 2 | Vitvi18g04257\_t001 |  | \| \| |  | \| \| |  |  |  |  |  |  |  |  |
| 2 | Vitvi18g01092\_t001.2.6037826c |  | Vitvi04g01440\_t001 |  | \| \| |  |  |  |  |  |  |  |  |
| 2 | Vitvi18g01093\_t001 |  | Vitvi04g01441\_t001 |  | Vitvi03g00064\_t001 |  |  |  |  |  |  |  |  |
| 1 | Vitvi18g04258\_t001 |  | \| \| |  |  |  |  |  |  |  |  |  |  |
| 2 | Vitvi18g01094\_t001 |  | \| \| |  | Vitvi03g04034\_t001 |  |  |  |  |  |  |  |  |
| 2 | Vitvi18g01095\_t001 |  | \| \| |  | \| \| |  |  |  |  |  |  |  |  |
| 2 | Vitvi18g01096\_t001 |  | \| \| |  | \| \| |  |  |  |  |  |  |  |  |
| 2 | Vitvi18g01097\_t001 |  | \| \| |  | \| \| |  |  |  |  |  |  |  |  |
| 2 | Vitvi18g01098\_t001 |  | Vitvi04g01444\_t001 |  | \| \| |  |  |  |  |  |  |  |  |
| 2 | Vitvi18g04259\_t001 |  | \| \| |  | \| \| |  |  |  |  |  |  |  |  |
| 2 | Vitvi18g04260\_t001 |  | \| \| |  | \| \| |  |  |  |  |  |  |  |  |
| 2 | Vitvi18g01101\_t001 |  | \| \| |  | Vitvi03g00078\_t001 |  |  |  |  |  |  |  |  |
| 2 | Vitvi18g04261\_t001 |  | \| \| |  | \| \| |  |  |  |  |  |  |  |  |
| 2 | Vitvi18g01102\_t001 |  | \| \| |  | \| \| |  |  |  |  |  |  |  |  |
| 2 | Vitvi18g04262\_t001 |  | \| \| |  | \| \| |  |  |  |  |  |  |  |  |
| 2 | Vitvi18g01103\_t001 |  | \| \| |  | \| \| |  |  |  |  |  |  |  |  |
| 2 | Vitvi18g01104\_t001 |  | \| \| |  | \| \| |  |  |  |  |  |  |  |  |
| 2 | Vitvi18g01105\_t002 |  | \| \| |  | Vitvi03g00079\_t001 |  |  |  |  |  |  |  |  |
| 2 | Vitvi18g01106\_t001 |  | \| \| |  | \| \| |  |  |  |  |  |  |  |  |
| 2 | Vitvi18g01107\_t001 |  | \| \| |  | \| \| |  |  |  |  |  |  |  |  |
| 2 | Vitvi18g01109\_t001 |  | \| \| |  | \| \| |  |  |  |  |  |  |  |  |
| 2 | Vitvi18g02788\_t001 |  | \| \| |  | \| \| |  |  |  |  |  |  |  |  |
| 2 | Vitvi18g02790\_t001 |  | \| \| |  | \| \| |  |  |  |  |  |  |  |  |
| 2 | Vitvi18g01110\_t001 |  | \| \| |  | \| \| |  |  |  |  |  |  |  |  |
| 2 | Vitvi18g01111\_t001 |  | \| \| |  | \| \| |  |  |  |  |  |  |  |  |
| 2 | Vitvi18g04263\_t001 |  | \| \| |  | \| \| |  |  |  |  |  |  |  |  |
| 2 | Vitvi18g04264\_t001 |  | \| \| |  | \| \| |  |  |  |  |  |  |  |  |
| 2 | Vitvi18g02792\_t001 |  | Vitvi04g02150\_t001 |  | \| \| |  |  |  |  |  |  |  |  |
| 2 | Vitvi18g04265\_t001 |  | \| \| |  | \| \| |  |  |  |  |  |  |  |  |
| 2 | Vitvi18g01114\_t001 |  | \| \| |  | \| \| |  |  |  |  |  |  |  |  |
| 2 | Vitvi18g02794\_t001 |  | Vitvi04g01451\_t001 |  | \| \| |  |  |  |  |  |  |  |  |
| 2 | Vitvi18g01115\_t001 |  | \| \| |  | \| \| |  |  |  |  |  |  |  |  |
| 2 | Vitvi18g04266\_t001 |  | \| \| |  | \| \| |  |  |  |  |  |  |  |  |
| 2 | Vitvi18g02795\_t001 |  | \| \| |  | \| \| |  |  |  |  |  |  |  |  |
| 2 | Vitvi18g02797\_t001 |  | \| \| |  | \| \| |  |  |  |  |  |  |  |  |
| 2 | Vitvi18g01116\_t001 |  | \| \| |  | Vitvi03g00087\_t001 |  |  |  |  |  |  |  |  |
| 2 | Vitvi18g01117\_t001 |  | \| \| |  | Vitvi03g00088\_t001 |  |  |  |  |  |  |  |  |
| 2 | Vitvi18g04267\_t001 |  | \| \| |  | \| \| |  |  |  |  |  |  |  |  |
| 2 | Vitvi18g01119\_t001 |  | Vitvi04g01454\_t001 |  | \| \| |  |  |  |  |  |  |  |  |
| 2 | Vitvi18g02798\_t001 |  | \| \| |  | Vitvi03g01372\_t001 |  |  |  |  |  |  |  |  |
| 1 | Vitvi18g02799\_t001 |  | \| \| |  |  |  |  |  |  |  |  |  |  |
| 1 | Vitvi18g02800\_t001 |  | \| \| |  |  |  |  |  |  |  |  |  |  |
| 1 | Vitvi18g02801\_t001 |  | \| \| |  |  |  |  |  |  |  |  |  |  |
| 1 | Vitvi18g04268\_t001 |  | \| \| |  |  |  |  |  |  |  |  |  |  |
| 1 | Vitvi18g02802\_t001 |  | \| \| |  |  |  |  |  |  |  |  |  |  |
| 1 | Vitvi18g01120\_t001 |  | \| \| |  |  |  |  |  |  |  |  |  |  |
| 1 | Vitvi18g01122\_t001 |  | \| \| |  |  |  |  |  |  |  |  |  |  |
| 1 | Vitvi18g01123\_t001 |  | \| \| |  |  |  |  |  |  |  |  |  |  |
| 1 | Vitvi18g01126\_t001 |  | \| \| |  |  |  |  |  |  |  |  |  |  |
| 1 | Vitvi18g02803\_t001 |  | \| \| |  |  |  |  |  |  |  |  |  |  |
| 1 | Vitvi18g02804\_t001 |  | \| \| |  |  |  |  |  |  |  |  |  |  |
| 1 | Vitvi18g01128\_t001 |  | \| \| |  |  |  |  |  |  |  |  |  |  |
| 1 | Vitvi18g04269\_t001 |  | \| \| |  |  |  |  |  |  |  |  |  |  |
| 2 | Vitvi18g01129\_t001 |  | Vitvi04g01466\_t001 |  | Vitvi03g00119\_t001 |  |  |  |  |  |  |  |  |
| 2 | Vitvi18g01130\_t001 |  | Vitvi04g01468\_t001 |  | \| \| |  |  |  |  |  |  |  |  |
| 2 | Vitvi18g02805\_t001 |  | \| \| |  | \| \| |  |  |  |  |  |  |  |  |
| 2 | Vitvi18g01131\_t001 |  | Vitvi04g01470\_t001 |  | Vitvi03g00123\_t001 |  |  |  |  |  |  |  |  |
| 1 | Vitvi18g04270\_t001 |  |  |  | \| \| |  |  |  |  |  |  |  |  |
| 1 | Vitvi18g01132\_t001 |  |  |  | Vitvi03g00124\_t001 |  |  |  |  |  |  |  |  |
| 1 | Vitvi18g01133\_t001 |  |  |  | \| \| |  |  |  |  |  |  |  |  |
| 1 | Vitvi18g01134\_t001 |  |  |  | \| \| |  |  |  |  |  |  |  |  |
| 1 | Vitvi18g01135\_t001 |  |  |  | \| \| |  |  |  |  |  |  |  |  |
| 1 | Vitvi18g01136\_t001 |  |  |  | \| \| |  |  |  |  |  |  |  |  |
| 1 | Vitvi18g01137\_t001 |  |  |  | \| \| |  |  |  |  |  |  |  |  |
| 1 | Vitvi18g01139\_t001 |  |  |  | Vitvi03g00131\_t001 |  |  |  |  |  |  |  |  |
| 1 | Vitvi18g01142\_t001 |  |  |  | \| \| |  |  |  |  |  |  |  |  |
| 1 | Vitvi18g04271\_t001 |  |  |  | \| \| |  |  |  |  |  |  |  |  |
| 1 | Vitvi18g02810\_t001 |  |  |  | \| \| |  |  |  |  |  |  |  |  |
| 1 | Vitvi18g01144\_t001 |  |  |  | \| \| |  |  |  |  |  |  |  |  |
| 1 | Vitvi18g01146\_t001 |  |  |  | \| \| |  |  |  |  |  |  |  |  |
| 1 | Vitvi18g01147\_t001 |  |  |  | Vitvi03g00138\_t001 |  |  |  |  |  |  |  |  |
| 1 | Vitvi18g02812\_t001 |  |  |  | \| \| |  |  |  |  |  |  |  |  |
| 1 | Vitvi18g02813\_t001 |  |  |  | \| \| |  |  |  |  |  |  |  |  |
| 1 | Vitvi18g01148\_t001 |  |  |  | \| \| |  |  |  |  |  |  |  |  |
| 2 | Vitvi18g01149\_t001 |  | Vitvi04g01498\_t001 |  | Vitvi03g00143\_t001 |  |  |  |  |  |  |  |  |
| 2 | Vitvi18g01150\_t001 |  | Vitvi04g01499\_t001 |  | \| \| |  |  |  |  |  |  |  |  |
| 2 | Vitvi18g01152\_t001 |  | \| \| |  | Vitvi03g00144\_t001 |  |  |  |  |  |  |  |  |
| 2 | Vitvi18g02814\_t002 |  | Vitvi04g01500\_t001 |  | Vitvi03g01400\_t001 |  |  |  |  |  |  |  |  |
| 2 | Vitvi18g01153\_t001 |  | \| \| |  | \| \| |  |  |  |  |  |  |  |  |
| 2 | Vitvi18g01155\_t001 |  | \| \| |  | \| \| |  |  |  |  |  |  |  |  |
| 2 | Vitvi18g01156\_t001 |  | \| \| |  | Vitvi03g00149\_t001 |  |  |  |  |  |  |  |  |
| 2 | Vitvi18g01157\_t001 |  | Vitvi04g04396\_t001 |  | \| \| |  |  |  |  |  |  |  |  |
| 2 | Vitvi18g04272\_t001 |  | \| \| |  | \| \| |  |  |  |  |  |  |  |  |
| 2 | Vitvi18g02816\_t001 |  | \| \| |  | \| \| |  |  |  |  |  |  |  |  |
| 2 | Vitvi18g02817\_t001 |  | \| \| |  | \| \| |  |  |  |  |  |  |  |  |
| 2 | Vitvi18g02818\_t001 |  | \| \| |  | \| \| |  |  |  |  |  |  |  |  |
| 2 | Vitvi18g02819\_t001 |  | \| \| |  | \| \| |  |  |  |  |  |  |  |  |
| 2 | Vitvi18g02820\_t001 |  | \| \| |  | \| \| |  |  |  |  |  |  |  |  |
| 2 | Vitvi18g01159\_t001 |  | \| \| |  | Vitvi03g00151\_t001 |  |  |  |  |  |  |  |  |
| 2 | Vitvi18g01160\_t002 |  | \| \| |  | \| \| |  |  |  |  |  |  |  |  |
| 2 | Vitvi18g01161\_t001 |  | Vitvi04g04398\_t001 |  | Vitvi03g00152\_t001 |  |  |  |  |  |  |  |  |
| 1 | Vitvi18g01162\_t001 |  | \| \| |  |  |  |  |  |  |  |  |  |  |
| 1 | Vitvi18g01163\_t003 |  | \| \| |  |  |  |  |  |  |  |  |  |  |
| 1 | Vitvi18g01165\_t001 |  | Vitvi04g01505\_t001 |  |  |  |  |  |  |  |  |  |  |
| 1 | Vitvi18g01166\_t001 |  | Vitvi04g01506\_t001 |  |  |  |  |  |  |  |  |  |  |
| 0 | Vitvi18g01167\_t001 |  |  |  |  |  |  |  |  |  |  |  |  |
| 0 | Vitvi18g01168\_t001 |  |  |  |  |  |  |  |  |  |  |  |  |
| 0 | Vitvi18g04273\_t001 |  |  |  |  |  |  |  |  |  |  |  |  |
| 0 | Vitvi18g01169\_t001 |  |  |  |  |  |  |  |  |  |  |  |  |
| 0 | Vitvi18g01170\_t001 |  |  |  |  |  |  |  |  |  |  |  |  |
| 0 | Vitvi18g01171\_t001 |  |  |  |  |  |  |  |  |  |  |  |  |
| 0 | Vitvi18g01172\_t001 |  |  |  |  |  |  |  |  |  |  |  |  |
| 0 | Vitvi18g01173\_t001 |  |  |  |  |  |  |  |  |  |  |  |  |
| 1 | Vitvi18g01174\_t001 |  | Vitvi04g01711\_t001 |  |  |  |  |  |  |  |  |  |  |
| 1 | Vitvi18g01175\_t001 |  | \| \| |  |  |  |  |  |  |  |  |  |  |
| 1 | Vitvi18g01177\_t001 |  | \| \| |  |  |  |  |  |  |  |  |  |  |
| 1 | Vitvi18g01179\_t001 |  | \| \| |  |  |  |  |  |  |  |  |  |  |
| 1 | Vitvi18g01180\_t001 |  | \| \| |  |  |  |  |  |  |  |  |  |  |
| 1 | Vitvi18g01181\_t002 |  | \| \| |  |  |  |  |  |  |  |  |  |  |
| 1 | Vitvi18g01182\_t001 |  | \| \| |  |  |  |  |  |  |  |  |  |  |
| 1 | Vitvi18g01183\_t002 |  | \| \| |  |  |  |  |  |  |  |  |  |  |
| 1 | Vitvi18g01184\_t001 |  | \| \| |  |  |  |  |  |  |  |  |  |  |
| 1 | Vitvi18g02821\_t001 |  | \| \| |  |  |  |  |  |  |  |  |  |  |
| 1 | Vitvi18g01187\_t001 |  | \| \| |  |  |  |  |  |  |  |  |  |  |
| 1 | Vitvi18g01188\_t001 |  | \| \| |  |  |  |  |  |  |  |  |  |  |
| 1 | Vitvi18g01189\_t001 |  | \| \| |  |  |  |  |  |  |  |  |  |  |
| 1 | Vitvi18g01190\_t001 |  | \| \| |  |  |  |  |  |  |  |  |  |  |
| 1 | Vitvi18g02823\_t001 |  | Vitvi04g01702\_t001 |  |  |  |  |  |  |  |  |  |  |
| 1 | Vitvi18g02824\_t001 |  | \| \| |  |  |  |  |  |  |  |  |  |  |
| 1 | Vitvi18g04274\_t001 |  | \| \| |  |  |  |  |  |  |  |  |  |  |
| 1 | Vitvi18g02825\_t001 |  | \| \| |  |  |  |  |  |  |  |  |  |  |
| 1 | Vitvi18g04275\_t001 |  | \| \| |  |  |  |  |  |  |  |  |  |  |
| 1 | Vitvi18g01196\_t001 |  | \| \| |  |  |  |  |  |  |  |  |  |  |
| 1 | Vitvi18g04276\_t001 |  | \| \| |  |  |  |  |  |  |  |  |  |  |
| 1 | Vitvi18g01198\_t001 |  | \| \| |  |  |  |  |  |  |  |  |  |  |
| 1 | Vitvi18g04277\_t001 |  | \| \| |  |  |  |  |  |  |  |  |  |  |
| 1 | Vitvi18g01199\_t001 |  | \| \| |  |  |  |  |  |  |  |  |  |  |
| 1 | Vitvi18g01200\_t001 |  | \| \| |  |  |  |  |  |  |  |  |  |  |
| 1 | Vitvi18g01202\_t001 |  | \| \| |  |  |  |  |  |  |  |  |  |  |
| 1 | Vitvi18g01203\_t001 |  | \| \| |  |  |  |  |  |  |  |  |  |  |
| 1 | Vitvi18g01205\_t001 |  | \| \| |  |  |  |  |  |  |  |  |  |  |
| 1 | Vitvi18g01208\_t001 |  | \| \| |  |  |  |  |  |  |  |  |  |  |
| 1 | Vitvi18g01209\_t001 |  | Vitvi04g01697\_t001 |  |  |  |  |  |  |  |  |  |  |
| 1 | Vitvi18g04278\_t001 |  | \| \| |  |  |  |  |  |  |  |  |  |  |
| 1 | Vitvi18g01210\_t001 |  | Vitvi04g01696\_t001 |  |  |  |  |  |  |  |  |  |  |
| 1 | Vitvi18g04279\_t001 |  | \| \| |  |  |  |  |  |  |  |  |  |  |
| 1 | Vitvi18g04280\_t001 |  | \| \| |  |  |  |  |  |  |  |  |  |  |
| 1 | Vitvi18g01211\_t001 |  | \| \| |  |  |  |  |  |  |  |  |  |  |
| 1 | Vitvi18g01212\_t001 |  | \| \| |  |  |  |  |  |  |  |  |  |  |
| 1 | Vitvi18g01213\_t001 |  | \| \| |  |  |  |  |  |  |  |  |  |  |
| 1 | Vitvi18g01214\_t001 |  | Vitvi04g04505\_t001 |  |  |  |  |  |  |  |  |  |  |
| 1 | Vitvi18g04281\_t001 |  | \| \| |  |  |  |  |  |  |  |  |  |  |
| 1 | Vitvi18g01215\_t001 |  | \| \| |  |  |  |  |  |  |  |  |  |  |
| 1 | Vitvi18g01216\_t001 |  | \| \| |  |  |  |  |  |  |  |  |  |  |
| 1 | Vitvi18g01217\_t001 |  | \| \| |  |  |  |  |  |  |  |  |  |  |
| 1 | Vitvi18g01218\_t001 |  | \| \| |  |  |  |  |  |  |  |  |  |  |
| 1 | Vitvi18g01219\_t001 |  | \| \| |  |  |  |  |  |  |  |  |  |  |
| 1 | Vitvi18g01220\_t001 |  | \| \| |  |  |  |  |  |  |  |  |  |  |
| 1 | Vitvi18g01221\_t001 |  | \| \| |  |  |  |  |  |  |  |  |  |  |
| 1 | Vitvi18g02832\_t001 |  | \| \| |  |  |  |  |  |  |  |  |  |  |
| 1 | Vitvi18g01223\_t001 |  | Vitvi04g01670\_t001 |  |  |  |  |  |  |  |  |  |  |
| 1 | Vitvi18g01224\_t001 |  | \| \| |  |  |  |  |  |  |  |  |  |  |
| 1 | Vitvi18g01225\_t001 |  | \| \| |  |  |  |  |  |  |  |  |  |  |
| 1 | Vitvi18g04282\_t001 |  | \| \| |  |  |  |  |  |  |  |  |  |  |
| 1 | Vitvi18g01226\_t001 |  | \| \| |  |  |  |  |  |  |  |  |  |  |
| 1 | Vitvi18g01230\_t001 |  | \| \| |  |  |  |  |  |  |  |  |  |  |
| 1 | Vitvi18g01231\_t001 |  | \| \| |  |  |  |  |  |  |  |  |  |  |
| 1 | Vitvi18g02833\_t001 |  | Vitvi04g02248\_t001 |  |  |  |  |  |  |  |  |  |  |
| 1 | Vitvi18g01232\_t001 |  | \| \| |  |  |  |  |  |  |  |  |  |  |
| 1 | Vitvi18g04283\_t001 |  | \| \| |  |  |  |  |  |  |  |  |  |  |
| 1 | Vitvi18g01235\_t004 |  | Vitvi04g01663\_t001 |  |  |  |  |  |  |  |  |  |  |
| 1 | Vitvi18g01236\_t001 |  | \| \| |  |  |  |  |  |  |  |  |  |  |
| 1 | Vitvi18g01237\_t001 |  | \| \| |  |  |  |  |  |  |  |  |  |  |
| 1 | Vitvi18g02835\_t001 |  | \| \| |  |  |  |  |  |  |  |  |  |  |
| 1 | Vitvi18g01238\_t001 |  | \| \| |  |  |  |  |  |  |  |  |  |  |
| 1 | Vitvi18g01239\_t001 |  | \| \| |  |  |  |  |  |  |  |  |  |  |
| 1 | Vitvi18g01240\_t003 |  | Vitvi04g01661\_t001 |  |  |  |  |  |  |  |  |  |  |
| 1 | Vitvi18g01242\_t002 |  | Vitvi04g01657\_t001 |  |  |  |  |  |  |  |  |  |  |
| 1 | Vitvi18g01243\_t001 |  | \| \| |  |  |  |  |  |  |  |  |  |  |
| 1 | Vitvi18g01244\_t001 |  | Vitvi04g01654\_t001 |  |  |  |  |  |  |  |  |  |  |
| 0 | Vitvi18g01245\_t001 |  |  |  |  |  |  |  |  |  |  |  |  |
| 0 | Vitvi18g01247\_t001 |  |  |  |  |  |  |  |  |  |  |  |  |
| 0 | Vitvi18g02836\_t001 |  |  |  |  |  |  |  |  |  |  |  |  |
| 0 | Vitvi18g01248\_t001 |  |  |  |  |  |  |  |  |  |  |  |  |
| 0 | Vitvi18g02837\_t001.1.6037826c |  |  |  |  |  |  |  |  |  |  |  |  |
| 0 | Vitvi18g01249\_t002 |  |  |  |  |  |  |  |  |  |  |  |  |
| 0 | Vitvi18g01250\_t001 |  |  |  |  |  |  |  |  |  |  |  |  |
| 0 | Vitvi18g01251\_t001 |  |  |  |  |  |  |  |  |  |  |  |  |
| 0 | Vitvi18g01252\_t001 |  |  |  |  |  |  |  |  |  |  |  |  |
| 0 | Vitvi18g01253\_t001 |  |  |  |  |  |  |  |  |  |  |  |  |
| 0 | Vitvi18g01254\_t001 |  |  |  |  |  |  |  |  |  |  |  |  |
| 0 | Vitvi18g04284\_t001 |  |  |  |  |  |  |  |  |  |  |  |  |
| 0 | Vitvi18g01256\_t001 |  |  |  |  |  |  |  |  |  |  |  |  |
| 0 | Vitvi18g02839\_t001 |  |  |  |  |  |  |  |  |  |  |  |  |
| 0 | Vitvi18g01257\_t001 |  |  |  |  |  |  |  |  |  |  |  |  |
| 0 | Vitvi18g01258\_t001 |  |  |  |  |  |  |  |  |  |  |  |  |
| 0 | Vitvi18g04285\_t001 |  |  |  |  |  |  |  |  |  |  |  |  |
| 0 | Vitvi18g04286\_t001 |  |  |  |  |  |  |  |  |  |  |  |  |
| 0 | Vitvi18g04287\_t001 |  |  |  |  |  |  |  |  |  |  |  |  |
| 0 | Vitvi18g04288\_t001 |  |  |  |  |  |  |  |  |  |  |  |  |
| 0 | Vitvi18g04289\_t001 |  |  |  |  |  |  |  |  |  |  |  |  |
| 0 | Vitvi18g01260\_t001 |  |  |  |  |  |  |  |  |  |  |  |  |
| 0 | Vitvi18g01261\_t001 |  |  |  |  |  |  |  |  |  |  |  |  |
| 0 | Vitvi18g04290\_t001 |  |  |  |  |  |  |  |  |  |  |  |  |
| 0 | Vitvi18g04291\_t001 |  |  |  |  |  |  |  |  |  |  |  |  |
| 0 | Vitvi18g04292\_t001 |  |  |  |  |  |  |  |  |  |  |  |  |
| 0 | Vitvi18g01262\_t001 |  |  |  |  |  |  |  |  |  |  |  |  |
| 0 | Vitvi18g04293\_t001 |  |  |  |  |  |  |  |  |  |  |  |  |
| 0 | Vitvi18g01264\_t001 |  |  |  |  |  |  |  |  |  |  |  |  |
| 0 | Vitvi18g01265\_t001 |  |  |  |  |  |  |  |  |  |  |  |  |
| 0 | Vitvi18g01266\_t001 |  |  |  |  |  |  |  |  |  |  |  |  |
| 0 | Vitvi18g04294\_t001 |  |  |  |  |  |  |  |  |  |  |  |  |
| 0 | Vitvi18g01267\_t001 |  |  |  |  |  |  |  |  |  |  |  |  |
| 0 | Vitvi18g01268\_t001 |  |  |  |  |  |  |  |  |  |  |  |  |
| 0 | Vitvi18g01270\_t001 |  |  |  |  |  |  |  |  |  |  |  |  |
| 0 | Vitvi18g01272\_t001 |  |  |  |  |  |  |  |  |  |  |  |  |
| 0 | Vitvi18g02846\_t001 |  |  |  |  |  |  |  |  |  |  |  |  |
| 0 | Vitvi18g02847\_t001 |  |  |  |  |  |  |  |  |  |  |  |  |
| 0 | Vitvi18g01275\_t002 |  |  |  |  |  |  |  |  |  |  |  |  |
| 0 | Vitvi18g01276\_t001 |  |  |  |  |  |  |  |  |  |  |  |  |
| 0 | Vitvi18g01277\_t001 |  |  |  |  |  |  |  |  |  |  |  |  |
| 0 | Vitvi18g04295\_t001 |  |  |  |  |  |  |  |  |  |  |  |  |
| 0 | Vitvi18g04296\_t001 |  |  |  |  |  |  |  |  |  |  |  |  |
| 0 | Vitvi18g01280\_t001 |  |  |  |  |  |  |  |  |  |  |  |  |
| 0 | Vitvi18g01281\_t001 |  |  |  |  |  |  |  |  |  |  |  |  |
| 0 | Vitvi18g01284\_t001 |  |  |  |  |  |  |  |  |  |  |  |  |
| 0 | Vitvi18g01285\_t001 |  |  |  |  |  |  |  |  |  |  |  |  |
| 0 | Vitvi18g02848\_t001 |  |  |  |  |  |  |  |  |  |  |  |  |
| 0 | Vitvi18g04297\_t001 |  |  |  |  |  |  |  |  |  |  |  |  |
| 0 | Vitvi18g04298\_t001 |  |  |  |  |  |  |  |  |  |  |  |  |
| 0 | Vitvi18g01288\_t001 |  |  |  |  |  |  |  |  |  |  |  |  |
| 0 | Vitvi18g01289\_t001 |  |  |  |  |  |  |  |  |  |  |  |  |
| 0 | Vitvi18g01290\_t002 |  |  |  |  |  |  |  |  |  |  |  |  |
| 0 | Vitvi18g04299\_t001 |  |  |  |  |  |  |  |  |  |  |  |  |
| 0 | Vitvi18g04300\_t001 |  |  |  |  |  |  |  |  |  |  |  |  |
| 0 | Vitvi18g01292\_t001 |  |  |  |  |  |  |  |  |  |  |  |  |
| 0 | Vitvi18g04301\_t001 |  |  |  |  |  |  |  |  |  |  |  |  |
| 0 | Vitvi18g04302\_t001 |  |  |  |  |  |  |  |  |  |  |  |  |
| 0 | Vitvi18g01295\_t001 |  |  |  |  |  |  |  |  |  |  |  |  |
| 0 | Vitvi18g01297\_t001 |  |  |  |  |  |  |  |  |  |  |  |  |
| 0 | Vitvi18g04303\_t001 |  |  |  |  |  |  |  |  |  |  |  |  |
| 0 | Vitvi18g01299\_t001 |  |  |  |  |  |  |  |  |  |  |  |  |
| 0 | Vitvi18g04304\_t001 |  |  |  |  |  |  |  |  |  |  |  |  |
| 0 | Vitvi18g04305\_t001 |  |  |  |  |  |  |  |  |  |  |  |  |
| 0 | Vitvi18g04306\_t001 |  |  |  |  |  |  |  |  |  |  |  |  |
| 0 | Vitvi18g01301\_t001 |  |  |  |  |  |  |  |  |  |  |  |  |
| 0 | Vitvi18g01304\_t001 |  |  |  |  |  |  |  |  |  |  |  |  |
| 0 | Vitvi18g01305\_t002 |  |  |  |  |  |  |  |  |  |  |  |  |
| 0 | Vitvi18g01307\_t001 |  |  |  |  |  |  |  |  |  |  |  |  |
| 0 | Vitvi18g01309\_t001 |  |  |  |  |  |  |  |  |  |  |  |  |
| 0 | Vitvi18g04307\_t001 |  |  |  |  |  |  |  |  |  |  |  |  |
| 0 | Vitvi18g01313\_t001 |  |  |  |  |  |  |  |  |  |  |  |  |
| 0 | Vitvi18g02853\_t001 |  |  |  |  |  |  |  |  |  |  |  |  |
| 0 | Vitvi18g04308\_t001 |  |  |  |  |  |  |  |  |  |  |  |  |
| 0 | Vitvi18g01314\_t001 |  |  |  |  |  |  |  |  |  |  |  |  |
| 0 | Vitvi18g01315\_t001 |  |  |  |  |  |  |  |  |  |  |  |  |
| 0 | Vitvi18g01320\_t001 |  |  |  |  |  |  |  |  |  |  |  |  |
| 0 | Vitvi18g01321\_t001 |  |  |  |  |  |  |  |  |  |  |  |  |
| 0 | Vitvi18g01322\_t001 |  |  |  |  |  |  |  |  |  |  |  |  |
| 0 | Vitvi18g04309\_t001 |  |  |  |  |  |  |  |  |  |  |  |  |
| 0 | Vitvi18g04310\_t001 |  |  |  |  |  |  |  |  |  |  |  |  |
| 0 | Vitvi18g04311\_t001 |  |  |  |  |  |  |  |  |  |  |  |  |
| 0 | Vitvi18g04313\_t001 |  |  |  |  |  |  |  |  |  |  |  |  |
| 0 | Vitvi18g04314\_t001 |  |  |  |  |  |  |  |  |  |  |  |  |
| 0 | Vitvi18g04315\_t001 |  |  |  |  |  |  |  |  |  |  |  |  |
| 0 | Vitvi18g04316\_t001 |  |  |  |  |  |  |  |  |  |  |  |  |
| 0 | Vitvi18g04317\_t001 |  |  |  |  |  |  |  |  |  |  |  |  |
| 0 | Vitvi18g04318\_t001 |  |  |  |  |  |  |  |  |  |  |  |  |
| 0 | Vitvi18g04319\_t001 |  |  |  |  |  |  |  |  |  |  |  |  |
| 0 | Vitvi18g04320\_t001 |  |  |  |  |  |  |  |  |  |  |  |  |
| 0 | Vitvi18g04321\_t001 |  |  |  |  |  |  |  |  |  |  |  |  |
| 0 | Vitvi18g01427\_t002 |  |  |  |  |  |  |  |  |  |  |  |  |
| 0 | Vitvi18g02890\_t001 |  |  |  |  |  |  |  |  |  |  |  |  |
| 0 | Vitvi18g01426\_t001 |  |  |  |  |  |  |  |  |  |  |  |  |
| 0 | Vitvi18g02889\_t001 |  |  |  |  |  |  |  |  |  |  |  |  |
| 0 | Vitvi18g04322\_t001 |  |  |  |  |  |  |  |  |  |  |  |  |
| 0 | Vitvi18g01422\_t001 |  |  |  |  |  |  |  |  |  |  |  |  |
| 0 | Vitvi18g01419\_t001 |  |  |  |  |  |  |  |  |  |  |  |  |
| 0 | Vitvi18g04323\_t001 |  |  |  |  |  |  |  |  |  |  |  |  |
| 0 | Vitvi18g04324\_t001 |  |  |  |  |  |  |  |  |  |  |  |  |
| 0 | Vitvi18g04325\_t001 |  |  |  |  |  |  |  |  |  |  |  |  |
| 0 | Vitvi18g04326\_t001 |  |  |  |  |  |  |  |  |  |  |  |  |
| 0 | Vitvi18g01411\_t001 |  |  |  |  |  |  |  |  |  |  |  |  |
| 0 | Vitvi18g02886\_t001 |  |  |  |  |  |  |  |  |  |  |  |  |
| 0 | Vitvi18g02885\_t001 |  |  |  |  |  |  |  |  |  |  |  |  |
| 0 | Vitvi18g02884\_t001 |  |  |  |  |  |  |  |  |  |  |  |  |
| 0 | Vitvi18g02883\_t001 |  |  |  |  |  |  |  |  |  |  |  |  |
| 0 | Vitvi18g04327\_t001 |  |  |  |  |  |  |  |  |  |  |  |  |
| 0 | Vitvi18g04328\_t001 |  |  |  |  |  |  |  |  |  |  |  |  |
| 0 | Vitvi18g04329\_t001 |  |  |  |  |  |  |  |  |  |  |  |  |
| 0 | Vitvi18g04330\_t001 |  |  |  |  |  |  |  |  |  |  |  |  |
| 0 | Vitvi18g02881\_t001 |  |  |  |  |  |  |  |  |  |  |  |  |
| 0 | Vitvi18g04331\_t001 |  |  |  |  |  |  |  |  |  |  |  |  |
| 0 | Vitvi18g02879\_t001 |  |  |  |  |  |  |  |  |  |  |  |  |
| 0 | Vitvi18g04332\_t001 |  |  |  |  |  |  |  |  |  |  |  |  |
| 0 | Vitvi18g04333\_t001 |  |  |  |  |  |  |  |  |  |  |  |  |
| 0 | Vitvi18g01405\_t001 |  |  |  |  |  |  |  |  |  |  |  |  |
| 0 | Vitvi18g01404\_t001 |  |  |  |  |  |  |  |  |  |  |  |  |
| 0 | Vitvi18g04334\_t001 |  |  |  |  |  |  |  |  |  |  |  |  |
| 0 | Vitvi18g02872\_t001 |  |  |  |  |  |  |  |  |  |  |  |  |
| 0 | Vitvi18g04335\_t001 |  |  |  |  |  |  |  |  |  |  |  |  |
| 0 | Vitvi18g01401\_t001 |  |  |  |  |  |  |  |  |  |  |  |  |
| 0 | Vitvi18g04336\_t001 |  |  |  |  |  |  |  |  |  |  |  |  |
| 0 | Vitvi18g02870\_t001 |  |  |  |  |  |  |  |  |  |  |  |  |
| 0 | Vitvi18g01397\_t001 |  |  |  |  |  |  |  |  |  |  |  |  |
| 0 | Vitvi18g01395\_t001 |  |  |  |  |  |  |  |  |  |  |  |  |
| 0 | Vitvi18g02869\_t001 |  |  |  |  |  |  |  |  |  |  |  |  |
| 0 | Vitvi18g04337\_t001 |  |  |  |  |  |  |  |  |  |  |  |  |
| 0 | Vitvi18g01393\_t001 |  |  |  |  |  |  |  |  |  |  |  |  |
| 0 | Vitvi18g01392\_t001 |  |  |  |  |  |  |  |  |  |  |  |  |
| 0 | Vitvi18g01391\_t001 |  |  |  |  |  |  |  |  |  |  |  |  |
| 0 | Vitvi18g01390\_t001 |  |  |  |  |  |  |  |  |  |  |  |  |
| 0 | Vitvi18g04338\_t001 |  |  |  |  |  |  |  |  |  |  |  |  |
| 0 | Vitvi18g04339\_t001 |  |  |  |  |  |  |  |  |  |  |  |  |
| 0 | Vitvi18g01385\_t001 |  |  |  |  |  |  |  |  |  |  |  |  |
| 0 | Vitvi18g01384\_t001 |  |  |  |  |  |  |  |  |  |  |  |  |
| 0 | Vitvi18g01383\_t001 |  |  |  |  |  |  |  |  |  |  |  |  |
| 0 | Vitvi18g01381\_t001 |  |  |  |  |  |  |  |  |  |  |  |  |
| 0 | Vitvi18g01380\_t001 |  |  |  |  |  |  |  |  |  |  |  |  |
| 0 | Vitvi18g01376\_t001 |  |  |  |  |  |  |  |  |  |  |  |  |
| 0 | Vitvi18g04341\_t001 |  |  |  |  |  |  |  |  |  |  |  |  |
| 0 | Vitvi18g04342\_t001 |  |  |  |  |  |  |  |  |  |  |  |  |
| 0 | Vitvi18g04343\_t001 |  |  |  |  |  |  |  |  |  |  |  |  |
| 0 | Vitvi18g04344\_t001 |  |  |  |  |  |  |  |  |  |  |  |  |
| 0 | Vitvi18g04345\_t001 |  |  |  |  |  |  |  |  |  |  |  |  |
| 0 | Vitvi18g04346\_t001 |  |  |  |  |  |  |  |  |  |  |  |  |
| 0 | Vitvi18g04347\_t001 |  |  |  |  |  |  |  |  |  |  |  |  |
| 0 | Vitvi18g04348\_t001 |  |  |  |  |  |  |  |  |  |  |  |  |
| 0 | Vitvi18g04349\_t001 |  |  |  |  |  |  |  |  |  |  |  |  |
| 0 | Vitvi18g01436\_t001 |  |  |  |  |  |  |  |  |  |  |  |  |
| 0 | Vitvi18g01435\_t001 |  |  |  |  |  |  |  |  |  |  |  |  |
| 0 | Vitvi18g04350\_t001 |  |  |  |  |  |  |  |  |  |  |  |  |
| 0 | Vitvi18g01433\_t001 |  |  |  |  |  |  |  |  |  |  |  |  |
| 0 | Vitvi18g01432\_t001 |  |  |  |  |  |  |  |  |  |  |  |  |
| 0 | Vitvi18g01431\_t001 |  |  |  |  |  |  |  |  |  |  |  |  |
| 0 | Vitvi18g01430\_t001 |  |  |  |  |  |  |  |  |  |  |  |  |
| 0 | Vitvi18g01429\_t001 |  |  |  |  |  |  |  |  |  |  |  |  |
| 0 | Vitvi18g04351\_t001 |  |  |  |  |  |  |  |  |  |  |  |  |
| 0 | Vitvi18g04352\_t001 |  |  |  |  |  |  |  |  |  |  |  |  |
| 0 | Vitvi18g04353\_t001 |  |  |  |  |  |  |  |  |  |  |  |  |
| 0 | Vitvi18g04354\_t001 |  |  |  |  |  |  |  |  |  |  |  |  |
| 0 | Vitvi18g04355\_t001 |  |  |  |  |  |  |  |  |  |  |  |  |
| 0 | Vitvi18g04356\_t001 |  |  |  |  |  |  |  |  |  |  |  |  |
| 0 | Vitvi18g04357\_t001 |  |  |  |  |  |  |  |  |  |  |  |  |
| 0 | Vitvi18g04358\_t001 |  |  |  |  |  |  |  |  |  |  |  |  |
| 0 | Vitvi18g04359\_t001 |  |  |  |  |  |  |  |  |  |  |  |  |
| 0 | Vitvi18g01488\_t001 |  |  |  |  |  |  |  |  |  |  |  |  |
| 0 | Vitvi18g04360\_t001 |  |  |  |  |  |  |  |  |  |  |  |  |
| 0 | Vitvi18g04361\_t001 |  |  |  |  |  |  |  |  |  |  |  |  |
| 0 | Vitvi18g04362\_t001 |  |  |  |  |  |  |  |  |  |  |  |  |
| 0 | Vitvi18g04363\_t001 |  |  |  |  |  |  |  |  |  |  |  |  |
| 0 | Vitvi18g04364\_t001 |  |  |  |  |  |  |  |  |  |  |  |  |
| 0 | Vitvi18g04365\_t001 |  |  |  |  |  |  |  |  |  |  |  |  |
| 0 | Vitvi18g04366\_t001 |  |  |  |  |  |  |  |  |  |  |  |  |
| 0 | Vitvi18g04367\_t001 |  |  |  |  |  |  |  |  |  |  |  |  |
| 0 | Vitvi18g04368\_t001 |  |  |  |  |  |  |  |  |  |  |  |  |
| 0 | Vitvi18g04369\_t001 |  |  |  |  |  |  |  |  |  |  |  |  |
| 0 | Vitvi18g02909\_t001 |  |  |  |  |  |  |  |  |  |  |  |  |
| 0 | Vitvi18g04370\_t001 |  |  |  |  |  |  |  |  |  |  |  |  |
| 0 | Vitvi18g04371\_t001 |  |  |  |  |  |  |  |  |  |  |  |  |
| 0 | Vitvi18g04372\_t001 |  |  |  |  |  |  |  |  |  |  |  |  |
| 0 | Vitvi18g04373\_t001 |  |  |  |  |  |  |  |  |  |  |  |  |
| 0 | Vitvi18g04374\_t001 |  |  |  |  |  |  |  |  |  |  |  |  |
| 0 | Vitvi18g04375\_t001 |  |  |  |  |  |  |  |  |  |  |  |  |
| 0 | Vitvi18g04376\_t001 |  |  |  |  |  |  |  |  |  |  |  |  |
| 0 | Vitvi18g04377\_t001 |  |  |  |  |  |  |  |  |  |  |  |  |
| 0 | Vitvi18g01452\_t001 |  |  |  |  |  |  |  |  |  |  |  |  |
| 0 | Vitvi18g04378\_t001 |  |  |  |  |  |  |  |  |  |  |  |  |
| 1 | Vitvi18g04379\_t001 |  | Vitvi18g04400\_t001 |  |  |  |  |  |  |  |  |  |  |
| 1 | Vitvi18g01450\_t001 |  | \| \| |  |  |  |  |  |  |  |  |  |  |
| 1 | Vitvi18g03352\_t001 |  | Vitvi18g01598\_t001 |  |  |  |  |  |  |  |  |  |  |
| 1 | Vitvi18g04380\_t001 |  | Vitvi18g04396\_t001 |  |  |  |  |  |  |  |  |  |  |
| 1 | Vitvi18g04381\_t001 |  | \| \| |  |  |  |  |  |  |  |  |  |  |
| 1 | Vitvi18g03350\_t001 |  | Vitvi18g02955\_t001 |  |  |  |  |  |  |  |  |  |  |
| 1 | Vitvi18g04382\_t001 |  | Vitvi18g01596\_t001 |  |  |  |  |  |  |  |  |  |  |
| 1 | Vitvi18g02938\_t001 |  | Vitvi18g02951\_t001 |  |  |  |  |  |  |  |  |  |  |
| 0 | Vitvi18g01577\_t001 |  |  |  |  |  |  |  |  |  |  |  |  |
| 0 | Vitvi18g04383\_t001 |  |  |  |  |  |  |  |  |  |  |  |  |
| 0 | Vitvi18g04384\_t001 |  |  |  |  |  |  |  |  |  |  |  |  |
| 0 | Vitvi18g01579\_t001 |  |  |  |  |  |  |  |  |  |  |  |  |
| 0 | Vitvi18g04385\_t001 |  |  |  |  |  |  |  |  |  |  |  |  |
| 0 | Vitvi18g04386\_t001 |  |  |  |  |  |  |  |  |  |  |  |  |
| 0 | Vitvi18g02939\_t001 |  |  |  |  |  |  |  |  |  |  |  |  |
| 0 | Vitvi18g04387\_t001 |  |  |  |  |  |  |  |  |  |  |  |  |
| 0 | Vitvi18g01580\_t001 |  |  |  |  |  |  |  |  |  |  |  |  |
| 0 | Vitvi18g04388\_t001 |  |  |  |  |  |  |  |  |  |  |  |  |
| 0 | Vitvi18g04389\_t001 |  |  |  |  |  |  |  |  |  |  |  |  |
| 0 | Vitvi18g01587\_t001 |  |  |  |  |  |  |  |  |  |  |  |  |
| 0 | Vitvi18g02948\_t001 |  |  |  |  |  |  |  |  |  |  |  |  |
| 0 | Vitvi18g04390\_t001 |  |  |  |  |  |  |  |  |  |  |  |  |
| 0 | Vitvi18g04391\_t001 |  |  |  |  |  |  |  |  |  |  |  |  |
| 0 | Vitvi18g02950\_t001 |  |  |  |  |  |  |  |  |  |  |  |  |
| 0 | Vitvi18g04392\_t001 |  |  |  |  |  |  |  |  |  |  |  |  |
| 1 | Vitvi18g02951\_t001 |  | Vitvi18g02938\_t001 |  |  |  |  |  |  |  |  |  |  |
| 1 | Vitvi18g04393\_t001 |  | \| \| |  |  |  |  |  |  |  |  |  |  |
| 1 | Vitvi18g01596\_t001 |  | Vitvi18g04382\_t001 |  |  |  |  |  |  |  |  |  |  |
| 1 | Vitvi18g04394\_t001 |  | \| \| |  |  |  |  |  |  |  |  |  |  |
| 1 | Vitvi18g04395\_t001 |  | \| \| |  |  |  |  |  |  |  |  |  |  |
| 1 | Vitvi18g02955\_t001 |  | Vitvi18g03350\_t001 |  |  |  |  |  |  |  |  |  |  |
| 1 | Vitvi18g04396\_t001 |  | Vitvi18g04380\_t001 |  |  |  |  |  |  |  |  |  |  |
| 1 | Vitvi18g01598\_t001 |  | Vitvi18g03352\_t001 |  |  |  |  |  |  |  |  |  |  |
| 1 | Vitvi18g04397\_t001 |  | \| \| |  |  |  |  |  |  |  |  |  |  |
| 1 | Vitvi18g04398\_t001 |  | \| \| |  |  |  |  |  |  |  |  |  |  |
| 1 | Vitvi18g04399\_t001 |  | \| \| |  |  |  |  |  |  |  |  |  |  |
| 1 | Vitvi18g04400\_t001 |  | Vitvi18g04379\_t001 |  |  |  |  |  |  |  |  |  |  |
| 0 | Vitvi18g01501\_t001 |  |  |  |  |  |  |  |  |  |  |  |  |
| 0 | Vitvi18g01503\_t001 |  |  |  |  |  |  |  |  |  |  |  |  |
| 0 | Vitvi18g01504\_t001 |  |  |  |  |  |  |  |  |  |  |  |  |
| 0 | Vitvi18g01507\_t001 |  |  |  |  |  |  |  |  |  |  |  |  |
| 0 | Vitvi18g04401\_t001 |  |  |  |  |  |  |  |  |  |  |  |  |
| 0 | Vitvi18g01508\_t001 |  |  |  |  |  |  |  |  |  |  |  |  |
| 0 | Vitvi18g01510\_t001 |  |  |  |  |  |  |  |  |  |  |  |  |
| 0 | Vitvi18g01511\_t001 |  |  |  |  |  |  |  |  |  |  |  |  |
| 0 | Vitvi18g01512\_t001 |  |  |  |  |  |  |  |  |  |  |  |  |
| 0 | Vitvi18g04402\_t001 |  |  |  |  |  |  |  |  |  |  |  |  |
| 1 | Vitvi18g01517\_t001 |  | Vitvi07g01835\_t001 |  |  |  |  |  |  |  |  |  |  |
| 1 | Vitvi18g01518\_t001 |  | \| \| |  |  |  |  |  |  |  |  |  |  |
| 1 | Vitvi18g01520\_t001 |  | \| \| |  |  |  |  |  |  |  |  |  |  |
| 1 | Vitvi18g01522\_t002 |  | \| \| |  |  |  |  |  |  |  |  |  |  |
| 1 | Vitvi18g01524\_t001 |  | \| \| |  |  |  |  |  |  |  |  |  |  |
| 1 | Vitvi18g04403\_t001 |  | \| \| |  |  |  |  |  |  |  |  |  |  |
| 1 | Vitvi18g01526\_t001 |  | \| \| |  |  |  |  |  |  |  |  |  |  |
| 1 | Vitvi18g01527\_t001 |  | \| \| |  |  |  |  |  |  |  |  |  |  |
| 1 | Vitvi18g02917\_t001 |  | \| \| |  |  |  |  |  |  |  |  |  |  |
| 1 | Vitvi18g01528\_t001 |  | \| \| |  |  |  |  |  |  |  |  |  |  |
| 1 | Vitvi18g02918\_t001 |  | \| \| |  |  |  |  |  |  |  |  |  |  |
| 1 | Vitvi18g02920\_t001 |  | \| \| |  |  |  |  |  |  |  |  |  |  |
| 1 | Vitvi18g01531\_t001 |  | Vitvi07g01846\_t001 |  |  |  |  |  |  |  |  |  |  |
| 1 | Vitvi18g01533\_t001 |  | \| \| |  |  |  |  |  |  |  |  |  |  |
| 1 | Vitvi18g04404\_t001 |  | \| \| |  |  |  |  |  |  |  |  |  |  |
| 1 | Vitvi18g01535\_t001 |  | Vitvi07g01848\_t001 |  |  |  |  |  |  |  |  |  |  |
| 1 | Vitvi18g02921\_t001 |  | \| \| |  |  |  |  |  |  |  |  |  |  |
| 1 | Vitvi18g02924\_t001 |  | \| \| |  |  |  |  |  |  |  |  |  |  |
| 1 | Vitvi18g02922\_t001 |  | \| \| |  |  |  |  |  |  |  |  |  |  |
| 1 | Vitvi18g04405\_t001 |  | \| \| |  |  |  |  |  |  |  |  |  |  |
| 1 | Vitvi18g02927\_t001 |  | \| \| |  |  |  |  |  |  |  |  |  |  |
| 1 | Vitvi18g02928\_t001 |  | \| \| |  |  |  |  |  |  |  |  |  |  |
| 1 | Vitvi18g04406\_t001 |  | \| \| |  |  |  |  |  |  |  |  |  |  |
| 1 | Vitvi18g01539\_t003 |  | \| \| |  |  |  |  |  |  |  |  |  |  |
| 1 | Vitvi18g04407\_t001 |  | \| \| |  |  |  |  |  |  |  |  |  |  |
| 1 | Vitvi18g01541\_t001 |  | \| \| |  |  |  |  |  |  |  |  |  |  |
| 1 | Vitvi18g01542\_t001 |  | \| \| |  |  |  |  |  |  |  |  |  |  |
| 1 | Vitvi18g01543\_t001 |  | \| \| |  |  |  |  |  |  |  |  |  |  |
| 1 | Vitvi18g02931\_t001 |  | \| \| |  |  |  |  |  |  |  |  |  |  |
| 1 | Vitvi18g01544\_t001 |  | \| \| |  |  |  |  |  |  |  |  |  |  |
| 1 | Vitvi18g01545\_t001 |  | \| \| |  |  |  |  |  |  |  |  |  |  |
| 1 | Vitvi18g01546\_t001 |  | \| \| |  |  |  |  |  |  |  |  |  |  |
| 1 | Vitvi18g01548\_t001 |  | \| \| |  |  |  |  |  |  |  |  |  |  |
| 1 | Vitvi18g01550\_t001 |  | \| \| |  |  |  |  |  |  |  |  |  |  |
| 1 | Vitvi18g04408\_t001 |  | \| \| |  |  |  |  |  |  |  |  |  |  |
| 1 | Vitvi18g01574\_t005 |  | \| \| |  |  |  |  |  |  |  |  |  |  |
| 1 | Vitvi18g01573\_t001 |  | \| \| |  |  |  |  |  |  |  |  |  |  |
| 1 | Vitvi18g01572\_t001.1.6037826d |  | \| \| |  |  |  |  |  |  |  |  |  |  |
| 1 | Vitvi18g02934\_t001 |  | \| \| |  |  |  |  |  |  |  |  |  |  |
| 1 | Vitvi18g01571\_t001 |  | Vitvi07g01857\_t001 |  |  |  |  |  |  |  |  |  |  |
| 1 | Vitvi18g01570\_t001 |  | \| \| |  |  |  |  |  |  |  |  |  |  |
| 1 | Vitvi18g02933\_t001 |  | \| \| |  |  |  |  |  |  |  |  |  |  |
| 1 | Vitvi18g01567\_t001 |  | \| \| |  |  |  |  |  |  |  |  |  |  |
| 1 | Vitvi18g01566\_t001 |  | \| \| |  |  |  |  |  |  |  |  |  |  |
| 1 | Vitvi18g01564\_t001 |  | \| \| |  |  |  |  |  |  |  |  |  |  |
| 1 | Vitvi18g01563\_t001 |  | \| \| |  |  |  |  |  |  |  |  |  |  |
| 1 | Vitvi18g01562\_t001 |  | \| \| |  |  |  |  |  |  |  |  |  |  |
| 1 | Vitvi18g01561\_t001 |  | \| \| |  |  |  |  |  |  |  |  |  |  |
| 1 | Vitvi18g01558\_t001 |  | \| \| |  |  |  |  |  |  |  |  |  |  |
| 1 | Vitvi18g01557\_t001 |  | \| \| |  |  |  |  |  |  |  |  |  |  |
| 1 | Vitvi18g04409\_t001 |  | \| \| |  |  |  |  |  |  |  |  |  |  |
| 1 | Vitvi18g01555\_t001 |  | \| \| |  |  |  |  |  |  |  |  |  |  |
| 1 | Vitvi18g04410\_t001 |  | \| \| |  |  |  |  |  |  |  |  |  |  |
| 1 | Vitvi18g04411\_t001 |  | \| \| |  |  |  |  |  |  |  |  |  |  |
| 1 | Vitvi18g01553\_t001 |  | \| \| |  |  |  |  |  |  |  |  |  |  |
| 1 | Vitvi18g01552\_t001 |  | \| \| |  |  |  |  |  |  |  |  |  |  |
| 1 | Vitvi18g04412\_t001 |  | \| \| |  |  |  |  |  |  |  |  |  |  |
| 1 | Vitvi18g04413\_t001 |  | \| \| |  |  |  |  |  |  |  |  |  |  |
| 1 | Vitvi18g04414\_t001 |  | \| \| |  |  |  |  |  |  |  |  |  |  |
| 1 | Vitvi18g01602\_t001 |  | Vitvi07g01862\_t001 |  |  |  |  |  |  |  |  |  |  |
| 1 | Vitvi18g04415\_t001 |  | \| \| |  |  |  |  |  |  |  |  |  |  |
| 1 | Vitvi18g01603\_t001 |  | \| \| |  |  |  |  |  |  |  |  |  |  |
| 1 | Vitvi18g01604\_t001 |  | \| \| |  |  |  |  |  |  |  |  |  |  |
| 1 | Vitvi18g02961\_t001 |  | \| \| |  |  |  |  |  |  |  |  |  |  |
| 1 | Vitvi18g01605\_t001 |  | \| \| |  |  |  |  |  |  |  |  |  |  |
| 1 | Vitvi18g01607\_t001 |  | \| \| |  |  |  |  |  |  |  |  |  |  |
| 1 | Vitvi18g04416\_t001 |  | Vitvi07g04740\_t001 |  |  |  |  |  |  |  |  |  |  |
| 1 | Vitvi18g04417\_t001 |  | \| \| |  |  |  |  |  |  |  |  |  |  |
| 1 | Vitvi18g01608\_t001 |  | \| \| |  |  |  |  |  |  |  |  |  |  |
| 1 | Vitvi18g01610\_t001 |  | \| \| |  |  |  |  |  |  |  |  |  |  |
| 1 | Vitvi18g02963\_t001 |  | \| \| |  |  |  |  |  |  |  |  |  |  |
| 1 | Vitvi18g02964\_t001 |  | \| \| |  |  |  |  |  |  |  |  |  |  |
| 1 | Vitvi18g02965\_t001 |  | \| \| |  |  |  |  |  |  |  |  |  |  |
| 1 | Vitvi18g04418\_t001 |  | \| \| |  |  |  |  |  |  |  |  |  |  |
| 1 | Vitvi18g01614\_t001 |  | Vitvi07g01871\_t001 |  |  |  |  |  |  |  |  |  |  |
| 1 | Vitvi18g02966\_t001 |  | \| \| |  |  |  |  |  |  |  |  |  |  |
| 1 | Vitvi18g02967\_t001 |  | \| \| |  |  |  |  |  |  |  |  |  |  |
| 1 | Vitvi18g02968\_t001 |  | \| \| |  |  |  |  |  |  |  |  |  |  |
| 1 | Vitvi18g04419\_t001 |  | \| \| |  |  |  |  |  |  |  |  |  |  |
| 1 | Vitvi18g04420\_t001 |  | \| \| |  |  |  |  |  |  |  |  |  |  |
| 1 | Vitvi18g04421\_t001 |  | \| \| |  |  |  |  |  |  |  |  |  |  |
| 1 | Vitvi18g01617\_t001 |  | Vitvi07g01874\_t001 |  |  |  |  |  |  |  |  |  |  |
| 1 | Vitvi18g04422\_t001 |  | \| \| |  |  |  |  |  |  |  |  |  |  |
| 1 | Vitvi18g01618\_t001 |  | \| \| |  |  |  |  |  |  |  |  |  |  |
| 1 | Vitvi18g04423\_t001 |  | \| \| |  |  |  |  |  |  |  |  |  |  |
| 1 | Vitvi18g01620\_t001 |  | \| \| |  |  |  |  |  |  |  |  |  |  |
| 1 | Vitvi18g01621\_t001 |  | Vitvi07g01878\_t001 |  |  |  |  |  |  |  |  |  |  |
| 1 | Vitvi18g01623\_t001 |  | \| \| |  |  |  |  |  |  |  |  |  |  |
| 1 | Vitvi18g01624\_t001 |  | Vitvi07g01879\_t001 |  |  |  |  |  |  |  |  |  |  |
| 1 | Vitvi18g01625\_t001 |  | Vitvi07g01880\_t001 |  |  |  |  |  |  |  |  |  |  |
| 1 | Vitvi18g04424\_t001 |  | \| \| |  |  |  |  |  |  |  |  |  |  |
| 1 | Vitvi18g02971\_t002 |  | \| \| |  |  |  |  |  |  |  |  |  |  |
| 1 | Vitvi18g01628\_t001 |  | Vitvi07g01881\_t001 |  |  |  |  |  |  |  |  |  |  |
| 2 | Vitvi18g02972\_t001 |  | Vitvi07g01882\_t001 |  | Vitvi04g01581\_t001 |  |  |  |  |  |  |  |  |
| 2 | Vitvi18g02973\_t001 |  | \| \| |  | \| \| |  |  |  |  |  |  |  |  |
| 2 | Vitvi18g01629\_t001 |  | \| \| |  | \| \| |  |  |  |  |  |  |  |  |
| 2 | Vitvi18g02974\_t001 |  | \| \| |  | \| \| |  |  |  |  |  |  |  |  |
| 2 | Vitvi18g02975\_t001 |  | \| \| |  | \| \| |  |  |  |  |  |  |  |  |
| 2 | Vitvi18g01631\_t001 |  | Vitvi07g01883\_t001 |  | Vitvi04g01583\_t001 |  |  |  |  |  |  |  |  |
| 1 | Vitvi18g01632\_t003 |  |  |  | \| \| |  |  |  |  |  |  |  |  |
| 1 | Vitvi18g02977\_t001 |  |  |  | \| \| |  |  |  |  |  |  |  |  |
| 1 | Vitvi18g01633\_t001 |  |  |  | \| \| |  |  |  |  |  |  |  |  |
| 1 | Vitvi18g01634\_t002 |  |  |  | Vitvi04g01591\_t005 |  |  |  |  |  |  |  |  |
| 1 | Vitvi18g02978\_t001 |  |  |  | \| \| |  |  |  |  |  |  |  |  |
| 1 | Vitvi18g01636\_t001 |  |  |  | Vitvi04g02211\_t001 |  |  |  |  |  |  |  |  |
| 1 | Vitvi18g01637\_t001 |  |  |  | \| \| |  |  |  |  |  |  |  |  |
| 1 | Vitvi18g02979\_t001 |  |  |  | \| \| |  |  |  |  |  |  |  |  |
| 1 | Vitvi18g02980\_t001 |  |  |  | \| \| |  |  |  |  |  |  |  |  |
| 1 | Vitvi18g02981\_t001 |  |  |  | \| \| |  |  |  |  |  |  |  |  |
| 1 | Vitvi18g01639\_t001 |  |  |  | \| \| |  |  |  |  |  |  |  |  |
| 1 | Vitvi18g01640\_t001 |  |  |  | \| \| |  |  |  |  |  |  |  |  |
| 1 | Vitvi18g01641\_t001 |  |  |  | \| \| |  |  |  |  |  |  |  |  |
| 1 | Vitvi18g01643\_t001 |  |  |  | \| \| |  |  |  |  |  |  |  |  |
| 1 | Vitvi18g01647\_t004 |  |  |  | \| \| |  |  |  |  |  |  |  |  |
| 1 | Vitvi18g01648\_t001 |  |  |  | Vitvi04g01596\_t001 |  |  |  |  |  |  |  |  |
| 1 | Vitvi18g01649\_t001 |  |  |  | Vitvi04g01604\_t001 |  |  |  |  |  |  |  |  |
| 1 | Vitvi18g04425\_t001 |  |  |  | \| \| |  |  |  |  |  |  |  |  |
| 1 | Vitvi18g01654\_t001 |  |  |  | \| \| |  |  |  |  |  |  |  |  |
| 1 | Vitvi18g01655\_t001 |  |  |  | \| \| |  |  |  |  |  |  |  |  |
| 1 | Vitvi18g04426\_t001 |  |  |  | \| \| |  |  |  |  |  |  |  |  |
| 1 | Vitvi18g01657\_t001 |  |  |  | \| \| |  |  |  |  |  |  |  |  |
| 1 | Vitvi18g01659\_t001 |  |  |  | \| \| |  |  |  |  |  |  |  |  |
| 1 | Vitvi18g02990\_t003 |  |  |  | \| \| |  |  |  |  |  |  |  |  |
| 1 | Vitvi18g04427\_t001 |  |  |  | Vitvi04g04474\_t001 |  |  |  |  |  |  |  |  |
| 0 | Vitvi18g04428\_t001 |  |  |  |  |  |  |  |  |  |  |  |  |
| 0 | Vitvi18g02993\_t001 |  |  |  |  |  |  |  |  |  |  |  |  |
| 0 | Vitvi18g01661\_t001 |  |  |  |  |  |  |  |  |  |  |  |  |
| 0 | Vitvi18g01662\_t003 |  |  |  |  |  |  |  |  |  |  |  |  |
| 0 | Vitvi18g04429\_t001 |  |  |  |  |  |  |  |  |  |  |  |  |
| 0 | Vitvi18g01664\_t001 |  |  |  |  |  |  |  |  |  |  |  |  |
| 0 | Vitvi18g01665\_t001 |  |  |  |  |  |  |  |  |  |  |  |  |
| 0 | Vitvi18g01666\_t001 |  |  |  |  |  |  |  |  |  |  |  |  |
| 0 | Vitvi18g01667\_t002 |  |  |  |  |  |  |  |  |  |  |  |  |
| 0 | Vitvi18g01669\_t001 |  |  |  |  |  |  |  |  |  |  |  |  |
| 0 | Vitvi18g04430\_t001 |  |  |  |  |  |  |  |  |  |  |  |  |
| 0 | Vitvi18g01670\_t002 |  |  |  |  |  |  |  |  |  |  |  |  |
| 0 | Vitvi18g02995\_t001 |  |  |  |  |  |  |  |  |  |  |  |  |
| 0 | Vitvi18g01671\_t003 |  |  |  |  |  |  |  |  |  |  |  |  |
| 0 | Vitvi18g01674\_t001 |  |  |  |  |  |  |  |  |  |  |  |  |
| 0 | Vitvi18g01675\_t001 |  |  |  |  |  |  |  |  |  |  |  |  |
| 0 | Vitvi18g01676\_t001 |  |  |  |  |  |  |  |  |  |  |  |  |
| 0 | Vitvi18g01677\_t001 |  |  |  |  |  |  |  |  |  |  |  |  |
| 0 | Vitvi18g01678\_t001 |  |  |  |  |  |  |  |  |  |  |  |  |
| 0 | Vitvi18g01679\_t001 |  |  |  |  |  |  |  |  |  |  |  |  |
| 0 | Vitvi18g04431\_t001 |  |  |  |  |  |  |  |  |  |  |  |  |
| 0 | Vitvi18g01681\_t001 |  |  |  |  |  |  |  |  |  |  |  |  |
| 0 | Vitvi18g01682\_t001 |  |  |  |  |  |  |  |  |  |  |  |  |
| 0 | Vitvi18g01683\_t001 |  |  |  |  |  |  |  |  |  |  |  |  |
| 0 | Vitvi18g04432\_t001 |  |  |  |  |  |  |  |  |  |  |  |  |
| 0 | Vitvi18g01684\_t001 |  |  |  |  |  |  |  |  |  |  |  |  |
| 0 | Vitvi18g01686\_t001 |  |  |  |  |  |  |  |  |  |  |  |  |
| 0 | Vitvi18g01687\_t001 |  |  |  |  |  |  |  |  |  |  |  |  |
| 0 | Vitvi18g02996\_t001 |  |  |  |  |  |  |  |  |  |  |  |  |
| 0 | Vitvi18g04433\_t001 |  |  |  |  |  |  |  |  |  |  |  |  |
| 0 | Vitvi18g04434\_t001 |  |  |  |  |  |  |  |  |  |  |  |  |
| 0 | Vitvi18g01690\_t001 |  |  |  |  |  |  |  |  |  |  |  |  |
| 0 | Vitvi18g01691\_t001 |  |  |  |  |  |  |  |  |  |  |  |  |
| 0 | Vitvi18g02997\_t001 |  |  |  |  |  |  |  |  |  |  |  |  |
| 0 | Vitvi18g01692\_t002 |  |  |  |  |  |  |  |  |  |  |  |  |
| 0 | Vitvi18g01694\_t001 |  |  |  |  |  |  |  |  |  |  |  |  |
| 0 | Vitvi18g04435\_t001 |  |  |  |  |  |  |  |  |  |  |  |  |
| 0 | Vitvi18g01696\_t001 |  |  |  |  |  |  |  |  |  |  |  |  |
| 0 | Vitvi18g01699\_t001 |  |  |  |  |  |  |  |  |  |  |  |  |
| 0 | Vitvi18g01700\_t001 |  |  |  |  |  |  |  |  |  |  |  |  |
| 0 | Vitvi18g01701\_t001 |  |  |  |  |  |  |  |  |  |  |  |  |
| 0 | Vitvi18g01703\_t001 |  |  |  |  |  |  |  |  |  |  |  |  |
| 0 | Vitvi18g04437\_t001 |  |  |  |  |  |  |  |  |  |  |  |  |
| 0 | Vitvi18g01706\_t001 |  |  |  |  |  |  |  |  |  |  |  |  |
| 0 | Vitvi18g01707\_t001 |  |  |  |  |  |  |  |  |  |  |  |  |
| 0 | Vitvi18g01708\_t001 |  |  |  |  |  |  |  |  |  |  |  |  |
| 0 | Vitvi18g01709\_t001 |  |  |  |  |  |  |  |  |  |  |  |  |
| 0 | Vitvi18g01711\_t001 |  |  |  |  |  |  |  |  |  |  |  |  |
| 0 | Vitvi18g01713\_t001 |  |  |  |  |  |  |  |  |  |  |  |  |
| 0 | Vitvi18g04438\_t001 |  |  |  |  |  |  |  |  |  |  |  |  |
| 0 | Vitvi18g04439\_t001 |  |  |  |  |  |  |  |  |  |  |  |  |
| 0 | Vitvi18g04440\_t001 |  |  |  |  |  |  |  |  |  |  |  |  |
| 0 | Vitvi18g04441\_t001 |  |  |  |  |  |  |  |  |  |  |  |  |
| 0 | Vitvi18g04442\_t001 |  |  |  |  |  |  |  |  |  |  |  |  |
| 0 | Vitvi18g03004\_t001 |  |  |  |  |  |  |  |  |  |  |  |  |
| 0 | Vitvi18g03005\_t001 |  |  |  |  |  |  |  |  |  |  |  |  |
| 0 | Vitvi18g03006\_t001 |  |  |  |  |  |  |  |  |  |  |  |  |
| 0 | Vitvi18g04443\_t001 |  |  |  |  |  |  |  |  |  |  |  |  |
| 0 | Vitvi18g01719\_t001 |  |  |  |  |  |  |  |  |  |  |  |  |
| 0 | Vitvi18g01721\_t001 |  |  |  |  |  |  |  |  |  |  |  |  |
| 0 | Vitvi18g01725\_t001 |  |  |  |  |  |  |  |  |  |  |  |  |
| 0 | Vitvi18g01726\_t001 |  |  |  |  |  |  |  |  |  |  |  |  |
| 0 | Vitvi18g01729\_t001 |  |  |  |  |  |  |  |  |  |  |  |  |
| 0 | Vitvi18g04444\_t001 |  |  |  |  |  |  |  |  |  |  |  |  |
| 0 | Vitvi18g04445\_t001 |  |  |  |  |  |  |  |  |  |  |  |  |
| 0 | Vitvi18g01735\_t001 |  |  |  |  |  |  |  |  |  |  |  |  |
| 0 | Vitvi18g01736\_t001 |  |  |  |  |  |  |  |  |  |  |  |  |
| 0 | Vitvi18g03009\_t001 |  |  |  |  |  |  |  |  |  |  |  |  |
| 0 | Vitvi18g03010\_t001 |  |  |  |  |  |  |  |  |  |  |  |  |
| 0 | Vitvi18g04446\_t001 |  |  |  |  |  |  |  |  |  |  |  |  |
| 0 | Vitvi18g01739\_t001 |  |  |  |  |  |  |  |  |  |  |  |  |
| 0 | Vitvi18g04447\_t001 |  |  |  |  |  |  |  |  |  |  |  |  |
| 0 | Vitvi18g01743\_t001 |  |  |  |  |  |  |  |  |  |  |  |  |
| 0 | Vitvi18g01744\_t001 |  |  |  |  |  |  |  |  |  |  |  |  |
| 0 | Vitvi18g04448\_t001 |  |  |  |  |  |  |  |  |  |  |  |  |
| 0 | Vitvi18g01745\_t001 |  |  |  |  |  |  |  |  |  |  |  |  |
| 0 | Vitvi18g03014\_t001 |  |  |  |  |  |  |  |  |  |  |  |  |
| 0 | Vitvi18g03015\_t001 |  |  |  |  |  |  |  |  |  |  |  |  |
| 0 | Vitvi18g01746\_t001 |  |  |  |  |  |  |  |  |  |  |  |  |
| 0 | Vitvi18g03016\_t001 |  |  |  |  |  |  |  |  |  |  |  |  |
| 0 | Vitvi18g04449\_t001 |  |  |  |  |  |  |  |  |  |  |  |  |
| 0 | Vitvi18g01750\_t001 |  |  |  |  |  |  |  |  |  |  |  |  |
| 0 | Vitvi18g01751\_t001 |  |  |  |  |  |  |  |  |  |  |  |  |
| 0 | Vitvi18g04450\_t001 |  |  |  |  |  |  |  |  |  |  |  |  |
| 0 | Vitvi18g04451\_t001 |  |  |  |  |  |  |  |  |  |  |  |  |
| 0 | Vitvi18g01761\_t001 |  |  |  |  |  |  |  |  |  |  |  |  |
| 0 | Vitvi18g04452\_t001 |  |  |  |  |  |  |  |  |  |  |  |  |
| 0 | Vitvi18g04453\_t001 |  |  |  |  |  |  |  |  |  |  |  |  |
| 0 | Vitvi18g01763\_t001 |  |  |  |  |  |  |  |  |  |  |  |  |
| 0 | Vitvi18g04454\_t001 |  |  |  |  |  |  |  |  |  |  |  |  |
| 0 | Vitvi18g04455\_t001 |  |  |  |  |  |  |  |  |  |  |  |  |
| 0 | Vitvi18g04456\_t001 |  |  |  |  |  |  |  |  |  |  |  |  |
| 0 | Vitvi18g01765\_t001 |  |  |  |  |  |  |  |  |  |  |  |  |
| 0 | Vitvi18g04457\_t001 |  |  |  |  |  |  |  |  |  |  |  |  |
| 0 | Vitvi18g01766\_t001 |  |  |  |  |  |  |  |  |  |  |  |  |
| 0 | Vitvi18g01767\_t001 |  |  |  |  |  |  |  |  |  |  |  |  |
| 0 | Vitvi18g04458\_t001 |  |  |  |  |  |  |  |  |  |  |  |  |
| 0 | Vitvi18g01769\_t001 |  |  |  |  |  |  |  |  |  |  |  |  |
| 0 | Vitvi18g04459\_t001 |  |  |  |  |  |  |  |  |  |  |  |  |
| 0 | Vitvi18g04460\_t001 |  |  |  |  |  |  |  |  |  |  |  |  |
| 0 | Vitvi18g04461\_t001 |  |  |  |  |  |  |  |  |  |  |  |  |
| 0 | Vitvi18g04462\_t001 |  |  |  |  |  |  |  |  |  |  |  |  |
| 0 | Vitvi18g04463\_t001 |  |  |  |  |  |  |  |  |  |  |  |  |
| 0 | Vitvi18g04464\_t001 |  |  |  |  |  |  |  |  |  |  |  |  |
| 0 | Vitvi18g04465\_t001 |  |  |  |  |  |  |  |  |  |  |  |  |
| 0 | Vitvi18g04466\_t001 |  |  |  |  |  |  |  |  |  |  |  |  |
| 0 | Vitvi18g04467\_t001 |  |  |  |  |  |  |  |  |  |  |  |  |
| 0 | Vitvi18g01772\_t001 |  |  |  |  |  |  |  |  |  |  |  |  |
| 0 | Vitvi18g01775\_t001 |  |  |  |  |  |  |  |  |  |  |  |  |
| 0 | Vitvi18g04468\_t001 |  |  |  |  |  |  |  |  |  |  |  |  |
| 0 | Vitvi18g04469\_t001 |  |  |  |  |  |  |  |  |  |  |  |  |
| 0 | Vitvi18g04470\_t001 |  |  |  |  |  |  |  |  |  |  |  |  |
| 0 | Vitvi18g04471\_t001 |  |  |  |  |  |  |  |  |  |  |  |  |
| 0 | Vitvi18g01780\_t001 |  |  |  |  |  |  |  |  |  |  |  |  |
| 0 | Vitvi18g01781\_t001 |  |  |  |  |  |  |  |  |  |  |  |  |
| 0 | Vitvi18g04472\_t001 |  |  |  |  |  |  |  |  |  |  |  |  |
| 0 | Vitvi18g01785\_t001 |  |  |  |  |  |  |  |  |  |  |  |  |
| 0 | Vitvi18g01786\_t001 |  |  |  |  |  |  |  |  |  |  |  |  |
| 0 | Vitvi18g01787\_t001 |  |  |  |  |  |  |  |  |  |  |  |  |
| 0 | Vitvi18g01788\_t001 |  |  |  |  |  |  |  |  |  |  |  |  |
| 0 | Vitvi18g01792\_t001 |  |  |  |  |  |  |  |  |  |  |  |  |
| 0 | Vitvi18g04474\_t001 |  |  |  |  |  |  |  |  |  |  |  |  |
| 0 | Vitvi18g04475\_t001 |  |  |  |  |  |  |  |  |  |  |  |  |
| 0 | Vitvi18g01796\_t001 |  |  |  |  |  |  |  |  |  |  |  |  |
| 0 | Vitvi18g01799\_t001 |  |  |  |  |  |  |  |  |  |  |  |  |
| 0 | Vitvi18g03034\_t001 |  |  |  |  |  |  |  |  |  |  |  |  |
| 0 | Vitvi18g04476\_t001 |  |  |  |  |  |  |  |  |  |  |  |  |
| 0 | Vitvi18g01803\_t001 |  |  |  |  |  |  |  |  |  |  |  |  |
| 0 | Vitvi18g01806\_t001 |  |  |  |  |  |  |  |  |  |  |  |  |
| 0 | Vitvi18g01807\_t001 |  |  |  |  |  |  |  |  |  |  |  |  |
| 0 | Vitvi18g01812\_t001 |  |  |  |  |  |  |  |  |  |  |  |  |
| 0 | Vitvi18g04478\_t001 |  |  |  |  |  |  |  |  |  |  |  |  |
| 0 | Vitvi18g04479\_t001 |  |  |  |  |  |  |  |  |  |  |  |  |
| 0 | Vitvi18g01817\_t001 |  |  |  |  |  |  |  |  |  |  |  |  |
| 0 | Vitvi18g04480\_t001 |  |  |  |  |  |  |  |  |  |  |  |  |
| 0 | Vitvi18g04481\_t001 |  |  |  |  |  |  |  |  |  |  |  |  |
| 0 | Vitvi18g01824\_t001 |  |  |  |  |  |  |  |  |  |  |  |  |
| 0 | Vitvi18g01825\_t001 |  |  |  |  |  |  |  |  |  |  |  |  |
| 0 | Vitvi18g01827\_t001 |  |  |  |  |  |  |  |  |  |  |  |  |
| 0 | Vitvi18g04482\_t001 |  |  |  |  |  |  |  |  |  |  |  |  |
| 0 | Vitvi18g01831\_t001 |  |  |  |  |  |  |  |  |  |  |  |  |
| 0 | Vitvi18g04483\_t001 |  |  |  |  |  |  |  |  |  |  |  |  |
| 0 | Vitvi18g04484\_t001 |  |  |  |  |  |  |  |  |  |  |  |  |
| 0 | Vitvi18g01833\_t001 |  |  |  |  |  |  |  |  |  |  |  |  |
| 0 | Vitvi18g01834\_t001 |  |  |  |  |  |  |  |  |  |  |  |  |
| 0 | Vitvi18g01835\_t001 |  |  |  |  |  |  |  |  |  |  |  |  |
| 0 | Vitvi18g04485\_t001 |  |  |  |  |  |  |  |  |  |  |  |  |
| 0 | Vitvi18g04486\_t001 |  |  |  |  |  |  |  |  |  |  |  |  |
| 0 | Vitvi18g04487\_t001 |  |  |  |  |  |  |  |  |  |  |  |  |
| 0 | Vitvi18g01912\_t001 |  |  |  |  |  |  |  |  |  |  |  |  |
| 0 | Vitvi18g01899\_t001 |  |  |  |  |  |  |  |  |  |  |  |  |
| 0 | Vitvi18g04488\_t001 |  |  |  |  |  |  |  |  |  |  |  |  |
| 0 | Vitvi18g04489\_t001 |  |  |  |  |  |  |  |  |  |  |  |  |
| 0 | Vitvi18g04490\_t001 |  |  |  |  |  |  |  |  |  |  |  |  |
| 0 | Vitvi18g04491\_t001 |  |  |  |  |  |  |  |  |  |  |  |  |
| 0 | Vitvi18g04492\_t001 |  |  |  |  |  |  |  |  |  |  |  |  |
| 0 | Vitvi18g04493\_t001 |  |  |  |  |  |  |  |  |  |  |  |  |
| 0 | Vitvi18g03056\_t001 |  |  |  |  |  |  |  |  |  |  |  |  |
| 0 | Vitvi18g04494\_t001 |  |  |  |  |  |  |  |  |  |  |  |  |
| 0 | Vitvi18g04495\_t001 |  |  |  |  |  |  |  |  |  |  |  |  |
| 0 | Vitvi18g01884\_t001 |  |  |  |  |  |  |  |  |  |  |  |  |
| 0 | Vitvi18g04496\_t001 |  |  |  |  |  |  |  |  |  |  |  |  |
| 0 | Vitvi18g04497\_t001 |  |  |  |  |  |  |  |  |  |  |  |  |
| 0 | Vitvi18g04498\_t001 |  |  |  |  |  |  |  |  |  |  |  |  |
| 0 | Vitvi18g01875\_t001 |  |  |  |  |  |  |  |  |  |  |  |  |
| 0 | Vitvi18g01874\_t001 |  |  |  |  |  |  |  |  |  |  |  |  |
| 0 | Vitvi18g03054\_t001 |  |  |  |  |  |  |  |  |  |  |  |  |
| 0 | Vitvi18g04499\_t001 |  |  |  |  |  |  |  |  |  |  |  |  |
| 0 | Vitvi18g01873\_t001 |  |  |  |  |  |  |  |  |  |  |  |  |
| 0 | Vitvi18g01872\_t001 |  |  |  |  |  |  |  |  |  |  |  |  |
| 0 | Vitvi18g03051\_t001 |  |  |  |  |  |  |  |  |  |  |  |  |
| 0 | Vitvi18g04500\_t001 |  |  |  |  |  |  |  |  |  |  |  |  |
| 0 | Vitvi18g01871\_t001 |  |  |  |  |  |  |  |  |  |  |  |  |
| 0 | Vitvi18g01870\_t001 |  |  |  |  |  |  |  |  |  |  |  |  |
| 0 | Vitvi18g01869\_t001 |  |  |  |  |  |  |  |  |  |  |  |  |
| 0 | Vitvi18g01868\_t001 |  |  |  |  |  |  |  |  |  |  |  |  |
| 0 | Vitvi18g01867\_t001 |  |  |  |  |  |  |  |  |  |  |  |  |
| 0 | Vitvi18g01866\_t001 |  |  |  |  |  |  |  |  |  |  |  |  |
| 0 | Vitvi18g01861\_t001 |  |  |  |  |  |  |  |  |  |  |  |  |
| 0 | Vitvi18g03050\_t001 |  |  |  |  |  |  |  |  |  |  |  |  |
| 0 | Vitvi18g01860\_t001 |  |  |  |  |  |  |  |  |  |  |  |  |
| 0 | Vitvi18g01859\_t001 |  |  |  |  |  |  |  |  |  |  |  |  |
| 0 | Vitvi18g04501\_t001 |  |  |  |  |  |  |  |  |  |  |  |  |
| 0 | Vitvi18g04502\_t001 |  |  |  |  |  |  |  |  |  |  |  |  |
| 0 | Vitvi18g04503\_t001 |  |  |  |  |  |  |  |  |  |  |  |  |
| 0 | Vitvi18g03049\_t001 |  |  |  |  |  |  |  |  |  |  |  |  |
| 0 | Vitvi18g01853\_t001 |  |  |  |  |  |  |  |  |  |  |  |  |
| 0 | Vitvi18g04504\_t001 |  |  |  |  |  |  |  |  |  |  |  |  |
| 0 | Vitvi18g03048\_t001 |  |  |  |  |  |  |  |  |  |  |  |  |
| 0 | Vitvi18g01852\_t001 |  |  |  |  |  |  |  |  |  |  |  |  |
| 0 | Vitvi18g03047\_t001 |  |  |  |  |  |  |  |  |  |  |  |  |
| 0 | Vitvi18g03046\_t001 |  |  |  |  |  |  |  |  |  |  |  |  |
| 0 | Vitvi18g03045\_t001 |  |  |  |  |  |  |  |  |  |  |  |  |
| 0 | Vitvi18g01849\_t001 |  |  |  |  |  |  |  |  |  |  |  |  |
| 0 | Vitvi18g04505\_t001 |  |  |  |  |  |  |  |  |  |  |  |  |
| 0 | Vitvi18g04506\_t001 |  |  |  |  |  |  |  |  |  |  |  |  |
| 0 | Vitvi18g04507\_t001 |  |  |  |  |  |  |  |  |  |  |  |  |
| 0 | Vitvi18g04508\_t001 |  |  |  |  |  |  |  |  |  |  |  |  |
| 0 | Vitvi18g03043\_t001 |  |  |  |  |  |  |  |  |  |  |  |  |
| 0 | Vitvi18g04509\_t001 |  |  |  |  |  |  |  |  |  |  |  |  |
| 0 | Vitvi18g03358\_t001 |  |  |  |  |  |  |  |  |  |  |  |  |
| 0 | Vitvi18g04510\_t001 |  |  |  |  |  |  |  |  |  |  |  |  |
| 0 | Vitvi18g03360\_t001 |  |  |  |  |  |  |  |  |  |  |  |  |
| 0 | Vitvi18g04511\_t001 |  |  |  |  |  |  |  |  |  |  |  |  |
| 0 | Vitvi18g03364\_t001 |  |  |  |  |  |  |  |  |  |  |  |  |
| 0 | Vitvi18g03366\_t001 |  |  |  |  |  |  |  |  |  |  |  |  |
| 0 | Vitvi18g04512\_t001 |  |  |  |  |  |  |  |  |  |  |  |  |
| 0 | Vitvi18g04513\_t001 |  |  |  |  |  |  |  |  |  |  |  |  |
| 0 | Vitvi18g04514\_t001 |  |  |  |  |  |  |  |  |  |  |  |  |
| 0 | Vitvi18g04515\_t001 |  |  |  |  |  |  |  |  |  |  |  |  |
| 0 | Vitvi18g03074\_t001 |  |  |  |  |  |  |  |  |  |  |  |  |
| 0 | Vitvi18g03076\_t001 |  |  |  |  |  |  |  |  |  |  |  |  |
| 0 | Vitvi18g04516\_t001 |  |  |  |  |  |  |  |  |  |  |  |  |
| 0 | Vitvi18g04517\_t001 |  |  |  |  |  |  |  |  |  |  |  |  |
| 0 | Vitvi18g04518\_t001 |  |  |  |  |  |  |  |  |  |  |  |  |
| 0 | Vitvi18g01924\_t001 |  |  |  |  |  |  |  |  |  |  |  |  |
| 0 | Vitvi18g04519\_t001 |  |  |  |  |  |  |  |  |  |  |  |  |
| 0 | Vitvi18g04520\_t001 |  |  |  |  |  |  |  |  |  |  |  |  |
| 0 | Vitvi18g01928\_t001 |  |  |  |  |  |  |  |  |  |  |  |  |
| 0 | Vitvi18g04521\_t001 |  |  |  |  |  |  |  |  |  |  |  |  |
| 0 | Vitvi18g04522\_t001 |  |  |  |  |  |  |  |  |  |  |  |  |
| 0 | Vitvi18g04523\_t001 |  |  |  |  |  |  |  |  |  |  |  |  |
| 0 | Vitvi18g04524\_t001 |  |  |  |  |  |  |  |  |  |  |  |  |
| 0 | Vitvi18g04525\_t001 |  |  |  |  |  |  |  |  |  |  |  |  |
| 0 | Vitvi18g04526\_t001 |  |  |  |  |  |  |  |  |  |  |  |  |
| 0 | Vitvi18g04527\_t001 |  |  |  |  |  |  |  |  |  |  |  |  |
| 0 | Vitvi18g04528\_t001 |  |  |  |  |  |  |  |  |  |  |  |  |
| 0 | Vitvi18g04529\_t001 |  |  |  |  |  |  |  |  |  |  |  |  |
| 0 | Vitvi18g04530\_t001 |  |  |  |  |  |  |  |  |  |  |  |  |
| 0 | Vitvi18g04531\_t001 |  |  |  |  |  |  |  |  |  |  |  |  |
| 0 | Vitvi18g04532\_t001 |  |  |  |  |  |  |  |  |  |  |  |  |
| 0 | Vitvi18g04533\_t001 |  |  |  |  |  |  |  |  |  |  |  |  |
| 0 | Vitvi18g01935\_t001 |  |  |  |  |  |  |  |  |  |  |  |  |
| 0 | Vitvi18g04534\_t001 |  |  |  |  |  |  |  |  |  |  |  |  |
| 0 | Vitvi18g04535\_t001 |  |  |  |  |  |  |  |  |  |  |  |  |
| 0 | Vitvi18g01937\_t001 |  |  |  |  |  |  |  |  |  |  |  |  |
| 0 | Vitvi18g04536\_t001 |  |  |  |  |  |  |  |  |  |  |  |  |
| 0 | Vitvi18g03084\_t001 |  |  |  |  |  |  |  |  |  |  |  |  |
| 0 | Vitvi18g01942\_t001 |  |  |  |  |  |  |  |  |  |  |  |  |
| 0 | Vitvi18g03085\_t001 |  |  |  |  |  |  |  |  |  |  |  |  |
| 0 | Vitvi18g03087\_t001 |  |  |  |  |  |  |  |  |  |  |  |  |
| 0 | Vitvi18g04537\_t001 |  |  |  |  |  |  |  |  |  |  |  |  |
| 0 | Vitvi18g01946\_t001 |  |  |  |  |  |  |  |  |  |  |  |  |
| 0 | Vitvi18g01947\_t001 |  |  |  |  |  |  |  |  |  |  |  |  |
| 0 | Vitvi18g01948\_t001 |  |  |  |  |  |  |  |  |  |  |  |  |
| 0 | Vitvi18g04538\_t001 |  |  |  |  |  |  |  |  |  |  |  |  |
| 0 | Vitvi18g01949\_t001 |  |  |  |  |  |  |  |  |  |  |  |  |
| 0 | Vitvi18g01950\_t001 |  |  |  |  |  |  |  |  |  |  |  |  |
| 0 | Vitvi18g04539\_t001 |  |  |  |  |  |  |  |  |  |  |  |  |
| 0 | Vitvi18g04540\_t001 |  |  |  |  |  |  |  |  |  |  |  |  |
| 0 | Vitvi18g04541\_t001 |  |  |  |  |  |  |  |  |  |  |  |  |
| 0 | Vitvi18g04542\_t001 |  |  |  |  |  |  |  |  |  |  |  |  |
| 0 | Vitvi18g03090\_t001 |  |  |  |  |  |  |  |  |  |  |  |  |
| 0 | Vitvi18g04543\_t001 |  |  |  |  |  |  |  |  |  |  |  |  |
| 0 | Vitvi18g04544\_t001 |  |  |  |  |  |  |  |  |  |  |  |  |
| 0 | Vitvi18g04545\_t001 |  |  |  |  |  |  |  |  |  |  |  |  |
| 0 | Vitvi18g03093\_t001 |  |  |  |  |  |  |  |  |  |  |  |  |
| 0 | Vitvi18g04546\_t001 |  |  |  |  |  |  |  |  |  |  |  |  |
| 0 | Vitvi18g01955\_t001 |  |  |  |  |  |  |  |  |  |  |  |  |
| 0 | Vitvi18g01958\_t001 |  |  |  |  |  |  |  |  |  |  |  |  |
| 0 | Vitvi18g04547\_t001 |  |  |  |  |  |  |  |  |  |  |  |  |
| 0 | Vitvi18g04548\_t001 |  |  |  |  |  |  |  |  |  |  |  |  |
| 0 | Vitvi18g04549\_t001 |  |  |  |  |  |  |  |  |  |  |  |  |
| 0 | Vitvi18g04550\_t001 |  |  |  |  |  |  |  |  |  |  |  |  |
| 0 | Vitvi18g01959\_t001 |  |  |  |  |  |  |  |  |  |  |  |  |
| 0 | Vitvi18g04551\_t001 |  |  |  |  |  |  |  |  |  |  |  |  |
| 0 | Vitvi18g03097\_t001 |  |  |  |  |  |  |  |  |  |  |  |  |
| 0 | Vitvi18g03098\_t001 |  |  |  |  |  |  |  |  |  |  |  |  |
| 0 | Vitvi18g01966\_t001 |  |  |  |  |  |  |  |  |  |  |  |  |
| 0 | Vitvi18g01967\_t001 |  |  |  |  |  |  |  |  |  |  |  |  |
| 0 | Vitvi18g01968\_t001 |  |  |  |  |  |  |  |  |  |  |  |  |
| 0 | Vitvi18g03099\_t001 |  |  |  |  |  |  |  |  |  |  |  |  |
| 0 | Vitvi18g01970\_t001 |  |  |  |  |  |  |  |  |  |  |  |  |
| 0 | Vitvi18g01971\_t002 |  |  |  |  |  |  |  |  |  |  |  |  |
| 0 | Vitvi18g03100\_t001 |  |  |  |  |  |  |  |  |  |  |  |  |
| 0 | Vitvi18g01972\_t001 |  |  |  |  |  |  |  |  |  |  |  |  |
| 0 | Vitvi18g01973\_t001 |  |  |  |  |  |  |  |  |  |  |  |  |
| 0 | Vitvi18g04552\_t001 |  |  |  |  |  |  |  |  |  |  |  |  |
| 0 | Vitvi18g01974\_t001 |  |  |  |  |  |  |  |  |  |  |  |  |
| 0 | Vitvi18g01975\_t001 |  |  |  |  |  |  |  |  |  |  |  |  |
| 0 | Vitvi18g03101\_t001 |  |  |  |  |  |  |  |  |  |  |  |  |
| 0 | Vitvi18g01977\_t001 |  |  |  |  |  |  |  |  |  |  |  |  |
| 0 | Vitvi18g01978\_t001 |  |  |  |  |  |  |  |  |  |  |  |  |
| 0 | Vitvi18g04553\_t001 |  |  |  |  |  |  |  |  |  |  |  |  |
| 0 | Vitvi18g04554\_t001 |  |  |  |  |  |  |  |  |  |  |  |  |
| 0 | Vitvi18g03104\_t001 |  |  |  |  |  |  |  |  |  |  |  |  |
| 0 | Vitvi18g01980\_t001 |  |  |  |  |  |  |  |  |  |  |  |  |
| 0 | Vitvi18g01981\_t001 |  |  |  |  |  |  |  |  |  |  |  |  |
| 0 | Vitvi18g04555\_t001 |  |  |  |  |  |  |  |  |  |  |  |  |
| 0 | Vitvi18g01982\_t001 |  |  |  |  |  |  |  |  |  |  |  |  |
| 0 | Vitvi18g01983\_t001 |  |  |  |  |  |  |  |  |  |  |  |  |
| 0 | Vitvi18g04556\_t001 |  |  |  |  |  |  |  |  |  |  |  |  |
| 0 | Vitvi18g04557\_t001 |  |  |  |  |  |  |  |  |  |  |  |  |
| 0 | Vitvi18g01986\_t001 |  |  |  |  |  |  |  |  |  |  |  |  |
| 0 | Vitvi18g01987\_t001 |  |  |  |  |  |  |  |  |  |  |  |  |
| 0 | Vitvi18g01988\_t001 |  |  |  |  |  |  |  |  |  |  |  |  |
| 0 | Vitvi18g01991\_t001 |  |  |  |  |  |  |  |  |  |  |  |  |
| 0 | Vitvi18g01992\_t001 |  |  |  |  |  |  |  |  |  |  |  |  |
| 0 | Vitvi18g01994\_t001 |  |  |  |  |  |  |  |  |  |  |  |  |
| 0 | Vitvi18g01995\_t001 |  |  |  |  |  |  |  |  |  |  |  |  |
| 0 | Vitvi18g04558\_t001 |  |  |  |  |  |  |  |  |  |  |  |  |
| 0 | Vitvi18g01997\_t001 |  |  |  |  |  |  |  |  |  |  |  |  |
| 0 | Vitvi18g04559\_t001 |  |  |  |  |  |  |  |  |  |  |  |  |
| 0 | Vitvi18g02003\_t001 |  |  |  |  |  |  |  |  |  |  |  |  |
| 0 | Vitvi18g02004\_t001 |  |  |  |  |  |  |  |  |  |  |  |  |
| 0 | Vitvi18g03111\_t001 |  |  |  |  |  |  |  |  |  |  |  |  |
| 0 | Vitvi18g03112\_t001 |  |  |  |  |  |  |  |  |  |  |  |  |
| 0 | Vitvi18g02006\_t001 |  |  |  |  |  |  |  |  |  |  |  |  |
| 0 | Vitvi18g02008\_t002 |  |  |  |  |  |  |  |  |  |  |  |  |
| 0 | Vitvi18g02011\_t001 |  |  |  |  |  |  |  |  |  |  |  |  |
| 0 | Vitvi18g02013\_t001 |  |  |  |  |  |  |  |  |  |  |  |  |
| 0 | Vitvi18g02015\_t001 |  |  |  |  |  |  |  |  |  |  |  |  |
| 0 | Vitvi18g04560\_t001 |  |  |  |  |  |  |  |  |  |  |  |  |
| 0 | Vitvi18g04561\_t001 |  |  |  |  |  |  |  |  |  |  |  |  |
| 0 | Vitvi18g02024\_t001 |  |  |  |  |  |  |  |  |  |  |  |  |
| 0 | Vitvi18g02033\_t001 |  |  |  |  |  |  |  |  |  |  |  |  |
| 0 | Vitvi18g04562\_t001 |  |  |  |  |  |  |  |  |  |  |  |  |
| 0 | Vitvi18g04563\_t001 |  |  |  |  |  |  |  |  |  |  |  |  |
| 0 | Vitvi18g04564\_t001 |  |  |  |  |  |  |  |  |  |  |  |  |
| 0 | Vitvi18g04565\_t001 |  |  |  |  |  |  |  |  |  |  |  |  |
| 0 | Vitvi18g04566\_t001 |  |  |  |  |  |  |  |  |  |  |  |  |
| 0 | Vitvi18g02048\_t001 |  |  |  |  |  |  |  |  |  |  |  |  |
| 0 | Vitvi18g02050\_t001 |  |  |  |  |  |  |  |  |  |  |  |  |
| 0 | Vitvi18g02051\_t001 |  |  |  |  |  |  |  |  |  |  |  |  |
| 0 | Vitvi18g02052\_t001 |  |  |  |  |  |  |  |  |  |  |  |  |
| 0 | Vitvi18g04568\_t001 |  |  |  |  |  |  |  |  |  |  |  |  |
| 0 | Vitvi18g04569\_t001 |  |  |  |  |  |  |  |  |  |  |  |  |
| 0 | Vitvi18g04570\_t001 |  |  |  |  |  |  |  |  |  |  |  |  |
| 0 | Vitvi18g04571\_t001 |  |  |  |  |  |  |  |  |  |  |  |  |
| 0 | Vitvi18g02059\_t001 |  |  |  |  |  |  |  |  |  |  |  |  |
| 0 | Vitvi18g03125\_t001 |  |  |  |  |  |  |  |  |  |  |  |  |
| 0 | Vitvi18g02060\_t001 |  |  |  |  |  |  |  |  |  |  |  |  |
| 0 | Vitvi18g03126\_t001 |  |  |  |  |  |  |  |  |  |  |  |  |
| 0 | Vitvi18g04572\_t001 |  |  |  |  |  |  |  |  |  |  |  |  |
| 0 | Vitvi18g02061\_t001 |  |  |  |  |  |  |  |  |  |  |  |  |
| 0 | Vitvi18g02063\_t001 |  |  |  |  |  |  |  |  |  |  |  |  |
| 0 | Vitvi18g02066\_t001 |  |  |  |  |  |  |  |  |  |  |  |  |
| 0 | Vitvi18g02067\_t001 |  |  |  |  |  |  |  |  |  |  |  |  |
| 0 | Vitvi18g02069\_t001 |  |  |  |  |  |  |  |  |  |  |  |  |
| 0 | Vitvi18g04573\_t001 |  |  |  |  |  |  |  |  |  |  |  |  |
| 0 | Vitvi18g03130\_t001 |  |  |  |  |  |  |  |  |  |  |  |  |
| 0 | Vitvi18g02072\_t001 |  |  |  |  |  |  |  |  |  |  |  |  |
| 0 | Vitvi18g04574\_t001 |  |  |  |  |  |  |  |  |  |  |  |  |
| 0 | Vitvi18g02075\_t001 |  |  |  |  |  |  |  |  |  |  |  |  |
| 0 | Vitvi18g04575\_t001 |  |  |  |  |  |  |  |  |  |  |  |  |
| 0 | Vitvi18g04576\_t001 |  |  |  |  |  |  |  |  |  |  |  |  |
| 0 | Vitvi18g02077\_t001 |  |  |  |  |  |  |  |  |  |  |  |  |
| 0 | Vitvi18g03133\_t001 |  |  |  |  |  |  |  |  |  |  |  |  |
| 0 | Vitvi18g04577\_t001 |  |  |  |  |  |  |  |  |  |  |  |  |
| 0 | Vitvi18g04578\_t001 |  |  |  |  |  |  |  |  |  |  |  |  |
| 0 | Vitvi18g02080\_t001 |  |  |  |  |  |  |  |  |  |  |  |  |
| 0 | Vitvi18g04579\_t001 |  |  |  |  |  |  |  |  |  |  |  |  |
| 0 | Vitvi18g02081\_t001 |  |  |  |  |  |  |  |  |  |  |  |  |
| 0 | Vitvi18g02082\_t001 |  |  |  |  |  |  |  |  |  |  |  |  |
| 0 | Vitvi18g04580\_t001 |  |  |  |  |  |  |  |  |  |  |  |  |
| 0 | Vitvi18g04581\_t001 |  |  |  |  |  |  |  |  |  |  |  |  |
| 0 | Vitvi18g04582\_t001 |  |  |  |  |  |  |  |  |  |  |  |  |
| 0 | Vitvi18g04583\_t001 |  |  |  |  |  |  |  |  |  |  |  |  |
| 0 | Vitvi18g02085\_t001 |  |  |  |  |  |  |  |  |  |  |  |  |
| 0 | Vitvi18g04584\_t001 |  |  |  |  |  |  |  |  |  |  |  |  |
| 0 | Vitvi18g04585\_t001 |  |  |  |  |  |  |  |  |  |  |  |  |
| 0 | Vitvi18g04586\_t001 |  |  |  |  |  |  |  |  |  |  |  |  |
| 0 | Vitvi18g04587\_t001 |  |  |  |  |  |  |  |  |  |  |  |  |
| 0 | Vitvi18g02091\_t001 |  |  |  |  |  |  |  |  |  |  |  |  |
| 0 | Vitvi18g02092\_t001 |  |  |  |  |  |  |  |  |  |  |  |  |
| 0 | Vitvi18g04588\_t001 |  |  |  |  |  |  |  |  |  |  |  |  |
| 0 | Vitvi18g04589\_t001 |  |  |  |  |  |  |  |  |  |  |  |  |
| 0 | Vitvi18g03138\_t001 |  |  |  |  |  |  |  |  |  |  |  |  |
| 0 | Vitvi18g02098\_t001 |  |  |  |  |  |  |  |  |  |  |  |  |
| 0 | Vitvi18g04590\_t001 |  |  |  |  |  |  |  |  |  |  |  |  |
| 0 | Vitvi18g03139\_t001 |  |  |  |  |  |  |  |  |  |  |  |  |
| 0 | Vitvi18g02099\_t001 |  |  |  |  |  |  |  |  |  |  |  |  |
| 1 | Vitvi18g03141\_t001 |  | Vitvi18g04748\_t001 |  |  |  |  |  |  |  |  |  |  |
| 1 | Vitvi18g03142\_t001 |  | \| \| |  |  |  |  |  |  |  |  |  |  |
| 1 | Vitvi18g04591\_t001 |  | \| \| |  |  |  |  |  |  |  |  |  |  |
| 1 | Vitvi18g04592\_t001 |  | \| \| |  |  |  |  |  |  |  |  |  |  |
| 1 | Vitvi18g04593\_t001 |  | Vitvi18g04749\_t001 |  |  |  |  |  |  |  |  |  |  |
| 1 | Vitvi18g04594\_t001 |  | \| \| |  |  |  |  |  |  |  |  |  |  |
| 1 | Vitvi18g02103\_t001 |  | \| \| |  |  |  |  |  |  |  |  |  |  |
| 1 | Vitvi18g04595\_t001 |  | \| \| |  |  |  |  |  |  |  |  |  |  |
| 1 | Vitvi18g04596\_t001 |  | \| \| |  |  |  |  |  |  |  |  |  |  |
| 1 | Vitvi18g03147\_t001 |  | \| \| |  |  |  |  |  |  |  |  |  |  |
| 1 | Vitvi18g04597\_t001 |  | Vitvi18g04758\_t001 |  |  |  |  |  |  |  |  |  |  |
| 1 | Vitvi18g04598\_t001 |  | \| \| |  |  |  |  |  |  |  |  |  |  |
| 1 | Vitvi18g04599\_t001 |  | \| \| |  |  |  |  |  |  |  |  |  |  |
| 1 | Vitvi18g04600\_t001 |  | \| \| |  |  |  |  |  |  |  |  |  |  |
| 1 | Vitvi18g04601\_t001 |  | \| \| |  |  |  |  |  |  |  |  |  |  |
| 1 | Vitvi18g03149\_t001 |  | \| \| |  |  |  |  |  |  |  |  |  |  |
| 1 | Vitvi18g04602\_t001 |  | \| \| |  |  |  |  |  |  |  |  |  |  |
| 1 | Vitvi18g04603\_t001 |  | \| \| |  |  |  |  |  |  |  |  |  |  |
| 1 | Vitvi18g03152\_t001 |  | \| \| |  |  |  |  |  |  |  |  |  |  |
| 1 | Vitvi18g04604\_t001 |  | \| \| |  |  |  |  |  |  |  |  |  |  |
| 1 | Vitvi18g04605\_t001 |  | \| \| |  |  |  |  |  |  |  |  |  |  |
| 1 | Vitvi18g04606\_t001 |  | Vitvi18g02375\_t001 |  |  |  |  |  |  |  |  |  |  |
| 1 | Vitvi18g02114\_t001 |  | \| \| |  |  |  |  |  |  |  |  |  |  |
| 1 | Vitvi18g02115\_t001 |  | \| \| |  |  |  |  |  |  |  |  |  |  |
| 1 | Vitvi18g02118\_t001 |  | \| \| |  |  |  |  |  |  |  |  |  |  |
| 1 | Vitvi18g04607\_t001 |  | \| \| |  |  |  |  |  |  |  |  |  |  |
| 1 | Vitvi18g04608\_t001 |  | \| \| |  |  |  |  |  |  |  |  |  |  |
| 1 | Vitvi18g04609\_t001 |  | \| \| |  |  |  |  |  |  |  |  |  |  |
| 1 | Vitvi18g02122\_t001 |  | \| \| |  |  |  |  |  |  |  |  |  |  |
| 1 | Vitvi18g02124\_t001 |  | \| \| |  |  |  |  |  |  |  |  |  |  |
| 1 | Vitvi18g02126\_t001 |  | \| \| |  |  |  |  |  |  |  |  |  |  |
| 1 | Vitvi18g04610\_t001 |  | \| \| |  |  |  |  |  |  |  |  |  |  |
| 1 | Vitvi18g02128\_t001 |  | \| \| |  |  |  |  |  |  |  |  |  |  |
| 1 | Vitvi18g03153\_t001 |  | \| \| |  |  |  |  |  |  |  |  |  |  |
| 1 | Vitvi18g02130\_t001 |  | \| \| |  |  |  |  |  |  |  |  |  |  |
| 1 | Vitvi18g02131\_t001 |  | \| \| |  |  |  |  |  |  |  |  |  |  |
| 1 | Vitvi18g02132\_t001 |  | \| \| |  |  |  |  |  |  |  |  |  |  |
| 1 | Vitvi18g02133\_t001 |  | \| \| |  |  |  |  |  |  |  |  |  |  |
| 1 | Vitvi18g04611\_t001 |  | \| \| |  |  |  |  |  |  |  |  |  |  |
| 1 | Vitvi18g04612\_t001 |  | \| \| |  |  |  |  |  |  |  |  |  |  |
| 1 | Vitvi18g04613\_t001 |  | \| \| |  |  |  |  |  |  |  |  |  |  |
| 1 | Vitvi18g04614\_t001 |  | \| \| |  |  |  |  |  |  |  |  |  |  |
| 1 | Vitvi18g04615\_t001 |  | Vitvi18g04773\_t001 |  |  |  |  |  |  |  |  |  |  |
| 1 | Vitvi18g04616\_t001 |  | \| \| |  |  |  |  |  |  |  |  |  |  |
| 1 | Vitvi18g04617\_t001 |  | \| \| |  |  |  |  |  |  |  |  |  |  |
| 1 | Vitvi18g04618\_t001 |  | Vitvi18g04775\_t001 |  |  |  |  |  |  |  |  |  |  |
| 1 | Vitvi18g04619\_t001 |  | Vitvi18g04776\_t001 |  |  |  |  |  |  |  |  |  |  |
| 1 | Vitvi18g02135\_t001 |  | \| \| |  |  |  |  |  |  |  |  |  |  |
| 1 | Vitvi18g04620\_t001 |  | \| \| |  |  |  |  |  |  |  |  |  |  |
| 1 | Vitvi18g04621\_t001 |  | \| \| |  |  |  |  |  |  |  |  |  |  |
| 1 | Vitvi18g03157\_t001 |  | \| \| |  |  |  |  |  |  |  |  |  |  |
| 1 | Vitvi18g02138\_t001 |  | \| \| |  |  |  |  |  |  |  |  |  |  |
| 1 | Vitvi18g02139\_t001 |  | \| \| |  |  |  |  |  |  |  |  |  |  |
| 1 | Vitvi18g02140\_t001 |  | \| \| |  |  |  |  |  |  |  |  |  |  |
| 1 | Vitvi18g04622\_t001 |  | \| \| |  |  |  |  |  |  |  |  |  |  |
| 1 | Vitvi18g04623\_t001 |  | \| \| |  |  |  |  |  |  |  |  |  |  |
| 1 | Vitvi18g04624\_t001 |  | \| \| |  |  |  |  |  |  |  |  |  |  |
| 1 | Vitvi18g03162\_t003 |  | \| \| |  |  |  |  |  |  |  |  |  |  |
| 1 | Vitvi18g02142\_t001 |  | \| \| |  |  |  |  |  |  |  |  |  |  |
| 1 | Vitvi18g03163\_t001 |  | \| \| |  |  |  |  |  |  |  |  |  |  |
| 1 | Vitvi18g03164\_t001 |  | \| \| |  |  |  |  |  |  |  |  |  |  |
| 1 | Vitvi18g02144\_t002 |  | \| \| |  |  |  |  |  |  |  |  |  |  |
| 1 | Vitvi18g02145\_t001 |  | \| \| |  |  |  |  |  |  |  |  |  |  |
| 1 | Vitvi18g02146\_t001 |  | Vitvi18g02393\_t001 |  |  |  |  |  |  |  |  |  |  |
| 0 | Vitvi18g02147\_t001 |  |  |  |  |  |  |  |  |  |  |  |  |
| 0 | Vitvi18g02148\_t001 |  |  |  |  |  |  |  |  |  |  |  |  |
| 0 | Vitvi18g02149\_t001 |  |  |  |  |  |  |  |  |  |  |  |  |
| 0 | Vitvi18g04625\_t001 |  |  |  |  |  |  |  |  |  |  |  |  |
| 0 | Vitvi18g04626\_t001 |  |  |  |  |  |  |  |  |  |  |  |  |
| 0 | Vitvi18g03166\_t001 |  |  |  |  |  |  |  |  |  |  |  |  |
| 0 | Vitvi18g03167\_t001 |  |  |  |  |  |  |  |  |  |  |  |  |
| 0 | Vitvi18g04627\_t001 |  |  |  |  |  |  |  |  |  |  |  |  |
| 0 | Vitvi18g04628\_t001 |  |  |  |  |  |  |  |  |  |  |  |  |
| 0 | Vitvi18g03169\_t001 |  |  |  |  |  |  |  |  |  |  |  |  |
| 0 | Vitvi18g02152\_t001 |  |  |  |  |  |  |  |  |  |  |  |  |
| 0 | Vitvi18g02153\_t001 |  |  |  |  |  |  |  |  |  |  |  |  |
| 0 | Vitvi18g04629\_t001 |  |  |  |  |  |  |  |  |  |  |  |  |
| 0 | Vitvi18g04630\_t001 |  |  |  |  |  |  |  |  |  |  |  |  |
| 0 | Vitvi18g04631\_t001 |  |  |  |  |  |  |  |  |  |  |  |  |
| 0 | Vitvi18g04632\_t001 |  |  |  |  |  |  |  |  |  |  |  |  |
| 0 | Vitvi18g02156\_t001 |  |  |  |  |  |  |  |  |  |  |  |  |
| 0 | Vitvi18g02157\_t001 |  |  |  |  |  |  |  |  |  |  |  |  |
| 0 | Vitvi18g04633\_t001 |  |  |  |  |  |  |  |  |  |  |  |  |
| 0 | Vitvi18g04634\_t001 |  |  |  |  |  |  |  |  |  |  |  |  |
| 0 | Vitvi18g04635\_t001 |  |  |  |  |  |  |  |  |  |  |  |  |
| 0 | Vitvi18g04636\_t001 |  |  |  |  |  |  |  |  |  |  |  |  |
| 0 | Vitvi18g02159\_t001 |  |  |  |  |  |  |  |  |  |  |  |  |
| 0 | Vitvi18g02164\_t001 |  |  |  |  |  |  |  |  |  |  |  |  |
| 0 | Vitvi18g02165\_t001 |  |  |  |  |  |  |  |  |  |  |  |  |
| 0 | Vitvi18g04637\_t001 |  |  |  |  |  |  |  |  |  |  |  |  |
| 0 | Vitvi18g02167\_t001 |  |  |  |  |  |  |  |  |  |  |  |  |
| 0 | Vitvi18g04638\_t001 |  |  |  |  |  |  |  |  |  |  |  |  |
| 0 | Vitvi18g02170\_t001 |  |  |  |  |  |  |  |  |  |  |  |  |
| 0 | Vitvi18g02171\_t001 |  |  |  |  |  |  |  |  |  |  |  |  |
| 0 | Vitvi18g02173\_t001 |  |  |  |  |  |  |  |  |  |  |  |  |
| 0 | Vitvi18g02180\_t001 |  |  |  |  |  |  |  |  |  |  |  |  |
| 0 | Vitvi18g03383\_t001 |  |  |  |  |  |  |  |  |  |  |  |  |
| 0 | Vitvi18g04639\_t001 |  |  |  |  |  |  |  |  |  |  |  |  |
| 0 | Vitvi18g03385\_t001 |  |  |  |  |  |  |  |  |  |  |  |  |
| 0 | Vitvi18g04640\_t001 |  |  |  |  |  |  |  |  |  |  |  |  |
| 0 | Vitvi18g04641\_t001 |  |  |  |  |  |  |  |  |  |  |  |  |
| 0 | Vitvi18g03375\_t001 |  |  |  |  |  |  |  |  |  |  |  |  |
| 0 | Vitvi18g03374\_t001 |  |  |  |  |  |  |  |  |  |  |  |  |
| 0 | Vitvi18g04642\_t001 |  |  |  |  |  |  |  |  |  |  |  |  |
| 0 | Vitvi18g04643\_t001 |  |  |  |  |  |  |  |  |  |  |  |  |
| 0 | Vitvi18g04644\_t001 |  |  |  |  |  |  |  |  |  |  |  |  |
| 0 | Vitvi18g02183\_t001 |  |  |  |  |  |  |  |  |  |  |  |  |
| 0 | Vitvi18g02184\_t001 |  |  |  |  |  |  |  |  |  |  |  |  |
| 0 | Vitvi18g02185\_t001 |  |  |  |  |  |  |  |  |  |  |  |  |
| 0 | Vitvi18g02186\_t002 |  |  |  |  |  |  |  |  |  |  |  |  |
| 0 | Vitvi18g02187\_t001 |  |  |  |  |  |  |  |  |  |  |  |  |
| 0 | Vitvi18g02188\_t001 |  |  |  |  |  |  |  |  |  |  |  |  |
| 0 | Vitvi18g02190\_t001 |  |  |  |  |  |  |  |  |  |  |  |  |
| 0 | Vitvi18g04645\_t001 |  |  |  |  |  |  |  |  |  |  |  |  |
| 0 | Vitvi18g02191\_t001 |  |  |  |  |  |  |  |  |  |  |  |  |
| 0 | Vitvi18g02192\_t001 |  |  |  |  |  |  |  |  |  |  |  |  |
| 0 | Vitvi18g04646\_t001 |  |  |  |  |  |  |  |  |  |  |  |  |
| 0 | Vitvi18g04647\_t001 |  |  |  |  |  |  |  |  |  |  |  |  |
| 0 | Vitvi18g02195\_t001 |  |  |  |  |  |  |  |  |  |  |  |  |
| 0 | Vitvi18g04648\_t001 |  |  |  |  |  |  |  |  |  |  |  |  |
| 0 | Vitvi18g04649\_t001 |  |  |  |  |  |  |  |  |  |  |  |  |
| 0 | Vitvi18g04650\_t001 |  |  |  |  |  |  |  |  |  |  |  |  |
| 0 | Vitvi18g04651\_t001 |  |  |  |  |  |  |  |  |  |  |  |  |
| 0 | Vitvi18g03185\_t001 |  |  |  |  |  |  |  |  |  |  |  |  |
| 0 | Vitvi18g04652\_t001 |  |  |  |  |  |  |  |  |  |  |  |  |
| 0 | Vitvi18g04653\_t001 |  |  |  |  |  |  |  |  |  |  |  |  |
| 0 | Vitvi18g03191\_t001 |  |  |  |  |  |  |  |  |  |  |  |  |
| 0 | Vitvi18g04654\_t001 |  |  |  |  |  |  |  |  |  |  |  |  |
| 0 | Vitvi18g04655\_t001 |  |  |  |  |  |  |  |  |  |  |  |  |
| 0 | Vitvi18g02210\_t001 |  |  |  |  |  |  |  |  |  |  |  |  |
| 0 | Vitvi18g04656\_t001 |  |  |  |  |  |  |  |  |  |  |  |  |
| 0 | Vitvi18g04657\_t001 |  |  |  |  |  |  |  |  |  |  |  |  |
| 0 | Vitvi18g04658\_t001 |  |  |  |  |  |  |  |  |  |  |  |  |
| 0 | Vitvi18g04659\_t001 |  |  |  |  |  |  |  |  |  |  |  |  |
| 0 | Vitvi18g04660\_t001 |  |  |  |  |  |  |  |  |  |  |  |  |
| 0 | Vitvi18g04661\_t001 |  |  |  |  |  |  |  |  |  |  |  |  |
| 0 | Vitvi18g04662\_t001 |  |  |  |  |  |  |  |  |  |  |  |  |
| 0 | Vitvi18g04663\_t001 |  |  |  |  |  |  |  |  |  |  |  |  |
| 0 | Vitvi18g04664\_t001 |  |  |  |  |  |  |  |  |  |  |  |  |
| 0 | Vitvi18g02221\_t002 |  |  |  |  |  |  |  |  |  |  |  |  |
| 0 | Vitvi18g02222\_t002 |  |  |  |  |  |  |  |  |  |  |  |  |
| 0 | Vitvi18g02223\_t001 |  |  |  |  |  |  |  |  |  |  |  |  |
| 0 | Vitvi18g04665\_t001 |  |  |  |  |  |  |  |  |  |  |  |  |
| 0 | Vitvi18g04666\_t001 |  |  |  |  |  |  |  |  |  |  |  |  |
| 0 | Vitvi18g04667\_t001 |  |  |  |  |  |  |  |  |  |  |  |  |
| 0 | Vitvi18g04668\_t001 |  |  |  |  |  |  |  |  |  |  |  |  |
| 0 | Vitvi18g04669\_t001 |  |  |  |  |  |  |  |  |  |  |  |  |
| 0 | Vitvi18g02228\_t002 |  |  |  |  |  |  |  |  |  |  |  |  |
| 0 | Vitvi18g02230\_t001 |  |  |  |  |  |  |  |  |  |  |  |  |
| 0 | Vitvi18g02231\_t001 |  |  |  |  |  |  |  |  |  |  |  |  |
| 0 | Vitvi18g02232\_t001 |  |  |  |  |  |  |  |  |  |  |  |  |
| 0 | Vitvi18g02233\_t001 |  |  |  |  |  |  |  |  |  |  |  |  |
| 0 | Vitvi18g04670\_t001 |  |  |  |  |  |  |  |  |  |  |  |  |
| 0 | Vitvi18g04671\_t001 |  |  |  |  |  |  |  |  |  |  |  |  |
| 0 | Vitvi18g02236\_t001 |  |  |  |  |  |  |  |  |  |  |  |  |
| 0 | Vitvi18g02237\_t001 |  |  |  |  |  |  |  |  |  |  |  |  |
| 0 | Vitvi18g02238\_t001 |  |  |  |  |  |  |  |  |  |  |  |  |
| 0 | Vitvi18g02240\_t001 |  |  |  |  |  |  |  |  |  |  |  |  |
| 0 | Vitvi18g02241\_t001 |  |  |  |  |  |  |  |  |  |  |  |  |
| 0 | Vitvi18g04672\_t001 |  |  |  |  |  |  |  |  |  |  |  |  |
| 0 | Vitvi18g02242\_t001 |  |  |  |  |  |  |  |  |  |  |  |  |
| 0 | Vitvi18g04673\_t001 |  |  |  |  |  |  |  |  |  |  |  |  |
| 0 | Vitvi18g02244\_t001 |  |  |  |  |  |  |  |  |  |  |  |  |
| 0 | Vitvi18g02246\_t001 |  |  |  |  |  |  |  |  |  |  |  |  |
| 0 | Vitvi18g04674\_t001 |  |  |  |  |  |  |  |  |  |  |  |  |
| 0 | Vitvi18g02248\_t001 |  |  |  |  |  |  |  |  |  |  |  |  |
| 0 | Vitvi18g02249\_t001 |  |  |  |  |  |  |  |  |  |  |  |  |
| 0 | Vitvi18g04675\_t001 |  |  |  |  |  |  |  |  |  |  |  |  |
| 0 | Vitvi18g04676\_t001 |  |  |  |  |  |  |  |  |  |  |  |  |
| 0 | Vitvi18g04677\_t001 |  |  |  |  |  |  |  |  |  |  |  |  |
| 0 | Vitvi18g04678\_t001 |  |  |  |  |  |  |  |  |  |  |  |  |
| 0 | Vitvi18g02251\_t002 |  |  |  |  |  |  |  |  |  |  |  |  |
| 0 | Vitvi18g02253\_t002 |  |  |  |  |  |  |  |  |  |  |  |  |
| 0 | Vitvi18g04679\_t001 |  |  |  |  |  |  |  |  |  |  |  |  |
| 0 | Vitvi18g04680\_t001 |  |  |  |  |  |  |  |  |  |  |  |  |
| 0 | Vitvi18g04681\_t001 |  |  |  |  |  |  |  |  |  |  |  |  |
| 0 | Vitvi18g04682\_t001 |  |  |  |  |  |  |  |  |  |  |  |  |
| 0 | Vitvi18g02467\_t001 |  |  |  |  |  |  |  |  |  |  |  |  |
| 0 | Vitvi18g04683\_t001 |  |  |  |  |  |  |  |  |  |  |  |  |
| 0 | Vitvi18g04684\_t001 |  |  |  |  |  |  |  |  |  |  |  |  |
| 0 | Vitvi18g02451\_t001 |  |  |  |  |  |  |  |  |  |  |  |  |
| 0 | Vitvi18g04685\_t001 |  |  |  |  |  |  |  |  |  |  |  |  |
| 0 | Vitvi18g03207\_t001 |  |  |  |  |  |  |  |  |  |  |  |  |
| 0 | Vitvi18g02261\_t001 |  |  |  |  |  |  |  |  |  |  |  |  |
| 0 | Vitvi18g04686\_t001 |  |  |  |  |  |  |  |  |  |  |  |  |
| 0 | Vitvi18g04687\_t001 |  |  |  |  |  |  |  |  |  |  |  |  |
| 0 | Vitvi18g02263\_t001 |  |  |  |  |  |  |  |  |  |  |  |  |
| 0 | Vitvi18g04688\_t001 |  |  |  |  |  |  |  |  |  |  |  |  |
| 0 | Vitvi18g04689\_t001 |  |  |  |  |  |  |  |  |  |  |  |  |
| 0 | Vitvi18g04690\_t001 |  |  |  |  |  |  |  |  |  |  |  |  |
| 0 | Vitvi18g04691\_t001 |  |  |  |  |  |  |  |  |  |  |  |  |
| 0 | Vitvi18g04692\_t001 |  |  |  |  |  |  |  |  |  |  |  |  |
| 0 | Vitvi18g04693\_t001 |  |  |  |  |  |  |  |  |  |  |  |  |
| 0 | Vitvi18g04694\_t001 |  |  |  |  |  |  |  |  |  |  |  |  |
| 0 | Vitvi18g02276\_t001 |  |  |  |  |  |  |  |  |  |  |  |  |
| 0 | Vitvi18g04695\_t001 |  |  |  |  |  |  |  |  |  |  |  |  |
| 0 | Vitvi18g04696\_t001 |  |  |  |  |  |  |  |  |  |  |  |  |
| 0 | Vitvi18g04697\_t001 |  |  |  |  |  |  |  |  |  |  |  |  |
| 0 | Vitvi18g04698\_t001 |  |  |  |  |  |  |  |  |  |  |  |  |
| 0 | Vitvi18g04699\_t001 |  |  |  |  |  |  |  |  |  |  |  |  |
| 0 | Vitvi18g02460\_t001 |  |  |  |  |  |  |  |  |  |  |  |  |
| 0 | Vitvi18g04700\_t001 |  |  |  |  |  |  |  |  |  |  |  |  |
| 0 | Vitvi18g04701\_t001 |  |  |  |  |  |  |  |  |  |  |  |  |
| 0 | Vitvi18g04702\_t001 |  |  |  |  |  |  |  |  |  |  |  |  |
| 0 | Vitvi18g04703\_t001 |  |  |  |  |  |  |  |  |  |  |  |  |
| 0 | Vitvi18g04704\_t001 |  |  |  |  |  |  |  |  |  |  |  |  |
| 0 | Vitvi18g04705\_t001 |  |  |  |  |  |  |  |  |  |  |  |  |
| 0 | Vitvi18g04706\_t001 |  |  |  |  |  |  |  |  |  |  |  |  |
| 0 | Vitvi18g04707\_t001 |  |  |  |  |  |  |  |  |  |  |  |  |
| 0 | Vitvi18g04708\_t001 |  |  |  |  |  |  |  |  |  |  |  |  |
| 0 | Vitvi18g04709\_t001 |  |  |  |  |  |  |  |  |  |  |  |  |
| 0 | Vitvi18g02446\_t001 |  |  |  |  |  |  |  |  |  |  |  |  |
| 0 | Vitvi18g04710\_t001 |  |  |  |  |  |  |  |  |  |  |  |  |
| 0 | Vitvi18g04711\_t001 |  |  |  |  |  |  |  |  |  |  |  |  |
| 0 | Vitvi18g04712\_t001 |  |  |  |  |  |  |  |  |  |  |  |  |
| 0 | Vitvi18g04713\_t001 |  |  |  |  |  |  |  |  |  |  |  |  |
| 0 | Vitvi18g04714\_t001 |  |  |  |  |  |  |  |  |  |  |  |  |
| 0 | Vitvi18g04715\_t001 |  |  |  |  |  |  |  |  |  |  |  |  |
| 0 | Vitvi18g04716\_t001 |  |  |  |  |  |  |  |  |  |  |  |  |
| 0 | Vitvi18g04717\_t001 |  |  |  |  |  |  |  |  |  |  |  |  |
| 0 | Vitvi18g04718\_t001 |  |  |  |  |  |  |  |  |  |  |  |  |
| 0 | Vitvi18g04719\_t001 |  |  |  |  |  |  |  |  |  |  |  |  |
| 0 | Vitvi18g02298\_t001 |  |  |  |  |  |  |  |  |  |  |  |  |
| 0 | Vitvi18g04720\_t001 |  |  |  |  |  |  |  |  |  |  |  |  |
| 0 | Vitvi18g04721\_t001 |  |  |  |  |  |  |  |  |  |  |  |  |
| 0 | Vitvi18g02459\_t001 |  |  |  |  |  |  |  |  |  |  |  |  |
| 0 | Vitvi18g02305\_t001 |  |  |  |  |  |  |  |  |  |  |  |  |
| 0 | Vitvi18g04722\_t001 |  |  |  |  |  |  |  |  |  |  |  |  |
| 0 | Vitvi18g04723\_t001 |  |  |  |  |  |  |  |  |  |  |  |  |
| 0 | Vitvi18g03214\_t001 |  |  |  |  |  |  |  |  |  |  |  |  |
| 0 | Vitvi18g04724\_t001 |  |  |  |  |  |  |  |  |  |  |  |  |
| 0 | Vitvi18g03215\_t001 |  |  |  |  |  |  |  |  |  |  |  |  |
| 0 | Vitvi18g04725\_t001 |  |  |  |  |  |  |  |  |  |  |  |  |
| 0 | Vitvi18g03217\_t001 |  |  |  |  |  |  |  |  |  |  |  |  |
| 0 | Vitvi18g02311\_t001 |  |  |  |  |  |  |  |  |  |  |  |  |
| 0 | Vitvi18g02312\_t001 |  |  |  |  |  |  |  |  |  |  |  |  |
| 0 | Vitvi18g04726\_t001 |  |  |  |  |  |  |  |  |  |  |  |  |
| 0 | Vitvi18g04727\_t001 |  |  |  |  |  |  |  |  |  |  |  |  |
| 0 | Vitvi18g04728\_t001 |  |  |  |  |  |  |  |  |  |  |  |  |
| 0 | Vitvi18g02316\_t003 |  |  |  |  |  |  |  |  |  |  |  |  |
| 0 | Vitvi18g02319\_t001 |  |  |  |  |  |  |  |  |  |  |  |  |
| 0 | Vitvi18g02320\_t001 |  |  |  |  |  |  |  |  |  |  |  |  |
| 0 | Vitvi18g02321\_t001 |  |  |  |  |  |  |  |  |  |  |  |  |
| 0 | Vitvi18g02322\_t001 |  |  |  |  |  |  |  |  |  |  |  |  |
| 0 | Vitvi18g02323\_t001 |  |  |  |  |  |  |  |  |  |  |  |  |
| 0 | Vitvi18g02324\_t001 |  |  |  |  |  |  |  |  |  |  |  |  |
| 0 | Vitvi18g04729\_t001 |  |  |  |  |  |  |  |  |  |  |  |  |
| 0 | Vitvi18g04730\_t001 |  |  |  |  |  |  |  |  |  |  |  |  |
| 0 | Vitvi18g02327\_t001 |  |  |  |  |  |  |  |  |  |  |  |  |
| 0 | Vitvi18g04731\_t001 |  |  |  |  |  |  |  |  |  |  |  |  |
| 0 | Vitvi18g04732\_t001 |  |  |  |  |  |  |  |  |  |  |  |  |
| 0 | Vitvi18g02328\_t002 |  |  |  |  |  |  |  |  |  |  |  |  |
| 0 | Vitvi18g02329\_t001 |  |  |  |  |  |  |  |  |  |  |  |  |
| 0 | Vitvi18g04733\_t001 |  |  |  |  |  |  |  |  |  |  |  |  |
| 0 | Vitvi18g04734\_t001 |  |  |  |  |  |  |  |  |  |  |  |  |
| 0 | Vitvi18g02333\_t001 |  |  |  |  |  |  |  |  |  |  |  |  |
| 0 | Vitvi18g04735\_t001 |  |  |  |  |  |  |  |  |  |  |  |  |
| 0 | Vitvi18g04736\_t001 |  |  |  |  |  |  |  |  |  |  |  |  |
| 0 | Vitvi18g04737\_t001 |  |  |  |  |  |  |  |  |  |  |  |  |
| 0 | Vitvi18g04738\_t001 |  |  |  |  |  |  |  |  |  |  |  |  |
| 0 | Vitvi18g02337\_t001 |  |  |  |  |  |  |  |  |  |  |  |  |
| 0 | Vitvi18g04739\_t001 |  |  |  |  |  |  |  |  |  |  |  |  |
| 0 | Vitvi18g04740\_t001 |  |  |  |  |  |  |  |  |  |  |  |  |
| 0 | Vitvi18g02341\_t001 |  |  |  |  |  |  |  |  |  |  |  |  |
| 0 | Vitvi18g03225\_t001 |  |  |  |  |  |  |  |  |  |  |  |  |
| 0 | Vitvi18g03226\_t001 |  |  |  |  |  |  |  |  |  |  |  |  |
| 0 | Vitvi18g04741\_t001 |  |  |  |  |  |  |  |  |  |  |  |  |
| 0 | Vitvi18g03228\_t001 |  |  |  |  |  |  |  |  |  |  |  |  |
| 0 | Vitvi18g02343\_t001 |  |  |  |  |  |  |  |  |  |  |  |  |
| 0 | Vitvi18g02345\_t001 |  |  |  |  |  |  |  |  |  |  |  |  |
| 0 | Vitvi18g02346\_t001 |  |  |  |  |  |  |  |  |  |  |  |  |
| 0 | Vitvi18g04742\_t001 |  |  |  |  |  |  |  |  |  |  |  |  |
| 0 | Vitvi18g02350\_t001 |  |  |  |  |  |  |  |  |  |  |  |  |
| 0 | Vitvi18g04743\_t001 |  |  |  |  |  |  |  |  |  |  |  |  |
| 0 | Vitvi18g02352\_t002 |  |  |  |  |  |  |  |  |  |  |  |  |
| 0 | Vitvi18g04744\_t001 |  |  |  |  |  |  |  |  |  |  |  |  |
| 0 | Vitvi18g03231\_t001 |  |  |  |  |  |  |  |  |  |  |  |  |
| 0 | Vitvi18g04745\_t001 |  |  |  |  |  |  |  |  |  |  |  |  |
| 0 | Vitvi18g04746\_t001 |  |  |  |  |  |  |  |  |  |  |  |  |
| 0 | Vitvi18g04747\_t001 |  |  |  |  |  |  |  |  |  |  |  |  |
| 1 | Vitvi18g04748\_t001 |  | Vitvi18g03141\_t001 |  |  |  |  |  |  |  |  |  |  |
| 1 | Vitvi18g04749\_t001 |  | Vitvi18g04593\_t001 |  |  |  |  |  |  |  |  |  |  |
| 1 | Vitvi18g04750\_t001 |  | \| \| |  |  |  |  |  |  |  |  |  |  |
| 1 | Vitvi18g02358\_t004 |  | \| \| |  |  |  |  |  |  |  |  |  |  |
| 1 | Vitvi18g02359\_t001 |  | \| \| |  |  |  |  |  |  |  |  |  |  |
| 1 | Vitvi18g02360\_t001 |  | \| \| |  |  |  |  |  |  |  |  |  |  |
| 1 | Vitvi18g02361\_t001 |  | \| \| |  |  |  |  |  |  |  |  |  |  |
| 1 | Vitvi18g03235\_t001 |  | \| \| |  |  |  |  |  |  |  |  |  |  |
| 1 | Vitvi18g02362\_t001 |  | \| \| |  |  |  |  |  |  |  |  |  |  |
| 1 | Vitvi18g02363\_t001 |  | \| \| |  |  |  |  |  |  |  |  |  |  |
| 1 | Vitvi18g04751\_t001 |  | \| \| |  |  |  |  |  |  |  |  |  |  |
| 1 | Vitvi18g02364\_t001 |  | \| \| |  |  |  |  |  |  |  |  |  |  |
| 1 | Vitvi18g02365\_t001 |  | \| \| |  |  |  |  |  |  |  |  |  |  |
| 1 | Vitvi18g03236\_t001 |  | \| \| |  |  |  |  |  |  |  |  |  |  |
| 1 | Vitvi18g04752\_t001 |  | \| \| |  |  |  |  |  |  |  |  |  |  |
| 1 | Vitvi18g04753\_t001 |  | \| \| |  |  |  |  |  |  |  |  |  |  |
| 1 | Vitvi18g03239\_t001 |  | \| \| |  |  |  |  |  |  |  |  |  |  |
| 1 | Vitvi18g04754\_t001 |  | \| \| |  |  |  |  |  |  |  |  |  |  |
| 1 | Vitvi18g04755\_t001 |  | \| \| |  |  |  |  |  |  |  |  |  |  |
| 1 | Vitvi18g04756\_t001 |  | \| \| |  |  |  |  |  |  |  |  |  |  |
| 1 | Vitvi18g04757\_t001 |  | \| \| |  |  |  |  |  |  |  |  |  |  |
| 1 | Vitvi18g04758\_t001 |  | Vitvi18g04597\_t001 |  |  |  |  |  |  |  |  |  |  |
| 1 | Vitvi18g04759\_t001 |  | \| \| |  |  |  |  |  |  |  |  |  |  |
| 1 | Vitvi18g04760\_t001 |  | \| \| |  |  |  |  |  |  |  |  |  |  |
| 1 | Vitvi18g04761\_t001 |  | \| \| |  |  |  |  |  |  |  |  |  |  |
| 1 | Vitvi18g04762\_t001 |  | \| \| |  |  |  |  |  |  |  |  |  |  |
| 1 | Vitvi18g02375\_t001 |  | Vitvi18g04606\_t001 |  |  |  |  |  |  |  |  |  |  |
| 1 | Vitvi18g02378\_t001 |  | \| \| |  |  |  |  |  |  |  |  |  |  |
| 1 | Vitvi18g04763\_t001 |  | \| \| |  |  |  |  |  |  |  |  |  |  |
| 1 | Vitvi18g04764\_t001 |  | \| \| |  |  |  |  |  |  |  |  |  |  |
| 1 | Vitvi18g04765\_t001 |  | \| \| |  |  |  |  |  |  |  |  |  |  |
| 1 | Vitvi18g04766\_t001 |  | \| \| |  |  |  |  |  |  |  |  |  |  |
| 1 | Vitvi18g02379\_t001 |  | \| \| |  |  |  |  |  |  |  |  |  |  |
| 1 | Vitvi18g02380\_t001 |  | \| \| |  |  |  |  |  |  |  |  |  |  |
| 1 | Vitvi18g02381\_t001 |  | \| \| |  |  |  |  |  |  |  |  |  |  |
| 1 | Vitvi18g03244\_t001 |  | \| \| |  |  |  |  |  |  |  |  |  |  |
| 1 | Vitvi18g03246\_t001 |  | \| \| |  |  |  |  |  |  |  |  |  |  |
| 1 | Vitvi18g04767\_t001 |  | \| \| |  |  |  |  |  |  |  |  |  |  |
| 1 | Vitvi18g04768\_t001 |  | \| \| |  |  |  |  |  |  |  |  |  |  |
| 1 | Vitvi18g04769\_t001 |  | \| \| |  |  |  |  |  |  |  |  |  |  |
| 1 | Vitvi18g03248\_t001 |  | \| \| |  |  |  |  |  |  |  |  |  |  |
| 1 | Vitvi18g03249\_t001 |  | \| \| |  |  |  |  |  |  |  |  |  |  |
| 1 | Vitvi18g03250\_t001 |  | \| \| |  |  |  |  |  |  |  |  |  |  |
| 1 | Vitvi18g04770\_t001 |  | \| \| |  |  |  |  |  |  |  |  |  |  |
| 1 | Vitvi18g03251\_t001 |  | \| \| |  |  |  |  |  |  |  |  |  |  |
| 1 | Vitvi18g04771\_t001 |  | \| \| |  |  |  |  |  |  |  |  |  |  |
| 1 | Vitvi18g03252\_t001 |  | \| \| |  |  |  |  |  |  |  |  |  |  |
| 1 | Vitvi18g04772\_t001 |  | \| \| |  |  |  |  |  |  |  |  |  |  |
| 1 | Vitvi18g04773\_t001 |  | Vitvi18g04615\_t001 |  |  |  |  |  |  |  |  |  |  |
| 1 | Vitvi18g04774\_t001 |  | \| \| |  |  |  |  |  |  |  |  |  |  |
| 1 | Vitvi18g04775\_t001 |  | Vitvi18g04618\_t001 |  |  |  |  |  |  |  |  |  |  |
| 1 | Vitvi18g04776\_t001 |  | Vitvi18g04619\_t001 |  |  |  |  |  |  |  |  |  |  |
| 1 | Vitvi18g04777\_t001 |  | \| \| |  |  |  |  |  |  |  |  |  |  |
| 1 | Vitvi18g04778\_t001 |  | \| \| |  |  |  |  |  |  |  |  |  |  |
| 1 | Vitvi18g04779\_t001 |  | \| \| |  |  |  |  |  |  |  |  |  |  |
| 1 | Vitvi18g04780\_t001 |  | \| \| |  |  |  |  |  |  |  |  |  |  |
| 1 | Vitvi18g04781\_t001 |  | \| \| |  |  |  |  |  |  |  |  |  |  |
| 1 | Vitvi18g03257\_t001 |  | \| \| |  |  |  |  |  |  |  |  |  |  |
| 1 | Vitvi18g03258\_t002 |  | \| \| |  |  |  |  |  |  |  |  |  |  |
| 1 | Vitvi18g02388\_t001 |  | \| \| |  |  |  |  |  |  |  |  |  |  |
| 1 | Vitvi18g02389\_t001 |  | \| \| |  |  |  |  |  |  |  |  |  |  |
| 1 | Vitvi18g02390\_t002 |  | \| \| |  |  |  |  |  |  |  |  |  |  |
| 1 | Vitvi18g02391\_t001 |  | \| \| |  |  |  |  |  |  |  |  |  |  |
| 1 | Vitvi18g02392\_t001 |  | \| \| |  |  |  |  |  |  |  |  |  |  |
| 1 | Vitvi18g02393\_t001 |  | Vitvi18g02146\_t001 |  |  |  |  |  |  |  |  |  |  |
| 0 | Vitvi18g04782\_t001 |  |  |  |  |  |  |  |  |  |  |  |  |
| 0 | Vitvi18g04783\_t001 |  |  |  |  |  |  |  |  |  |  |  |  |
| 0 | Vitvi18g04784\_t001 |  |  |  |  |  |  |  |  |  |  |  |  |
| 0 | Vitvi18g04785\_t001 |  |  |  |  |  |  |  |  |  |  |  |  |
| 0 | Vitvi18g04786\_t001 |  |  |  |  |  |  |  |  |  |  |  |  |
| 0 | Vitvi18g04787\_t001 |  |  |  |  |  |  |  |  |  |  |  |  |
| 0 | Vitvi18g04788\_t003 |  |  |  |  |  |  |  |  |  |  |  |  |
| 0 | Vitvi18g04789\_t001 |  |  |  |  |  |  |  |  |  |  |  |  |
| 0 | Vitvi18g04790\_t001 |  |  |  |  |  |  |  |  |  |  |  |  |
| 0 | Vitvi18g03265\_t001 |  |  |  |  |  |  |  |  |  |  |  |  |
| 0 | Vitvi18g03266\_t001 |  |  |  |  |  |  |  |  |  |  |  |  |
| 0 | Vitvi18g02396\_t001 |  |  |  |  |  |  |  |  |  |  |  |  |
| 0 | Vitvi18g03267\_t001 |  |  |  |  |  |  |  |  |  |  |  |  |
| 0 | Vitvi18g04791\_t001 |  |  |  |  |  |  |  |  |  |  |  |  |
| 0 | Vitvi18g03268\_t001 |  |  |  |  |  |  |  |  |  |  |  |  |
| 0 | Vitvi18g03269\_t001 |  |  |  |  |  |  |  |  |  |  |  |  |
| 0 | Vitvi18g02398\_t001 |  |  |  |  |  |  |  |  |  |  |  |  |
| 0 | Vitvi18g02399\_t001 |  |  |  |  |  |  |  |  |  |  |  |  |
| 0 | Vitvi18g02400\_t001 |  |  |  |  |  |  |  |  |  |  |  |  |
| 0 | Vitvi18g03270\_t001 |  |  |  |  |  |  |  |  |  |  |  |  |
| 0 | Vitvi18g02401\_t001 |  |  |  |  |  |  |  |  |  |  |  |  |
| 0 | Vitvi18g04792\_t001 |  |  |  |  |  |  |  |  |  |  |  |  |
| 0 | Vitvi18g04793\_t001 |  |  |  |  |  |  |  |  |  |  |  |  |
| 0 | Vitvi18g02403\_t001 |  |  |  |  |  |  |  |  |  |  |  |  |
| 0 | Vitvi18g02404\_t001 |  |  |  |  |  |  |  |  |  |  |  |  |
| 0 | Vitvi18g04794\_t001 |  |  |  |  |  |  |  |  |  |  |  |  |
| 0 | Vitvi18g02406\_t001 |  |  |  |  |  |  |  |  |  |  |  |  |
| 0 | Vitvi18g02407\_t001 |  |  |  |  |  |  |  |  |  |  |  |  |
| 0 | Vitvi18g02408\_t001 |  |  |  |  |  |  |  |  |  |  |  |  |
| 0 | Vitvi18g02409\_t001 |  |  |  |  |  |  |  |  |  |  |  |  |
| 0 | Vitvi18g02410\_t001 |  |  |  |  |  |  |  |  |  |  |  |  |
| 0 | Vitvi18g02411\_t001 |  |  |  |  |  |  |  |  |  |  |  |  |
| 0 | Vitvi18g02412\_t001 |  |  |  |  |  |  |  |  |  |  |  |  |
| 0 | Vitvi18g04795\_t001 |  |  |  |  |  |  |  |  |  |  |  |  |
| 0 | Vitvi18g02415\_t001 |  |  |  |  |  |  |  |  |  |  |  |  |
| 0 | Vitvi18g02416\_t001 |  |  |  |  |  |  |  |  |  |  |  |  |
| 0 | Vitvi18g02417\_t001 |  |  |  |  |  |  |  |  |  |  |  |  |
| 0 | Vitvi18g02418\_t001 |  |  |  |  |  |  |  |  |  |  |  |  |
| 0 | Vitvi18g04796\_t001 |  |  |  |  |  |  |  |  |  |  |  |  |
| 0 | Vitvi18g03271\_t001 |  |  |  |  |  |  |  |  |  |  |  |  |
| 0 | Vitvi18g04797\_t001 |  |  |  |  |  |  |  |  |  |  |  |  |
| 0 | Vitvi18g02420\_t001 |  |  |  |  |  |  |  |  |  |  |  |  |
| 0 | Vitvi18g02421\_t001 |  |  |  |  |  |  |  |  |  |  |  |  |
| 0 | Vitvi18g04798\_t001 |  |  |  |  |  |  |  |  |  |  |  |  |
| 0 | Vitvi18g02423\_t001 |  |  |  |  |  |  |  |  |  |  |  |  |
| 0 | Vitvi18g04799\_t001 |  |  |  |  |  |  |  |  |  |  |  |  |
| 0 | Vitvi18g04800\_t001 |  |  |  |  |  |  |  |  |  |  |  |  |
| 0 | Vitvi18g02424\_t001 |  |  |  |  |  |  |  |  |  |  |  |  |
| 0 | Vitvi18g02426\_t001 |  |  |  |  |  |  |  |  |  |  |  |  |
| 0 | Vitvi18g02427\_t001 |  |  |  |  |  |  |  |  |  |  |  |  |
| 0 | Vitvi18g02428\_t001 |  |  |  |  |  |  |  |  |  |  |  |  |
| 0 | Vitvi18g02429\_t002 |  |  |  |  |  |  |  |  |  |  |  |  |
| 0 | Vitvi18g02431\_t001 |  |  |  |  |  |  |  |  |  |  |  |  |
| 0 | Vitvi18g02432\_t001 |  |  |  |  |  |  |  |  |  |  |  |  |
| 0 | Vitvi18g02433\_t001 |  |  |  |  |  |  |  |  |  |  |  |  |
| 0 | Vitvi18g02434\_t001 |  |  |  |  |  |  |  |  |  |  |  |  |
| 0 | Vitvi18g02435\_t001 |  |  |  |  |  |  |  |  |  |  |  |  |
| 0 | Vitvi18g04801\_t001 |  |  |  |  |  |  |  |  |  |  |  |  |
| 0 | Vitvi18g04802\_t001 |  |  |  |  |  |  |  |  |  |  |  |  |
